# Acupuncture combined with language training for aphasia in children with cerebral palsy: a systematic review with meta-analysis and trial sequential analysis

**DOI:** 10.3389/fneur.2025.1502023

**Published:** 2025-03-12

**Authors:** Shuzhen Liu, Yujiao Li, Jun Chang, Jiangwei Shi, Lan Zhao

**Affiliations:** ^1^First Teaching Hospital of Tianjin University of Traditional Chinese Medicine, Tianjin, China; ^2^National Clinical Research Center for Chinese Medicine Acupuncture and Moxibustion, Tianjin, China

**Keywords:** acupuncture, language training, cerebral palsy, aphasia, meta-analysis, randomised controlled trials

## Abstract

**Objective:**

The aim of this study was to comprehensively evaluate the efficacy of acupuncture combined with language training in the treatment of aphasia in children with cerebral palsy (CP).

**Methods:**

We searched eight electronic databases from their inceptions to July 1, 2024 for randomized controlled trials (RCTs) of acupuncture for aphasia in children with CP. The evaluation of methodological quality for RCTs incorporated in this study adhered to the guidelines provided by the Cochrane risk-of-bias tool (ROB2). The Grading of Recommendations Assessment, Development and Evaluation Approach (GRADE) was used to evaluate the certainty of evidence of each outcome. The heterogeneity of the included literature was tested using Review Manager 5.4 software, while publication bias was estimated using funnel plots and Egger’s tests by STATA15.1. A trial sequential analysis (TSA) was performed to test the robustness of the conclusiveness of our results.

**Results:**

In this study, we encompassed a total of 56 randomised controlled trials encompassing 4,683 participants. The majority of these trials were characterized by either a high or uncertain risk of bias, predominantly due to the omission of blinding within their experimental setups. Meta-analysis showed that acupuncture combined with language training was significantly better than language training alone in improving the clinical efficiency (RR: 1.25; 95% CI: 1.21, 1.29; *p* < 0.00001). A subgroup analysis of the different types of acupuncture revealed that acupuncture, electroacupuncture, scalp acupuncture, and auricular point seed-pressing all showed a significant improvement in aphasia in children with CP. Acupuncture combined with language training could significantly improve the adaptive behaviour (MD: 7.46; 95% CI: 3.67, 11.26; *p* = 0.0001), verbal behaviour (MD: 7.79; 95% CI: 5.66, 9.92; *p* < 0.00001), fine motor behaviour (MD: 4.66; 95% CI: 1.28, 8.03; *p* = 0.007), and personal social behaviour (MD: 6.47; 95% CI: 2.38, 10.55; *p* = 0.002); it was also significantly more effective in improving the language comprehension developmental quotient (SMD: 2.02; 95% CI: 1.54, 2.50; *p* < 0.00001), the expressive language development quotient (SMD: 2.40; 95% CI: 1.76, 3.03; *p* < 0.00001), assessment of dysarthria (MD: 0.40; 95% CI: 0.11, 0.69; *p* = 0.007), and oral motor function (SMD: 2.63; 95% CI: 1.36, 3.90; *p* < 0.0001).

**Conclusion:**

Acupuncture combined with language training could be an effective treatment for aphasia in children with CP. Due to low or very low certainty of evidence and high heterogeneity, more rigorous RCTs are needed to verify the effect of acupuncture in the management of CP.

**Systematic review registration:**

https://www.crd.york.ac.uk/PROSPERO/view/CRD42024501328, identifier CRD42024501328.

## 1 Introduction

Cerebral palsy (CP) is a permanent central nervous system disorder that affects motor control and postural regulation in children. According to statistics, there are approximately 2–3 cases of CP per 1,000 live births worldwide (1). The prevalence rate of CP in China is reported to be 0.246 percent and with this number increasing by 30,000 to 80,000 per year (2). CP mainly manifests as motor dysfunction. In addition, CP may be accompanied by a variety of other comorbidities, of which aphasia is one of the common ones, with an incidence of approximately 80% (3). Aphasia not only affects the patient’s ability to communicate, but may also have a profound impact on their psychological development, social interactions, and overall quality of life. However, in clinical practice, motor dysfunction is often the focus of treatment, thus missing the optimal time for language and speech function treatment and intervention (4).

At present, no targeted medication exists for the treatment of language disorder manifestations in CP cases; language training is one of the main methods to treat language disorders in children with CP, using scientific language training to stimulate the language function of the child’s brain and achieve the role of promoting language development. However, the use of language rehabilitation training is relatively simple, with relatively poor efficacy and limitations in improving the disease prognosis (5).

Acupuncture, as an important part of traditional Chinese medicine (TCM), which has been practiced for over two thousand years to modulate body physiology via stimulation at specific body regions (acupoints) (6, 7). It has advantages such as simple operation, non-invasive treatment, and fewer adverse reactions, which can fully exert a therapeutic effect and have a positive effect on shortening the course of treatment (8). Acupuncture stimulates specific areas of the brain cortex, promotes synaptic regeneration, enhances brain compensatory function, helps alleviate brain cell damage, and protects neurons (9, 10). Recent studies have shown that acupuncture can improve the speech expression in children with CP by modulating the nerves (11, 12). However, there is no evidence-based medical evidence to prove whether acupuncture has an ameliorative effect on aphasia in children with CP. Therefore, this paper systematically reviews the research data in recent years, aiming to study the effectiveness of acupuncture in improving aphasia in children with CP more objectively through meta-analysis, with a view to providing evidence for clinical medical research.

## 2 Methods

Adhering to the PRISMA extension guidelines (13), this research was executed. Our systematic review methodology was registered in advance with PROSPERO under the identifier CRD42024501328, discoverable at https://www.crd.york.ac.uk/prospero/.

### 2.1 Data sources and search strategy

Our search encompassed a range of pertinent databases, including PubMed, Embase, the Cochrane Library, Web of Science, along with Chinese databases including the China Knowledge Infrastructure (CNKI), Wan-Fang Database, China Science Journal Database (VIP), and SinoMed, spanning from the inception of these databases up to the date of July 1, 2024. The main search terms were “Acupuncture,” “Electroacupuncture,” “Cerebral palsy,” “Aphasia,” “Speech Disorder,” “Dysarthria,” “Language Development Disorder,” and “randomized controlled trial.” The comprehensive search methodology is detailed in the Supplementary File 1.

### 2.2 Inclusion criteria

#### 2.2.1 Types of studies

Eligible for inclusion in our study were RCTs that investigated the acupuncture combined with language training for aphasia among children diagnosed with CP. The term “random” in these trials pertains to the allocation process, which may or may not have incorporated blinding.

#### 2.2.2 Participants

The study involved children diagnosed with CP as well as those who fulfilled the criteria for aphasia. The selection of participants was unrestricted by gender or nationality.

#### 2.2.3 Interventions

In the control arm of the study, participants were exclusively provided with language training. Conversely, the treatment arm included an integrated approach, offering both acupuncture and language training to the patients. Acupuncture therapy included body acupuncture, electroacupuncture, warming acupuncture, catgut implantation at acupoint, auricular pressure beans, and acupoint injection. The study imposed no limitations regarding the precise timing of interventions, selection of acupuncture points, or duration of treatment protocols.

#### 2.2.4 Outcomes

The primary outcomes include clinical effectiveness rate and development quotient (adaptive behaviour, gross motor behaviour, verbal behaviour, fine motor behaviour, and personal social behaviour), while the secondary outcomes include assessment of dysarthria, oral motor function, expressive language development quotient, and language comprehension developmental quotient. According to the evaluation standard of the China Code for the Diagnosis and Treatment of Rehabilitation Medicine from the Department of Medical Administration of the People’s Republic of China: (1) Significant effects: language development delay, language development and understanding ability to improve the two stages, and language expression ability improvement; (2) Effective: language development delay, language development understanding ability to improve a stage, and language expression ability has improved; (3) Invalid: the improvement in language delay was not obvious. Clinical effectiveness rate = (basic cure + significant + effective)/100% of total cases. The development of children was evaluated using the Gesell development scale, which includes the following five aspects: adaptive behaviour, gross motor behaviour, verbal behaviour, fine motor behaviour, and personal social behaviour. The lower the score from this scale, the less ideal the development status is.

### 2.3 Exclusion criteria

The criteria for exclusion were delineated as follows: (1) for repetitive articles, keep only the most recent or comprehensive ones; (2) meta-analyses, retrospective investigations, case reports, animal experiments, non-RCTs, non-English and Chinese papers were excluded; (3) interventions that do not meet the requirements, as well as diseases that do not match those in this study, are excluded; (4) articles that could not be obtained were excluded; (5) articles with non-compliant research subjects were excluded.

### 2.4 Selection criteria

The literature was independently evaluated by two researchers against the established inclusion and exclusion criteria, employing the PICOS to guide the selection process.

### 2.5 Data screening and extraction

For the purpose of pinpointing studies compliant with the inclusion criteria, all included studies were uploaded into Endnote 20. Two reviewers, each with specialized training (SZL and YJL), scrutinized each study individually, and removed duplicates studies, and studies that failed to satisfy the inclusion criteria. Data extraction encompassed pertinent details: authorship, year of publication, age, gender, the size of the study sample, the duration of the treatment regimen, the methodology of intervention, and outcome indicators. Upon completion of their individual reviews, the two reviewers performed a cross-verification to confirm the data’s veracity. In instances where discrepancies arose between the reviewers’ assessments or data extractions, a third, neutral assessor (JC) was consulted to reach a consensus.

### 2.6 Risk of bias

The evaluation of the methodological quality within the selected RCTs was guided by the Cochrane risk-of-bias tool (ROB2). This assessment was independently carried out by two reviewers (YJL and Y-JL), using Covidence to ensure blinding (14). For any risk domains classified as serious, critical, or lacking information, the reviewers provided detailed justifications. When essential details were not readily available in the reports under review, the reviewers sought and verified information from study protocols, clinical trial registries, and any supplementary documents provided. To resolve any inconsistencies in the assessment, the reviewers engaged in discussions and strived to reach a mutual agreement on the evaluation of each risk domain within the ROB2 tool. In cases where a consensus was not achieved, a third reviewer (JC), was invited to participate in the assessment, ensuring a unified decision was made. ROB2 was conducted at the outcome level.

### 2.7 GRADE assessment

Furthermore, the certainty of evidence derived from the synthesis of the primary outcomes was appraised using the Grading of Recommendations Assessment, Development, and Evaluation (GRADE) framework (15). For each primary outcome within the network estimate, an evaluation was conducted considering several criteria, including risk of bias, indirectness, inconsistency, imprecision, and publication bias. In accordance with the GRADE framework, the level of evidence was adjusted; it was lowered one tier for issues deemed “serious” and two tiers for those considered “very serious.” Ultimately, an integrated evaluation of the evidence’s certainty was formulated, with each comparison receiving an overall qualitative rating. This rating was based on a four-tiered system of evidence quality: high, moderate, low, and very low.

### 2.8 Data analysis

Data analysis was performed utilizing the Cochrane Collaboration Meta-analysis software (Review Manager 5.4). For dichotomous data, the relative risk (RR) along with its 95% confidence interval (CI) was calculated, whereas for continuous data, the weighted mean difference (WMD) or standard mean difference (SMD) and their corresponding 95% CI were reported. Statistical significance was set at a *p*-value of less than 0.05. A heterogeneity test was carried out on the included literature. If *p* ≤ 0.1 and *I*^2^ ≥ 50%, it indicated the existence of heterogeneity; the random effect model was used for heterogeneity analysis. Otherwise, a fixed-effects model was used. Given the potential impact of acupuncture type on treatment efficacy, a subgroup analysis was conducted to examine the effectiveness of different acupuncture modalities in treating aphasia in children with CP. Additionally, considering the possible influence of treatment duration, subgroup analyses were performed to compare the efficacy of treatments lasting up to 3 months versus those exceeding this period. A sensitivity analysis was conducted to evaluate the robustness of the findings by sequentially excluding each study and scrutinizing the impact on both the aggregated effectiveness of the remaining studies and the overall efficacy. Furthermore, Egger’s test was applied to determine the presence of publication bias.

To control the risks associated with type I and type II errors, our study implemented a trial sequential analysis (TSA) to assess the clinical efficacy of acupuncture in conjunction with language training for aphasia in children with CP. Utilizing the TSA software, version 0.9.5.10 beta, we adjusted the confidence intervals (CIs) in response to the sparse data and the issue of repeated testing within the cumulative meta-analysis. A conclusive determination is attainable if the cumulative *Z*-curve surpasses the TSA boundary or intersects with the Required Information Size line, indicating that additional research may not be warranted (16). TSA was performed at an overall 10% risk level of a type I error and with 80% power.

## 3 Results

### 3.1 Literature selection

**Figure 1:**
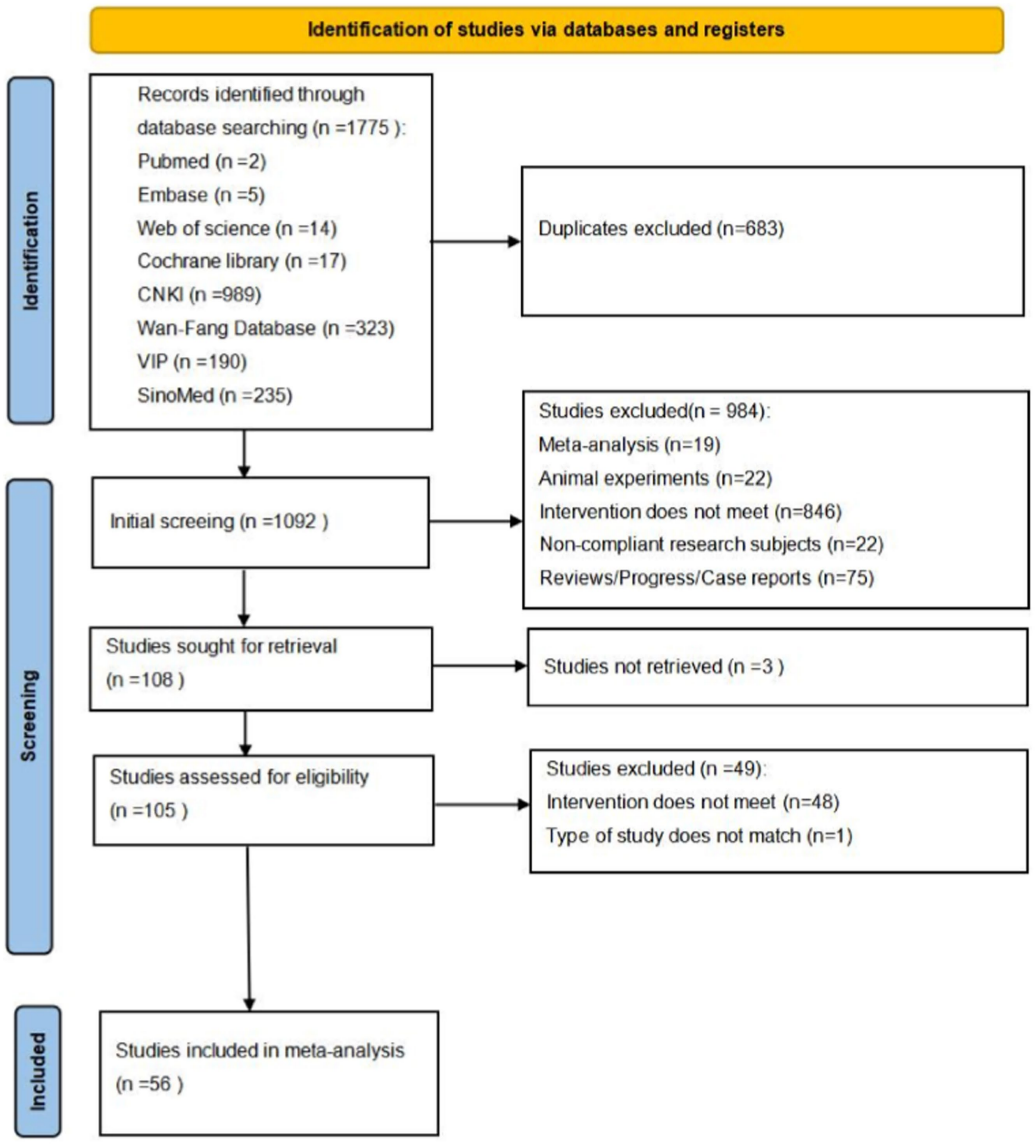
Flow diagram of searching and articles selection.

We searched a total of 1,775 articles from eight electronic databases. Following the removal of duplicate studies, 1,092 relevant studies were identified. Upon reviewing the titles and abstracts, we narrowed down the selection to 108 studies that appeared to be pertinent to our investigation. Subsequently, two reviewers, working independently, conducted a thorough examination of the full texts of the studies, who conducted an additional eligibility assessment according to the predefined inclusion and exclusion criteria. Ultimately, 56 studies were deemed suitable for inclusion in the final meta-analysis, with all trials being published in Chinese. The comprehensive process of literature screening is depicted in Figure 1.

### 3.2 Characteristics of the included literature

This study encompassed 56 articles, each a randomized controlled trial originating from a single center within China. The English titles, authors and abstracts of all the articles are included in Supplementary File 2. Collectively, these trials enrolled a total of 4,683 patients, with 2,371 allocated to the experimental group and 2,312 to the control group (17–72), and the length of the treatment course ranged from 1 to 9 months. Incomplete baseline feature details were identified in several studies (17, 19, 25, 42, 48, 68), but no significant differences were observed between groups among the characteristics (*p* > 0.05). Of the 56 studies, 39 used body acupuncture (17–19, 21, 25, 26, 28–30, 33–38, 40–48, 50–52, 55, 58–62, 65–68, 71, 72), 13 used scalp acupuncture (20, 22–24, 27, 32, 39, 49, 53, 54, 56, 69, 70), one used laser acupuncture (31), one used auricular point seed-pressing (59), and three used electroacupuncture (25, 57, 64) [among them, Zou et al. (25) had two experimental groups-one used acupuncture and the other used electroacupuncture]. In addition, only one study (25) that reported on the safety of acupuncture. A summary of the fundamental characteristics of the included studies can be found in Table 1.

**Table 1:** The basic characteristics of the included RCTs.

Study ID	Patients N		Average age (years)		Sex		Intervention		Treatment (days)	Outcomes
	E	C	E	C	M	F	E	C		
Li (17)	30	30	NR		NR		Acupuncture	Language training	60 days	①
Liu et al. (18)	38	38	2–7		20	18	Acupuncture	Language training	10 days × 3	① ④
					28	10				
Li (19)	30	30	NR		47	13	Acupuncture	Language training	60 days	①
Yang (20)	49	76	0.8–7		28	21	Scalp acupuncture	Language training	90 days	①
					42	34				
Jiang (21)	47	46	2–6		51	42	Acupuncture	Language training	20 days × 4	①
Li et al. (22)	31	30	1–6		37	24	Scalp acupuncture	Language training	6 days × 12	①
Li et al. (23)	49	76	3.4 ± 1.2		28	21	Scalp acupuncture	Language training	15 days × 5	①
					42	34				
Liang et al. (24)	31	30	3.6 ± 0.6		37	24	Scalp acupuncture	Language training	20 days × 3	① ⑦ ⑧ ⑨
Zou (electroacupuncture) et al. (25)	40	39	1–7		NR		Electroacupuncture	Language training	20 days × 6	① ④
Zou (acupuncture) et al. (25)	42	39	1–7		NR		Acupuncture	Language training	20 days × 6	① ④
Fan and Yang (26)	50	50	1–6		51	49	Acupuncture	Language training	21 days × 8	①
Jin et al. (27)	64	57	3.9 ± 0.6	4.3 ± 0.7	67	54	Scalp acupuncture	Language training	28 days × 6	①
Meng and Zhou (28)	15	15	3	5	11	4	Acupuncture	Language training	70 days	①
					9	6				
Yang (29)	15	15	3.1 ± 1.1	5.1 ± 1.1	9	6	Acupuncture	Language training	30 days	①
					4	11				
Li et al. (30)	45	45	6.3 ± 2.4	6.5 ± 2.5	21	24	Acupuncture	Language training	30 days × 3	①
					23	22				
Li et al. (31)	20	20	4.12 ± 2.15	4.56 ± 1.95	13	7	Laser acupuncture	Language training	20 days × 3	① ④
					12	8				
Li (32)	80	80	2.8 ± 1.5		88	72	Scalp acupuncture	Language training	30 days × 6	①
Liu and Shi (33)	30	30	2–6		36	24	Acupuncture	Language training	30 days × 3	① ⑦ ⑧ ⑨
Wang et al. (34)	30	30	1.89 ± 0.62	2.16 ± 0.57	22	8	Acupuncture	Language training	90 days × 2	①
					24	6				
Zhang (35)	45	45	3	5	20	25	Acupuncture	Language training	56 days × 3	①
					30	15				
Wang (36)	44	44	4.87 ± 1.12	4.62 ± 1.03	24	20	Acupuncture	Language training	90 days	① ⑧ ⑨
					26	18				
Yu (37)	31	30	4 ± 1.2	3.5 ± 1	15	16	Acupuncture	Language training	90 days	①
					17	13				
Guo et al. (38)	41	40	3.34 ± 1.45	3.61 ± 1.69	25	16	Acupuncture	Language training	10 days × 9	① ②
					27	13				
Li et al. (39)	60	60	3.52 ± 1.24	3.55 ± 1.21	36	24	Scalp acupuncture	Language training	15 days × 5	①
					35	25				
Liao (40)	39	39	2.38 ± 0.94	2.52 ± 0.91	21	18	Acupuncture	Language training	180 days	① ② ④ ⑥ ⑩
					20	19				
Qin and Li (41)	45	45	2–10		21	24	Acupuncture	Language training	30 days × 3	①
					23	22				
Tao and Ding (42)	30	30	2–10		NR		Acupuncture	Language training	20 days × 5	①
Du et al. (43)	68	68	2.3 ± 1.2	2.1 ± 1.3	41	27	Acupuncture	Language training	90 days	⑦ ⑧
					43	25				
Liu et al. (44)	30	30	3.28 ± 0.76	3.02 ± 0.78	17	13	Acupuncture	Language training	10 days × 9	④
					16	14				
Shao (45)	39	40	2.24 ± 1.17	2.53 ± 1.46	30	9	Acupuncture	Language training	90 days × 3	①
					32	8				
Zhang (46)	38	38	2.9 ± 1.2	3.2 ± 0.9	21	17	Acupuncture	Language training	90 days	①
					20	18				
Zhao (47)	30	30	3.52 ± 1.38	3.87 ± 1.52	22	8	Acupuncture	Language training	90 days	①
					20	10				
Bao (48)	39	40	NR		NR		Acupuncture	Language training	90 days × 3	①
Chen (49)	50	50	4.29 ± 0.86	4.17 ± 0.92	28	22	Scalp acupuncture	Language training	90 days	①
					27	23				
Chen (50)	47	47	2.14 ± 0.82	3.28 ± 0.89	25	22	Acupuncture	Language training	180 days	①
					24	23				
Wu et al. (51)	38	38	7.35 ± 3.32	7.40 ± 3.82	22	16	Acupuncture	Language training	90 days	① ⑦ ⑧ ⑨
					21	17				
Zhao et al. (52)	49	49	4.51 ± 1.36	4.82 ± 1.17	28	21	Acupuncture	Language training	90 days	① ④ ⑨
					25	24				
Ai et al. (53)	15	15	3.8		18	12	Scalp acupuncture	Language training	10 days × 12	①
Huang et al. (54)	33	33	4.37 ± 1.25	4.58 ± 1.45	18	15	Scalp acupuncture	Language training	28 days × 6	⑦ ⑧ ⑩
					19	14				
Jiao (55)	56	55	4.27 ± 0.26	4.20 ± 0.11	28	28	Acupuncture	Language training	90 days	① ② ③ ④ ⑤ ⑥ ⑦ ⑧
					27	28				
Li (56)	40	40	4.05 ± 1.01	4.03 ± 1.02	23	17	Scalp acupuncture	Language training	90 days	①
					22	18				
Yuan et al. (57)	30	30	5.45 ± 0.33	5.39 ± 0.42	13	17	Electroacupuncture	Language training	30 days	① ② ④
					12	18				
Lian (58)	40	40	2.9 ± 1.3	3.0 ± 1.2	22	18	Acupuncture	Language training	30 days × 6	① ④
					23	17				
Lin et al. (59)	43	43	30.8 ± 2.6	30.4 ± 1.8	21	22	Auricular point seed-pressing	Language training	90 days	① ② ③ ④ ⑤ ⑥
					23	20				
Liu (60)	75	75	3.5 ± 1.5	4.0 ± 2.0	41	34	Acupuncture	Language training	180 days	⑩
					40	35				
Liu et al. (61)	54	53	3.72 ± 0.91	3.82 ± 0.74	32	22	Acupuncture	Language training	90 days	①
					30	23				
Ma (62)	40	40	2.9 ± 0.9	2.7 ± 0.8	21	19	Acupuncture	Language training	84 days	① ⑦ ⑧
					22	18				
Song (63)	33	33	4.23 ± 1.24	4.44 ± 1.01	17	16	Acupuncture	Language training	90 days	① ② ④ ⑥ ⑩
					20	13				
Yu (64)	24	24	2.47 ± 0.73	2.23 ± 0.64	16	8	Electroacupuncture	Language training	90 days	①
					17	7				
Qiu (65)	30	30	2.39 ± 0.68	2.42 ± 0.71	17	13	Acupuncture	Language training	180 days	① ② ④ ⑥ ⑩
					19	11				
Wang (66)	35	35	3.81 ± 1.52	3.88 ± 1.53	19	16	Acupuncture	Language training	90 days	①
					20	15				
Yan et al. (67)	30	30	7.35 ± 1.22	7.14 ± 1.31	17	13	Acupuncture	Language training	60 days	② ③ ④ ⑤ ⑥ ⑨
					18	12				
Yu (68)	30	30	1–6		35	25	Acupuncture	Language training	NR	①
Jin and Huang (69)	124	66	2.30 ± 1.34	2.24 ± 1.17	102	22	Scalp acupuncture	Language training	28 days × 6	①
					51	15				
Yang and Liu (70)	50	50	3.98 ± 0.51	4.01 ± 0.63	30	20	Scalp acupuncture	Language training	30 days × 3	① ② ④ ⑥ ⑦ ⑧
					29	21				
Zhang et al. (71)	46	46	3.42 ± 1.02	3.53 ± 1.05	24	22	Acupuncture	Language training	90 days	① ④
					25	21				
Yang and Bai (72)	44	44	4.81 ± 0.52	4.78 ± 0.46	21	23	Acupuncture	Language training	90 days	①
					23	21				

E, experimental group; C, control group; M, male; F, female; NR, not reported; ① clinical effectiveness rate; ② adaptive behaviour; ③ gross motor behaviour; ④ verbal behaviour; ⑤ fine motor behaviour; ⑥ personal social behaviour; ⑦ language comprehension developmental quotient; ⑧ expressive language development quotient; ⑨ assessment of dysarthria; ⑩ oral motor function.

### 3.3 Risk of study bias

**Figure 2:**
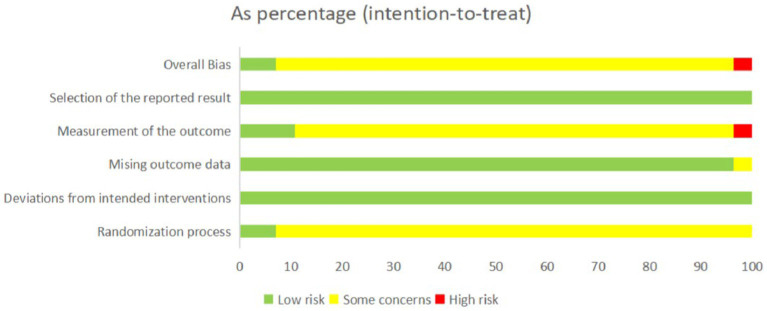
Risk of bias graph.

**Figure 3:**
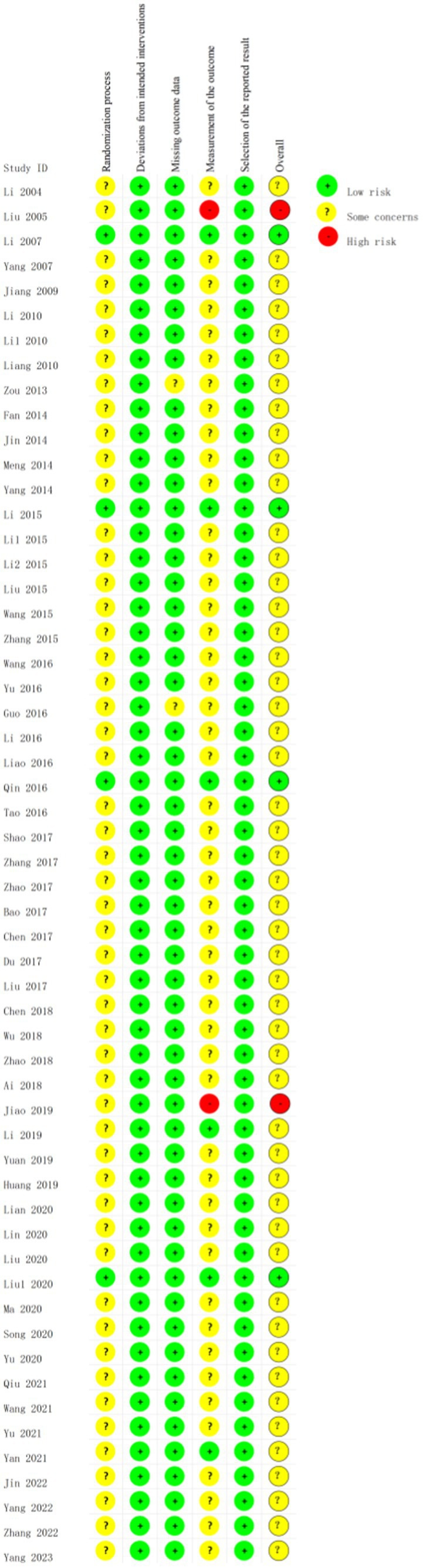
Risk of bias summary.

We utilised the revised Cochrane risk-of-bias tool (ROB 2.0) to evaluate the risk of bias and the quality of the included studies. Four studies’ outcomes were rated as low risk (19, 30, 41, 61), two studies’ outcomes were rated as high risk (18, 55), and the results of the other studies were rated as having some concerns (Figures 2, 3). All trials mentioned randomisation; Nonetheless, specifics regarding the randomization were reported in merely four RCTs (19, 30, 41, 61), which consequently earned a low risk designation. Notably, among these, two employed a random number table for their randomization process (30, 41), while the other pair adopted a single-blind, randomized controlled methodology (19, 61). In terms of measuring the outcome, there are two studies (18, 55) in which the evaluators were aware of the intervention received by the study participants; as such, the assessment of the outcome had been influenced by this knowledge and these trials were, therefore, rated as high risk. In addition, there are six studies (19, 30, 41, 56, 61, 67) in which the evaluators were unaware of the intervention that the study participants received, these trials were rated as low risk.

### 3.4 Certainty of evidence

**Figure 4:**
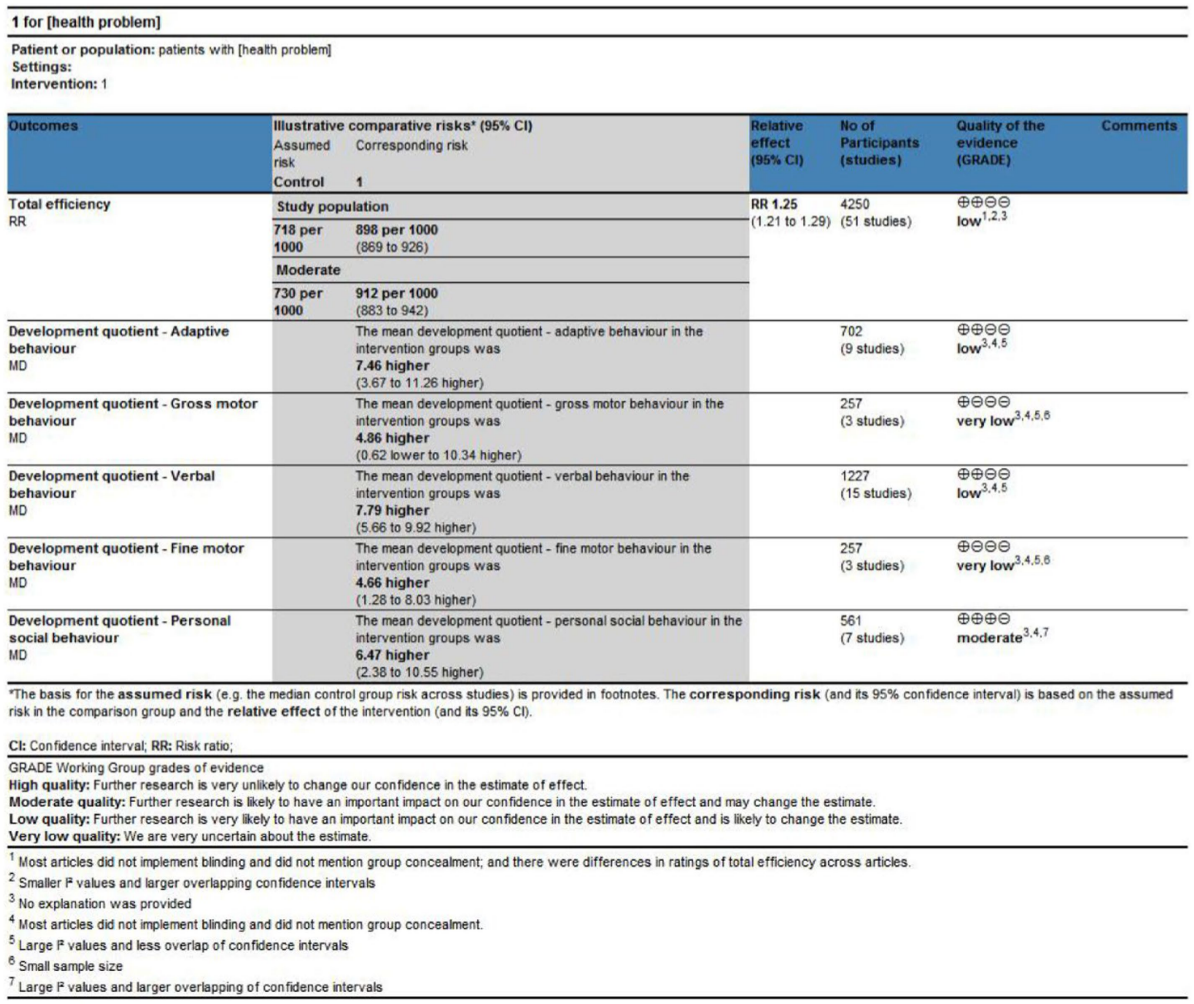
GRADE assessment of meta-analytic results.

The overall certainty of the evidence was rated for meta-analytic outcomes as being moderate to very low. The principal factors that led to the reduction in the evidence rating included concerns related to risk of bias, inconsistency, and imprecision. However, the assessment of indirectness did not undergo demotion, given that this systematic review adhered to stringent parameters for the selection of population, intervention, comparison, and outcome criteria. Evidence of very low certainty indicated that acupuncture combined with language training had no effect on improving either gross or fine motor behaviour. Figure 4 presents a summary of the results, with footnotes explaining the downgrade judgments (decreasing the rating of the certainty of the evidence).

## 4 Outcomes

### 4.1 The clinical effectiveness rate

**Figure 5:**
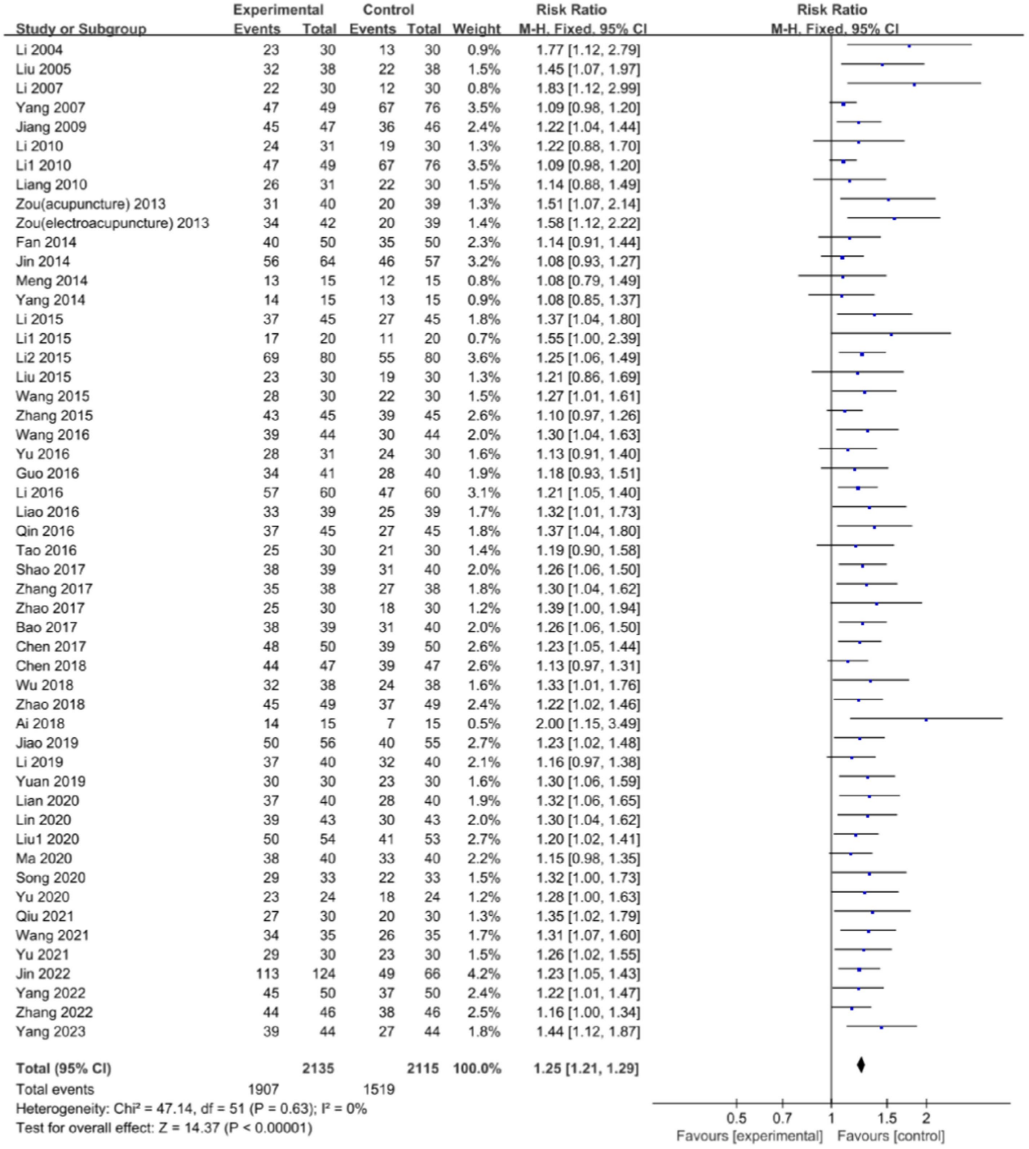
Forest plot of the clinical effectiveness rate comparison between acupuncture and control group.

A total of 51 studies (17–42, 45–53, 55–59, 61–66, 68–72) reported the clinical effectiveness rate [among them, Zou et al. (25) had two experimental groups-one used acupuncture and the other used electroacupuncture] and there was no significant heterogeneity between them (*p* = 0.63, *I*^2^ = 0%). Utilizing the fixed-effects model, a significant disparity was observed in the clinical effectiveness rate between the integration of acupuncture with language training and language training alone (RR: 1.25; 95% CI: 1.21, 1.29; *p* < 0.00001). This finding underscores the potential of acupuncture, when combined with language training, to improve the clinical efficacy of treating aphasia in patients with CP, as shown in Figure 5.

### 4.2 GESELL development scale

The GESELL development scale consists of five main dimensions, which are adaptive behaviour, gross motor behaviour, verbal behaviour, fine motor behaviour, and personal social behaviour.

**Figure 6:**
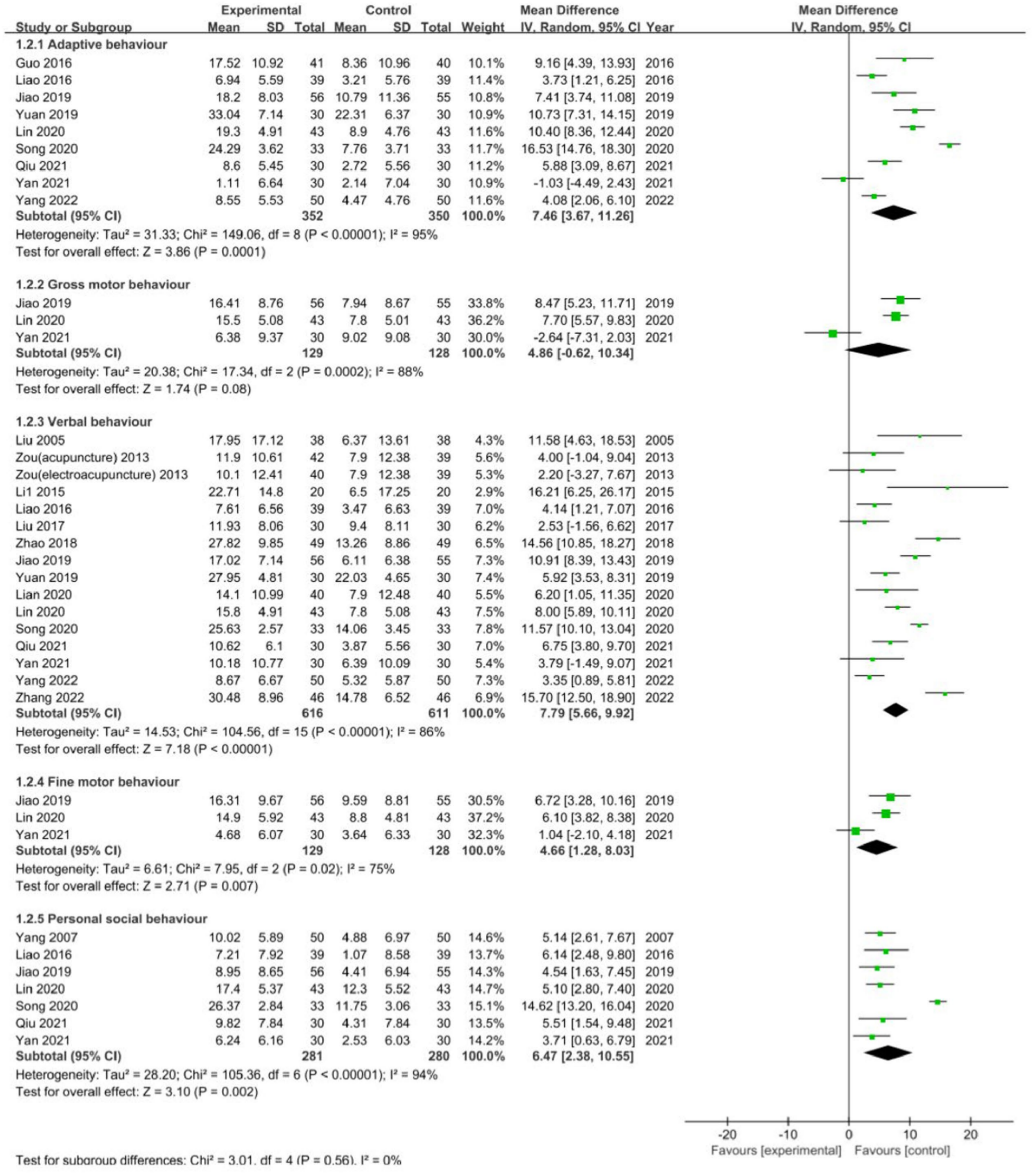
Forest plot of GESELL development scale comparison between acupuncture and control group.

Adaptive behaviour was reported in nine studies (38, 40, 55, 57, 59, 63, 65, 67, 70), and the statistical data revealed that acupuncture combined with language training significantly influenced adaptive behaviour in aphasic children with CP (MD: 7.46; 95% CI: 3.67, 11.26; *p* = 0.0001; heterogeneity: *I*^2^ = 88%; *p* < 0.00001; Figure 6), which indicated that this treatment method significantly improves adaptive behaviour compared to treatment with language training alone. Three studies (55, 59, 67) reported results showing no significant difference in gross motor behaviour between treatment using acupuncture combined with language training and treatment using language training alone (MD: 4.86; 95% CI: −0.62, 10.34; *p* = 0.08; heterogeneity: *I*^2^ = 88%; *p* = 0.0002; Figure 6).

Verbal behaviour was reported in 15 studies (18, 25, 31, 40, 44, 52, 55, 57–59, 63, 65, 67, 70, 71) [among them, Zou et al. (25) had two experimental groups]; totally 132 CP children were randomly assigned to the speech training group (Group A, 44 cases), the electroacupuncture combined speech training group (Group B, 44 cases), and the acupuncture combined speech training group (Group C, 44 cases). Patients in Group A received one to one training including game therapy, therapy of communication attitudes, and so on. Those in the other two groups were needled at Baihui (GV20), Sishencong (EX-HN1), the first language zone, the second language zone, and the third language zone. Those in Group B were treated with electric needling and then speech training. Those in Group C were treated with language training, while needling with needle maintaining for 40 min. All patients were treated once daily, 5 times per week, 20 times as one course of treatment, six courses in total, and the results indicated that acupuncture combined with language training produced a notably positive impact on verbal behaviour in aphasic children with CP (MD: 7.79; 95% CI: 5.66, 9.92; *p* < 0.00001; heterogeneity: *I*^2^ = 86%; *p* < 0.00001; Figure 6), which indicated that this treatment method significantly improves verbal behaviour compared to treatment with language training alone. Statistics from the three studies (55, 59, 67) showed that acupuncture combined with language training exerted a significant influence on the fine motor behaviour in aphasic children with CP (MD: 4.66; 95% CI: 1.28, 8.03; *p* = 0.007; heterogeneity: *I*^2^ = 75%; *p* = 0.02; Figure 6), which indicated that this method of treatment could significantly improve this type of behaviour compared to treatment with language training alone. Seven studies (40, 55, 59, 63, 65, 67, 70) reported statistical data showing that acupuncture combined with language training significantly impacted the personal social behaviour in the treatment of aphasia in children with CP (MD: 6.47; 95% CI: 2.38, 10.55; *p* = 0.002; heterogeneity: *I*^2^ = 94%; *p* < 0.00001; Figure 6), which indicated that this method of treatment could significantly improve personal social behaviour in aphasic children with CP compared to treatment using language training alone.

### 4.3 Language comprehension developmental quotient

**Figure 7:**
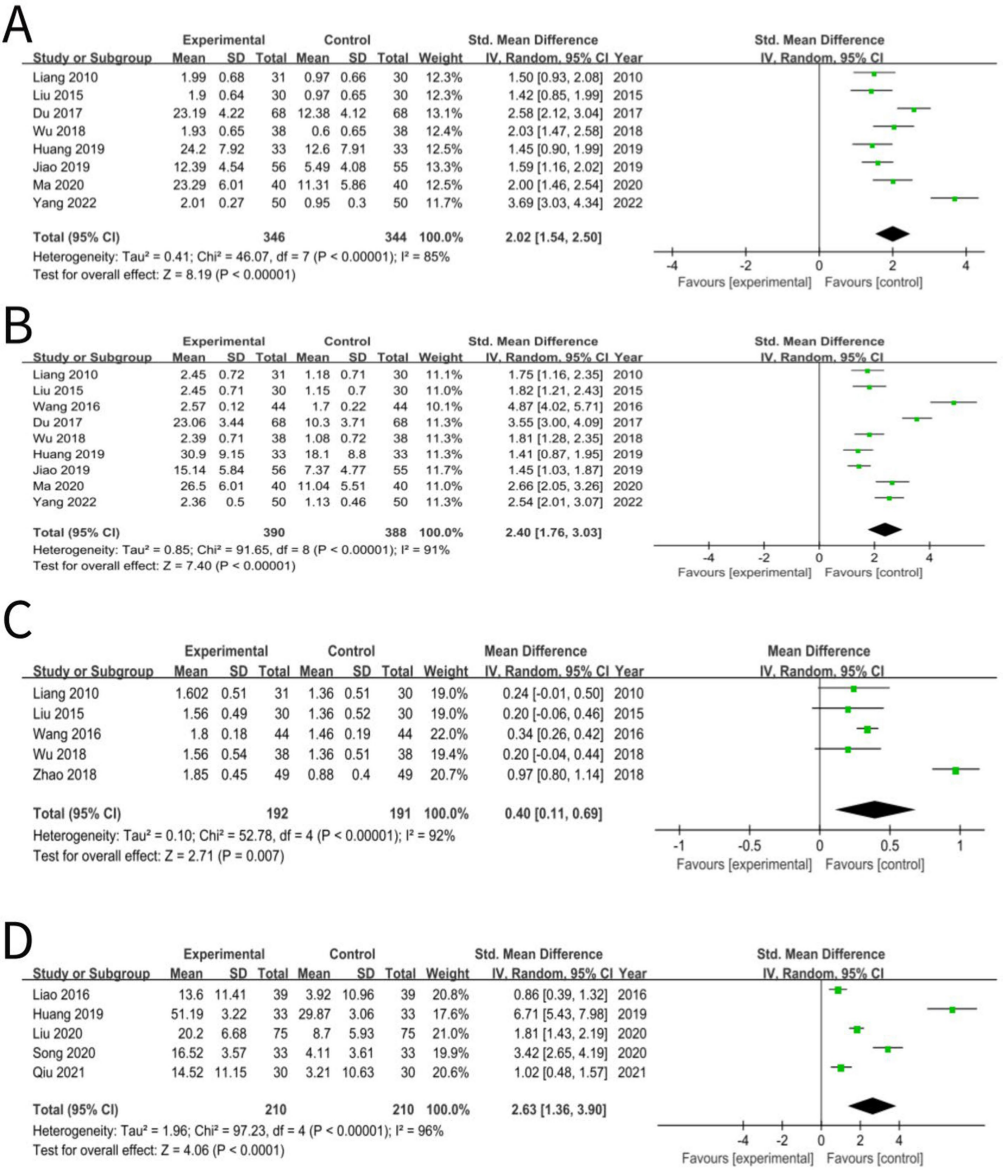
**(A)** Forest plot of language comprehension developmental quotient comparison between acupuncture and control group. **(B)** Forest plot of expressive language development quotient comparison between acupuncture and control group. **(C)** Forest plot of assessment of dysarthria comparison between acupuncture and control group. **(D)** Forest plot of oral motor function comparison between acupuncture and control group.

The language comprehension developmental quotient was reported in eight studies (24, 33, 43, 51, 54, 55, 62, 70) and the statistical analysis revealed that the integration of acupuncture with language training yielded a significant impact on this quotient of aphasic children with CP (SMD: 2.02; 95% CI: 1.54, 2.50; *p* < 0.00001; heterogeneity: *I*^2^ = 85%; *p* < 0.00001; Figure 7A), which indicated that this method of treatment can significantly improve the language comprehension developmental quotient compared to treatment using language training alone.

### 4.4 Expressive language development quotient

Nine studies (24, 33, 36, 43, 51, 54, 55, 62, 70) reported the expressive language development quotient and the analysis of statistical data indicated that the combination of acupuncture and language training significantly influenced this quotient among children with CP (SMD: 2.40; 95% CI: 1.76, 3.03; *p* < 0.00001; heterogeneity: *I*^2^ = 91%; *p* < 0.00001; Figure 7B), which indicated that this treatment method could improve the expressive language development quotient more so than treatment with language training alone.

### 4.5 Assessment of dysarthria

An assessment of dysarthria was reported in five studies (24, 33, 36, 51, 52) and the results showed that acupuncture combined with language training exerted a pronounced beneficial effect on dysarthria in aphasic children with CP (MD: 0.40; 95% CI: 0.11, 0.69; *p* = 0.007; heterogeneity: *I*^2^ = 92%; *p* < 0.00001; Figure 7C), which indicated that this method of treatment could improve dysarthria more so than treatment using language training alone.

### 4.6 Oral motor function

Five studies (40, 54, 60, 63, 65) reported oral motor function and the statistical outcomes demonstrated a notable improvement in this characteristic of children with CP who suffered from aphasia, attributed to the concurrent application of acupuncture and language training (SMD: 2.63; 95% CI: 1.36, 3.90; *p* < 0.0001; heterogeneity: *I*^2^ = 96%; *p* < 0.00001; Figure 7D), which indicated that this treatment method could improve the oral motor function more so than using treatment with language training alone.

### 4.7 Subgroup analysis

#### 4.7.1 Analysis of the efficacy of different types of acupuncture

**Figure 8:**
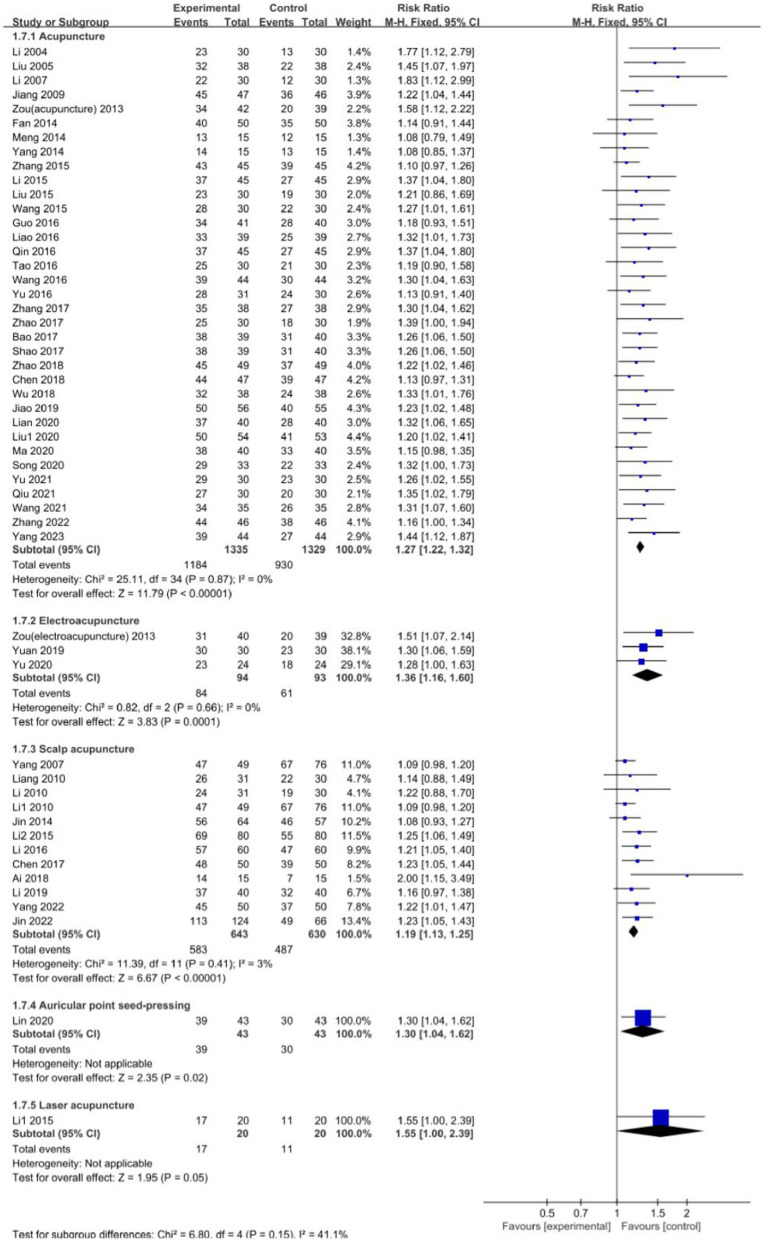
Forest plot comparing the effects of different acupuncture types.

Figure 8 shows a subgroup analysis of the effect of different types of acupuncture on the efficacy of aphasia treatment in children with CP. Among them, 35 studies (17–19, 21, 25, 26, 28–30, 33–38, 40–42, 45–48, 50–52, 55, 58, 61–63, 65, 66, 68, 71, 72) used acupuncture as the intervention measure, three studies (25, 57, 64) used electroacupuncture, and 12 studies (20, 22–24, 27, 32, 39, 49, 53, 56, 69, 70) used scalp acupuncture. In addition, the intervention measures of auricular point seed-pressing (59) and laser acupuncture (31) were each discussed in one study. The results showed that acupuncture (RR: 1.27; 95% CI: 1.22, 1.32; *p* < 0.00001; *I*^2^ = 0%), electroacupuncture (RR: 1.36; 95% CI: 1.16, 1.60; *p* = 0.0001; *I*^2^ = 0%), scalp acupuncture (RR = 1.19; 95% CI: 1.13, 1.25; *p* < 0.00001; *I*^2^ = 3%), and auricular point seed-pressing (RR: 1.30; 95% CI: 1.04, 1.62; *p* = 0.02) significantly improved aphasia in children with CP more so than when compared to treatment without acupuncture. However, the effect of laser acupuncture on the treatment of aphasia in children with CP is limited and there was no significant difference compared with treatment using language training alone (RR: 1.55; 95% CI: 1.00, 2.39; *p* = 0.05).

#### 4.7.2 Analysis of the efficacy of different treatment courses

**Figure 9:**
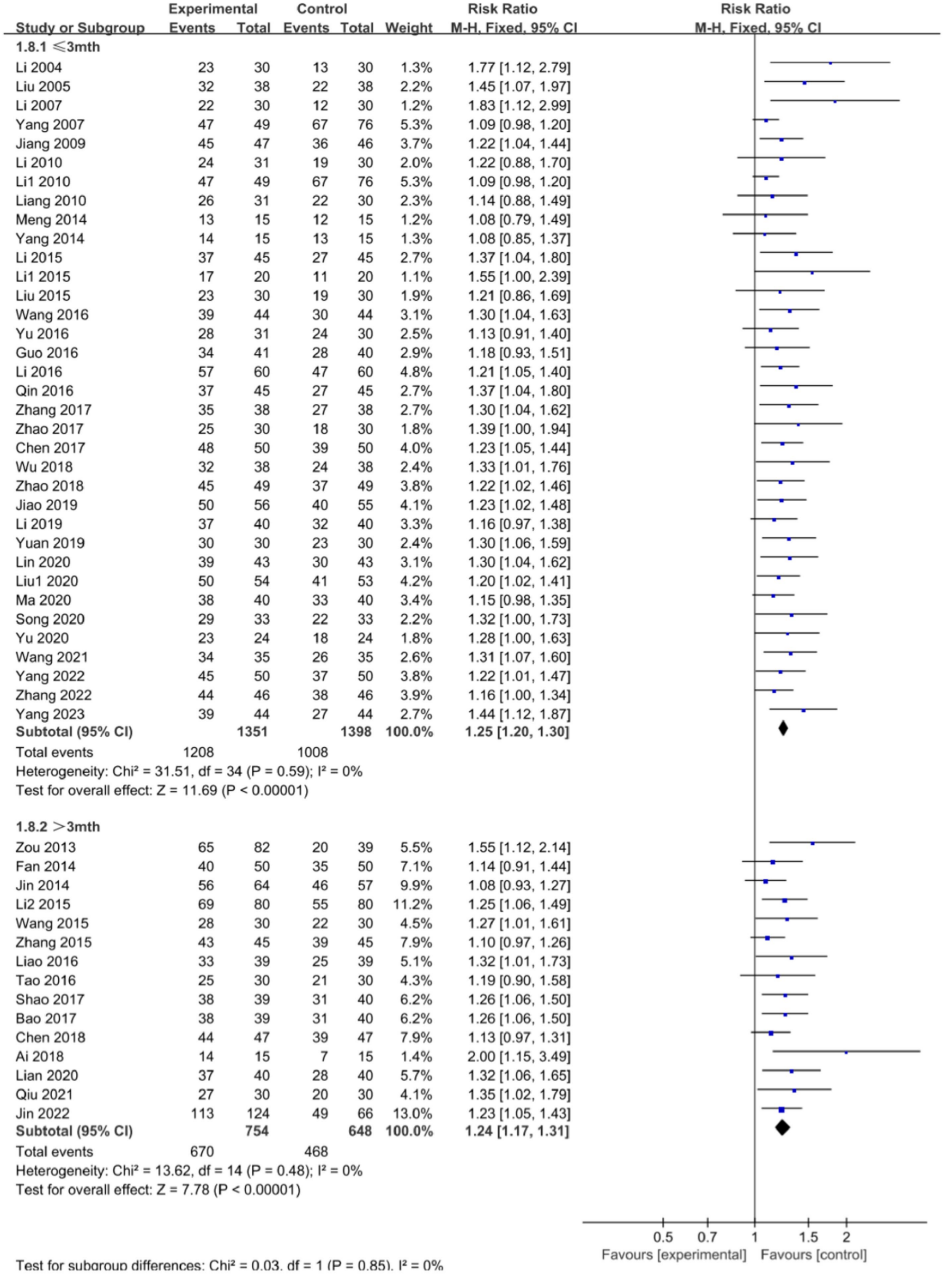
Forest plot comparing the efficacy of different courses of treatment.

Figure 9 shows a subgroup analysis of the effect of different treatment courses on the efficacy of aphasia treatment in children with CP. Among them, 35 studies (17–24, 28–31, 33, 36–39, 41, 46, 47, 49, 51, 52, 55–57, 59, 61–64, 66, 70–72) had a course of treatment of ≤3 months, and 15 studies (25–27, 32, 34, 35, 40, 42, 45, 48, 50, 53, 58, 65, 69) had a course of treatment of >3 months. The results showed significant improvement in aphasia in patients with acupuncture sessions ≤3 months (RR: 1.25; 95% CI: 1.20, 1.30; *p* < 0.00001; *I*^2^ = 0%) and in patients with sessions >3 months (RR: 1.24; 95% CI: 1.17, 1.31; *p* < 0.00001; *I*^2^ = 0%) compared with patients who did not receive acupuncture.

### 4.8 Sensitivity analysis and publication Bias

**Figure 10:**
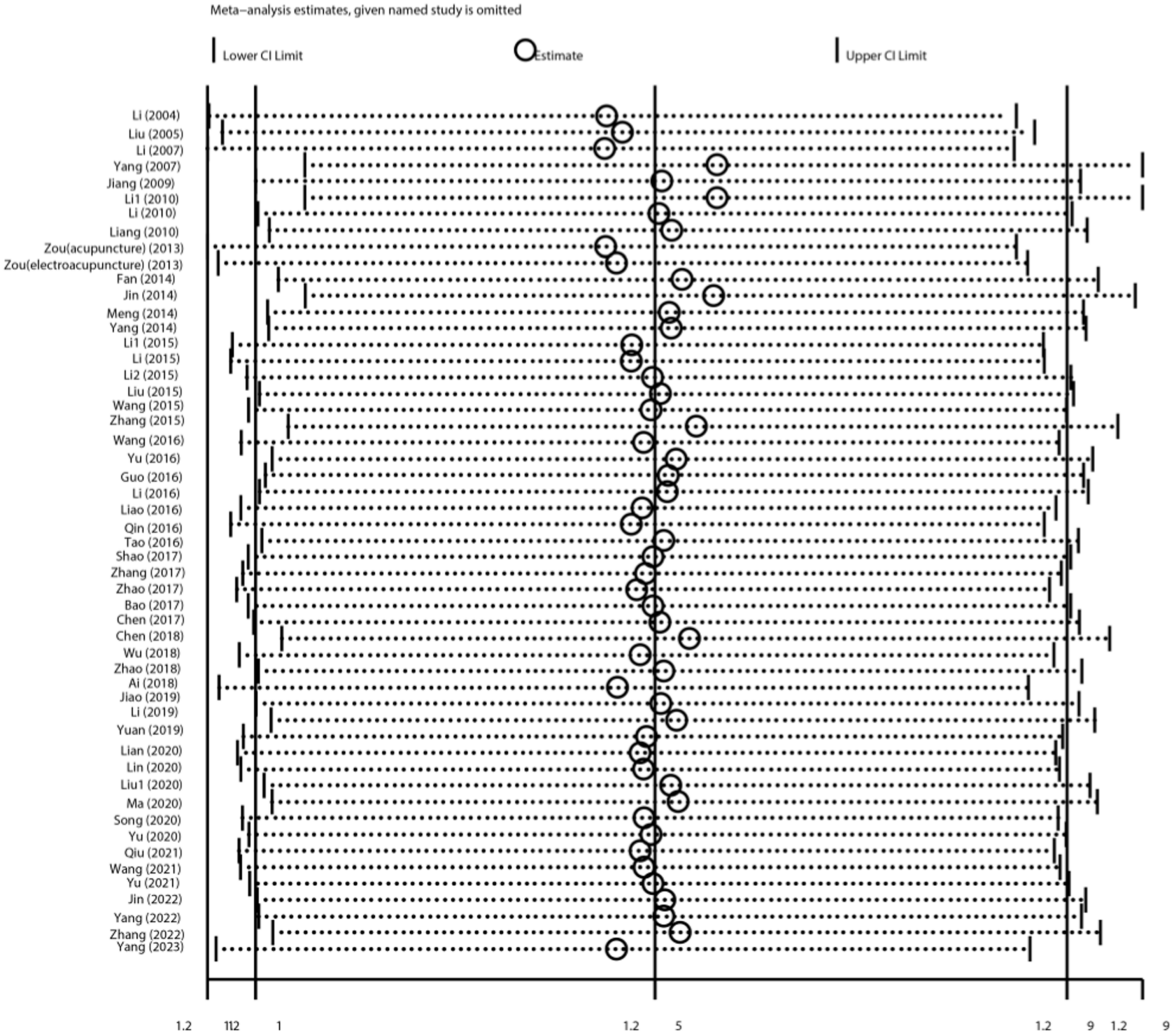
Sensitivity analysis of outcomes: the clinical effectiveness rate.

Sensitivity analysis was conducted by excluding each trial individually from the present study; the corresponding results were relatively robust (Figure 10).

**Figure 11:**
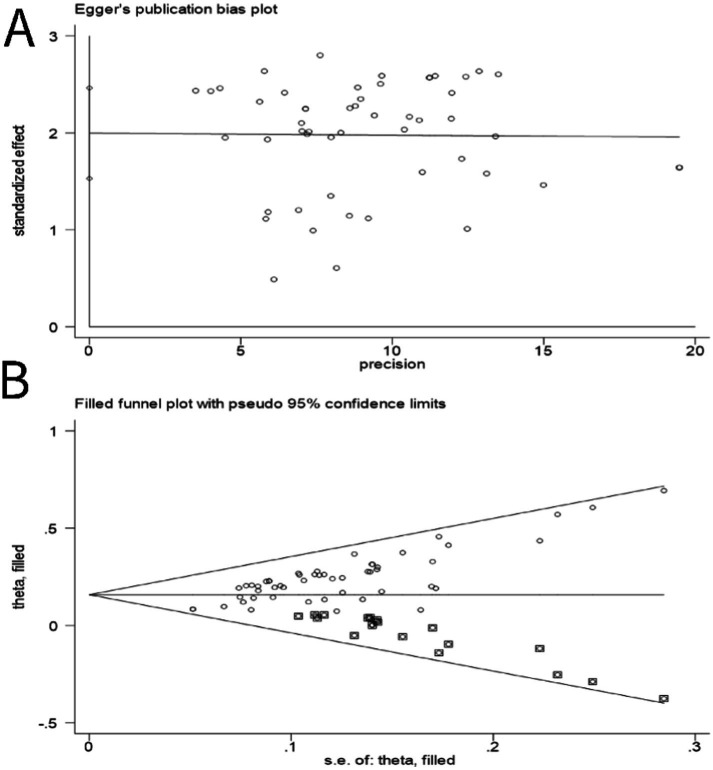
Funnel plots of outcomes: the clinical effectiveness rate. **(A)** Before correction. **(B)** After correction.

Egger’s test was employed to construct a funnel plot aimed at assessing the presence of publication bias, utilizing the metric of clinical effectiveness rate. Figure 11A illustrates that the dispersion of points within the funnel plot exhibited a perceptible asymmetry encircling the axis of symmetry, suggesting publication bias among these studies (*p* < 0.01; 95% CI: 1.53, 2.46). Using the trim and fill method for correction, after adding 19 articles (*p* < 0.01; 95% CI: 1.143, 1.207), it was consistent with the pre-correction conclusions, indicating that publication bias had no effect on the conclusion of this study; this is shown in Figure 11B.

### 4.9 Trial sequential analysis

**Figure 12:**
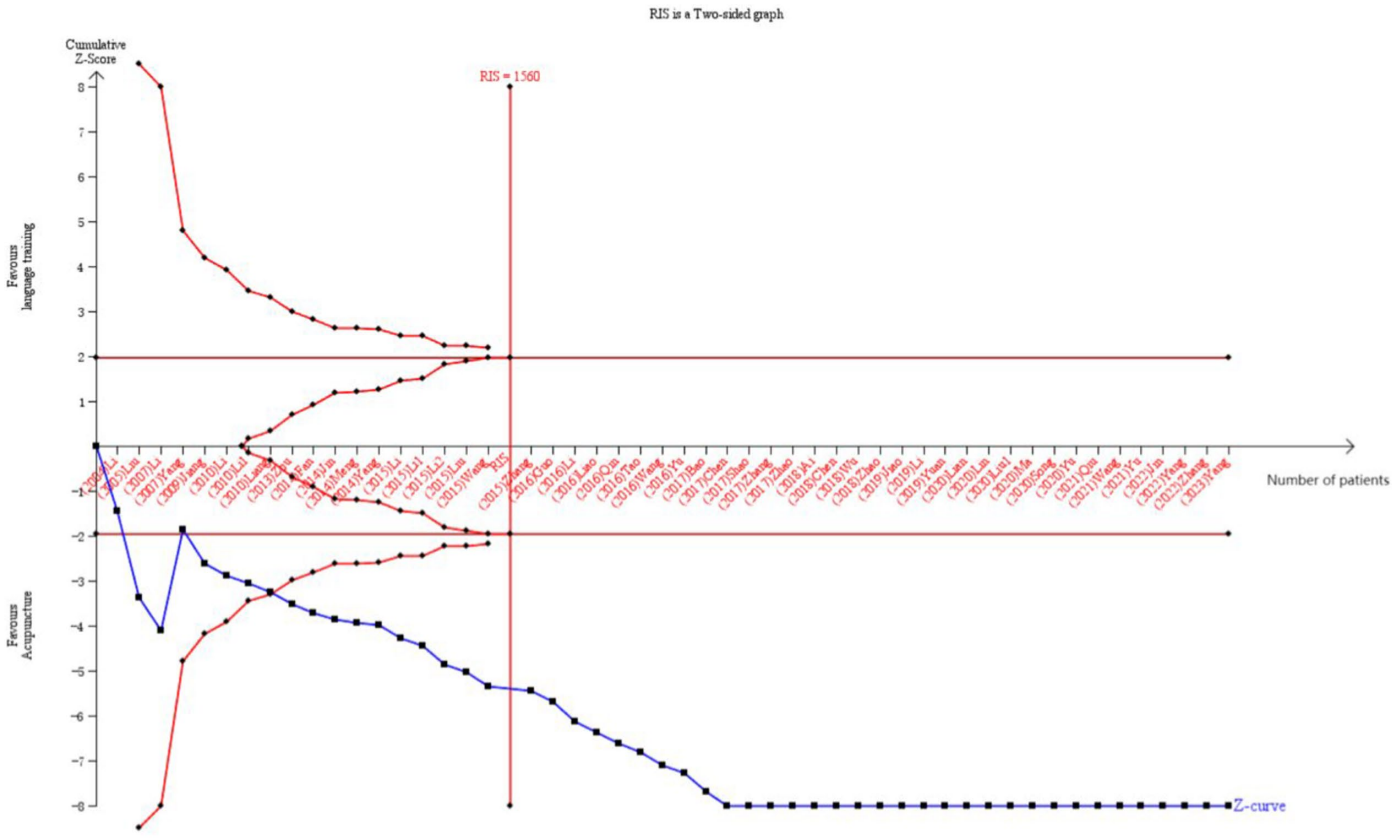
TSA on comparison of acupuncture combined with language training compared to language training alone. The crimson horizontal line represents the conventional statistical boundary of *p* = 0.05. The blue line indicates the cumulative *Z*-score of the meta-analysis. The red vertical line indicates the TSA boundary. RIS represents the required size of information.

In all, 51 RCTs (17–42, 45–53, 55–59, 61–66, 68–72) provided data on the overall clinical effectiveness rate, which was subjected to sequential analysis. This analysis was conducted with a type I error of 5% and a statistical power of 80%. The cumulative sample size was designated as the information axis, with the sample size also serving as the required information value (RIS). As depicted in Figure 12, the *Z*-curve crosses both the conventional and the TSA boundary value, underscoring the meta-analysis’s statistical significance. Additionally, the *Z*-curve also crosses the RIS boundary value, indicating that the sample size has reached the expected amount and no further RCT validation will be needed in the future.

## 5 Discussion

The results of this meta-analysis show that acupuncture combined with language training has a more favourable clinical efficiency compared with treatment using language training alone. From the GESELL development scale, acupuncture combined with language training was seen to be effective in improving the adaptive, verbal, fine motor, and personal social behaviours of aphasic children with CP, compared to treatment with language training alone. However, there is no significant difference between the effect of acupuncture combined with language training and that of language training alone, in terms of improving gross sports behaviour. In addition, compared with language training alone, acupuncture combined with language training can significantly improve the assessment of dysarthria, oral motor function, the expressive language development quotient, and the language comprehension development quotient.

Through a subgroup analysis of different types of acupuncture, it was found that, compared with language training alone, acupuncture, electroacupuncture, scalp acupuncture, and auricular point seed-pressing combined with language training can effectively improve the clinical efficacy of aphasia treatment in children with CP. However, there is no significant difference between laser acupuncture combined with language training and language training alone. The duration subgroup analysis found that acupuncture combined with language training significantly improved clinical efficiency for a treatment duration of both ≤3 months and >3 months.

Studies have shown that acupuncture in the field of neurorehabilitation can promote the recovery of somatosensory or motor function, repair nerve damage, and promote the repair of speech function (73, 74). Aphasia, a common neurological disorder, is caused by damage to the functional language areas of the brain and its associated language networks (73). Acupuncture can enhance blood circulation at the site of brain lesions and in the language regions of individuals with aphasia, promote the establishment of collateral circulation, activate the language centers, and revive neural conduction pathways (75). Evidence from multiple studies indicates that the integration of various acupuncture modalities with language training can markedly enhance the speech expression and comprehension in children with CP, while also effectively mitigating dysarthria (62, 67, 70). The main types of acupuncture used in the RCTs included in this study were body acupuncture, scalp acupuncture, and electroacupuncture. The main mechanisms of body acupuncture are stimulating the peripheral nerves, improving local blood circulation, accelerating the local activities of the cerebral cortex, strengthening the protection of the cranial nerve, attenuating the negative effects on local nerves caused by the release of lipid peroxidation, and promoting the functional recovery of the cranial nerve (76). Acupuncture can also extend the brain nerve survival cycle and improve the release of the nerve growth factor, by improving the adverse symptoms of CP (77). Modern studies show that scalp acupuncture can effectively activate the cerebral cortex function, inhibit nerve cell apoptosis, promote nerve regeneration and the growth of endogenous neural stem cells, regulate brain cell energy metabolism, and increase the oxygen carrying capacity of brain cells (78–80). In addition, scalp acupuncture has a certain awakening effect on brain cells in the dormant state and can repair the damaged neuronal cells, improving language disorders and intelligence levels (81, 82). Electroacupuncture can stimulate the proliferation and differentiation of endogenous neural stem cells in the hippocampus, preventing their excessive differentiation into astrocytes and helping to accelerate the differentiation of e NSCs into neurons, thus playing a positive role in nerve regeneration (83). It has been found that electroacupuncture can increase the expression of p53 deacetylated Bcl-2, reduce the levels of Bax and Caspase-3, cut off the endogenous apoptotic pathway, and play an anti-apoptotic effect; it can also downregulate CHOP and Caspase-12 mRNA expression to intervene in endoplasmic reticulum stress (ERS), thus reducing cell apoptosis and providing positive effects to the brain (84–86). Consequently, advocating for the broader clinical utilization of acupuncture as a therapeutic intervention for aphasia in children with CP is deemed valuable.

In our study, only one study reported adverse events (25), primarily local bleeding, subcutaneous petechiae, and crying. From this, it can be seen that the clinical effect of acupuncture combined with language training in the treatment of aphasic children with CP is remarkable and safe.

Nevertheless, our study has certain limitations. Firstly, the scope of our research is confined to single-center RCTs originating from China, where acupuncture is predominantly utilized, thereby leading to geographical constraints; Secondly, the reliability of our study was limited by the sample size, especially concerning laser acupuncture and auricular point seed-pressing, with only one RCT was included in this study; Thirdly, the specific nature of acupuncture therapy makes it challenging to achieve participant and personnel blinding, predisposing the trials to a heightened risk of bias; Additionally, the literature included in our study is exclusively in Chinese due to the regions and populations where acupuncture is commonly practiced, which may have influenced the overall quality of the studies. Despite these limitations, our meta-analysis offers an exhaustive assessment of the clinical application of acupuncture in conjunction with language training for treating aphasia in children with CP.

## 6 Conclusion

Acupuncture demonstrates efficacy when used as an adjunctive treatment for aphasia in children with CP, not only improving the patient’s adaptive, verbal, fine motor, and personal social behaviours, but also the patient’s assessment of dysarthria, oral motor function, expressive language development quotient, and language comprehension development quotient, with significant clinical efficacy. Our findings may provide valuable guidance for the use of acupuncture in clinical applications.

## Data Availability

The original contributions presented in the study are included in the article/Supplementary material, further inquiries can be directed to the corresponding authors.
