# Supplementary material for: Acupuncture combined with language training for aphasia in children with cerebral palsy: a systematic review with meta-analysis and trial sequential analysis
Source: Front Neurol. 2025 Mar 12;16:1502023. doi: 10.3389/fneur.2025.1502023 (PMC11936805; doi:10.3389/fneur.2025.1502023)
Supplement: Supplementary file 1 [file Supplementary_file_1.docx]

1. **Pubmed**

| Search number | Query |
| --- | --- |
| 1 | "Acupuncture"[Mesh] |
| 2 | (Acupuncture[Title/Abstract]) OR (Pharmacopuncture[Title/Abstract]) |
| 3 | ("Acupuncture"[Mesh]) OR ((Acupuncture[Title/Abstract]) OR (Pharmacopuncture[Title/Abstract])) |
| 4 | "Electroacupuncture"[Mesh] |
| 5 | (("Acupuncture"[Mesh]) OR ((Acupuncture[Title/Abstract]) OR (Pharmacopuncture[Title/Abstract]))) OR ("Electroacupuncture"[Mesh]) |
| 6 | ((((warm needle acupuncture[Title/Abstract]) OR (moxibustion[Title/Abstract])) OR (catgut implantation at acupoint[Title/Abstract])) OR (Auricular pressure beans[Title/Abstract])) OR (acupoint injection[Title/Abstract]) |
| 7 | ((("Acupuncture"[Mesh]) OR ((Acupuncture[Title/Abstract]) OR (Pharmacopuncture[Title/Abstract]))) OR ("Electroacupuncture"[Mesh])) OR (((((warm needle acupuncture[Title/Abstract]) OR (moxibustion[Title/Abstract])) OR (catgut implantation at acupoint[Title/Abstract])) OR (Auricular pressure beans[Title/Abstract])) OR (acupoint injection[Title/Abstract])) |
| 8 | "Cerebral Palsy"[Mesh] |
| 9 | (((((((((cerebral pals*[Title/Abstract]) OR (brain pals*[Title/Abstract])) OR (central pals*[Title/Abstract])) OR (central paralysis[Title/Abstract])) OR (cerebral paralysis[Title/Abstract])) OR (cerebral paresis[Title/Abstract])) OR (diplegia spastica[Title/Abstract])) OR (encephalopathia infantilis[Title/Abstract])) OR (Little* Disease[Title/Abstract])) OR (Spastic Diplegia*[Title/Abstract]) |
| 10 | ("Cerebral Palsy"[Mesh]) OR ((((((((((cerebral pals*[Title/Abstract]) OR (brain pals*[Title/Abstract])) OR (central pals*[Title/Abstract])) OR (central paralysis[Title/Abstract])) OR (cerebral paralysis[Title/Abstract])) OR (cerebral paresis[Title/Abstract])) OR (diplegia spastica[Title/Abstract])) OR (encephalopathia infantilis[Title/Abstract])) OR (Little* Disease[Title/Abstract])) OR (Spastic Diplegia*[Title/Abstract])) |
| 11 | "Aphasia"[Mesh] |
| 12 | (((((((((Aphasia[Title/Abstract]) OR (Alogia*[Title/Abstract])) OR (Logasthenia*[Title/Abstract])) OR (Logagnosia*[Title/Abstract])) OR (Logamnesia*[Title/Abstract])) OR (Word Deafness[Title/Abstract])) OR (Anepia*[Title/Abstract])) OR (Dejerine-Lichtheim Phenomenon[Title/Abstract])) OR (Lichtheim* Sign[Title/Abstract])) OR (Dysphasia[Title/Abstract]) |
| 13 | ("Aphasia"[Mesh]) OR ((((((((((Aphasia[Title/Abstract]) OR (Alogia*[Title/Abstract])) OR (Logasthenia*[Title/Abstract])) OR (Logagnosia*[Title/Abstract])) OR (Logamnesia*[Title/Abstract])) OR (Word Deafness[Title/Abstract])) OR (Anepia*[Title/Abstract])) OR (Dejerine-Lichtheim Phenomenon[Title/Abstract])) OR (Lichtheim* Sign[Title/Abstract])) OR (Dysphasia[Title/Abstract])) |
| 14 | "Speech Disorders"[Mesh] |
| 15 | (((((((((((((((Speech Disorder*[Title/Abstract]) OR (Cluttering*[Title/Abstract])) OR (Verbal Fluency Disorder*[Title/Abstract])) OR (Dyslalia*[Title/Abstract])) OR (Rhinolalia*[Title/Abstract])) OR (Aprosodia*[Title/Abstract])) OR (Aprosodic Speech[Title/Abstract])) OR (Dysglossia*[Title/Abstract])) OR (logopathy[Title/Abstract])) OR (speech defect[Title/Abstract])) OR (speech deficiency[Title/Abstract])) OR (speech disturbance[Title/Abstract])) OR (speech impairment[Title/Abstract])) OR (speech impediment[Title/Abstract])) OR (speech problem[Title/Abstract])) OR (speech-language pathology[Title/Abstract]) |
| 16 | ("Speech Disorders"[Mesh]) OR ((((((((((((((((Speech Disorder*[Title/Abstract]) OR (Cluttering*[Title/Abstract])) OR (Verbal Fluency Disorder*[Title/Abstract])) OR (Dyslalia*[Title/Abstract])) OR (Rhinolalia*[Title/Abstract])) OR (Aprosodia*[Title/Abstract])) OR (Aprosodic Speech[Title/Abstract])) OR (Dysglossia*[Title/Abstract])) OR (logopathy[Title/Abstract])) OR (speech defect[Title/Abstract])) OR (speech deficiency[Title/Abstract])) OR (speech disturbance[Title/Abstract])) OR (speech impairment[Title/Abstract])) OR (speech impediment[Title/Abstract])) OR (speech problem[Title/Abstract])) OR (speech-language pathology[Title/Abstract])) |
| 17 | "Dysarthria"[Mesh] |
| 18 | (((((Dysarthria*[Title/Abstract]) OR (Dysarthos*[Title/Abstract])) OR (Scanning Speech*[Title/Abstract])) OR (disarthric speech[Title/Abstract])) OR (dysarthric speech[Title/Abstract])) OR (Dysarthry[Title/Abstract]) |
| 19 | ("Dysarthria"[Mesh]) OR ((((((Dysarthria*[Title/Abstract]) OR (Dysarthos*[Title/Abstract])) OR (Scanning Speech*[Title/Abstract])) OR (disarthric speech[Title/Abstract])) OR (dysarthric speech[Title/Abstract])) OR (Dysarthry[Title/Abstract])) |
| 20 | "Language Development Disorders"[Mesh] |
| 21 | (((((Language Development Disorder*[Title/Abstract]) OR (Developmental Language Disorder*[Title/Abstract])) OR (Speech Delay*[Title/Abstract])) OR (Semantic-Pragmatic Disorder*[Title/Abstract])) OR (Central Auditory Processing Disorder[Title/Abstract])) OR (Language Delay*[Title/Abstract]) |
| 22 | ("Language Development Disorders"[Mesh]) OR ((((((Language Development Disorder*[Title/Abstract]) OR (Developmental Language Disorder*[Title/Abstract])) OR (Speech Delay*[Title/Abstract])) OR (Semantic-Pragmatic Disorder*[Title/Abstract])) OR (Central Auditory Processing Disorder[Title/Abstract])) OR (Language Delay*[Title/Abstract])) |
| 23 | (((("Aphasia"[Mesh]) OR ((((((((((Aphasia[Title/Abstract]) OR (Alogia*[Title/Abstract])) OR (Logasthenia*[Title/Abstract])) OR (Logagnosia*[Title/Abstract])) OR (Logamnesia*[Title/Abstract])) OR (Word Deafness[Title/Abstract])) OR (Anepia*[Title/Abstract])) OR (Dejerine-Lichtheim Phenomenon[Title/Abstract])) OR (Lichtheim* Sign[Title/Abstract])) OR (Dysphasia[Title/Abstract]))) OR (("Speech Disorders"[Mesh]) OR ((((((((((((((((Speech Disorder*[Title/Abstract]) OR (Cluttering*[Title/Abstract])) OR (Verbal Fluency Disorder*[Title/Abstract])) OR (Dyslalia*[Title/Abstract])) OR (Rhinolalia*[Title/Abstract])) OR (Aprosodia*[Title/Abstract])) OR (Aprosodic Speech[Title/Abstract])) OR (Dysglossia*[Title/Abstract])) OR (logopathy[Title/Abstract])) OR (speech defect[Title/Abstract])) OR (speech deficiency[Title/Abstract])) OR (speech disturbance[Title/Abstract])) OR (speech impairment[Title/Abstract])) OR (speech impediment[Title/Abstract])) OR (speech problem[Title/Abstract])) OR (speech-language pathology[Title/Abstract])))) OR (("Dysarthria"[Mesh]) OR ((((((Dysarthria*[Title/Abstract]) OR (Dysarthos*[Title/Abstract])) OR (Scanning Speech*[Title/Abstract])) OR (disarthric speech[Title/Abstract])) OR (dysarthric speech[Title/Abstract])) OR (Dysarthry[Title/Abstract])))) OR (("Language Development Disorders"[Mesh]) OR ((((((Language Development Disorder*[Title/Abstract]) OR (Developmental Language Disorder*[Title/Abstract])) OR (Speech Delay*[Title/Abstract])) OR (Semantic-Pragmatic Disorder*[Title/Abstract])) OR (Central Auditory Processing Disorder[Title/Abstract])) OR (Language Delay*[Title/Abstract]))) |
| 24 | ((Random*[Title/Abstract]) OR (RCT[Title/Abstract])) OR (Randomized controlled trial[Title/Abstract]) |
| 25 | (((((("Acupuncture"[Mesh]) OR ((Acupuncture[Title/Abstract]) OR (Pharmacopuncture[Title/Abstract]))) OR ("Electroacupuncture"[Mesh])) OR (((((warm needle acupuncture[Title/Abstract]) OR (moxibustion[Title/Abstract])) OR (catgut implantation at acupoint[Title/Abstract])) OR (Auricular pressure beans[Title/Abstract])) OR (acupoint injection[Title/Abstract]))) AND (("Cerebral Palsy"[Mesh]) OR ((((((((((cerebral pals*[Title/Abstract]) OR (brain pals*[Title/Abstract])) OR (central pals*[Title/Abstract])) OR (central paralysis[Title/Abstract])) OR (cerebral paralysis[Title/Abstract])) OR (cerebral paresis[Title/Abstract])) OR (diplegia spastica[Title/Abstract])) OR (encephalopathia infantilis[Title/Abstract])) OR (Little* Disease[Title/Abstract])) OR (Spastic Diplegia*[Title/Abstract])))) AND ((((("Aphasia"[Mesh]) OR ((((((((((Aphasia[Title/Abstract]) OR (Alogia*[Title/Abstract])) OR (Logasthenia*[Title/Abstract])) OR (Logagnosia*[Title/Abstract])) OR (Logamnesia*[Title/Abstract])) OR (Word Deafness[Title/Abstract])) OR (Anepia*[Title/Abstract])) OR (Dejerine-Lichtheim Phenomenon[Title/Abstract])) OR (Lichtheim* Sign[Title/Abstract])) OR (Dysphasia[Title/Abstract]))) OR (("Speech Disorders"[Mesh]) OR ((((((((((((((((Speech Disorder*[Title/Abstract]) OR (Cluttering*[Title/Abstract])) OR (Verbal Fluency Disorder*[Title/Abstract])) OR (Dyslalia*[Title/Abstract])) OR (Rhinolalia*[Title/Abstract])) OR (Aprosodia*[Title/Abstract])) OR (Aprosodic Speech[Title/Abstract])) OR (Dysglossia*[Title/Abstract])) OR (logopathy[Title/Abstract])) OR (speech defect[Title/Abstract])) OR (speech deficiency[Title/Abstract])) OR (speech disturbance[Title/Abstract])) OR (speech impairment[Title/Abstract])) OR (speech impediment[Title/Abstract])) OR (speech problem[Title/Abstract])) OR (speech-language pathology[Title/Abstract])))) OR (("Dysarthria"[Mesh]) OR ((((((Dysarthria*[Title/Abstract]) OR (Dysarthos*[Title/Abstract])) OR (Scanning Speech*[Title/Abstract])) OR (disarthric speech[Title/Abstract])) OR (dysarthric speech[Title/Abstract])) OR (Dysarthry[Title/Abstract])))) OR (("Language Development Disorders"[Mesh]) OR ((((((Language Development Disorder*[Title/Abstract]) OR (Developmental Language Disorder*[Title/Abstract])) OR (Speech Delay*[Title/Abstract])) OR (Semantic-Pragmatic Disorder*[Title/Abstract])) OR (Central Auditory Processing Disorder[Title/Abstract])) OR (Language Delay*[Title/Abstract]))))) AND (((Random*[Title/Abstract]) OR (RCT[Title/Abstract])) OR (Randomized controlled trial[Title/Abstract])) |

1. **Cochrane**

| ID | Search |
| --- | --- |
| #1 | MeSH descriptor: [Acupuncture] explode all trees |
| #2 | MeSH descriptor: [Electroacupuncture] explode all trees |
| #3 | (Acupuncture or Pharmacopuncture or Electroacupuncture or warm needle acupuncture or moxibustion or catgut implantation at acupoint or Auricular pressure beans or acupoint injection):ti,ab,kw |
| #4 | #1 or #2 or #3 |
| #5 | MeSH descriptor: [Cerebral Palsy] explode all trees |
| #6 | (cerebral pals* or brain pals* or central pals* or central paralysis or cerebral paralysis or cerebral paresis or diplegia spastica or encephalopathia infantilis or Little* Disease or Spastic Diplegia*):ti,ab,kw |
| #7 | #5 or #6 |
| #8 | MeSH descriptor: [Aphasia] explode all trees |
| #9 | MeSH descriptor: [Speech Disorders] explode all trees |
| #10 | MeSH descriptor: [Dysarthria] explode all trees |
| #11 | MeSH descriptor: [Language Development Disorders] explode all trees |
| #12 | (Aphasia or Alogia* or Logasthenia* or Logagnosia* or Logamnesia* or Word Deafness or Anepia* or Dejerine-Lichtheim Phenomenon or Lichtheim* Sign or Dysphasia or Speech Disorder* or Cluttering* or Verbal Fluency Disorder* or Dyslalia* or Rhinolalia* or Aprosodia* or Aprosodic Speech or Dysglossia* or logopathy or speech defect or speech deficiency or speech disturbance or speech impairment or speech impediment or speech problem or speech-language pathology or Dysarthria* or Dysarthos* or Scanning Speech* or disarthric speech or dysarthric speech or Dysarthry or Language Development Disorder* or Developmental Language Disorder* or Speech Delay* or Semantic-Pragmatic Disorder* or Central Auditory Processing Disorder or Language Delay*):ti,ab,kw |
| #13 | #8 or #9 or #10 or #11 or #12 |
| #14 | (Random* or RCT or Randomized controlled trial):ti,ab,kw |
| #15 | #4 and #7 and #13 and #14 |

1. **Embase**

| No. | Query |
| --- | --- |
| #1 | 'acupuncture'/exp |
| #2 | acupuncture:ab,ti OR pharmacopuncture:ab,ti |
| #3 | #1 OR #2 |
| #4 | 'electroacupuncture'/exp |
| #5 | #3 OR #4 |
| #6 | 'warm needle acupuncture':ab,ti OR moxibustion:ab,ti OR 'catgut implantation at acupoint':ab,ti OR 'auricular pressure beans':ab,ti OR 'acupoint injection':ab,ti |
| #7 | #5 OR #6 |
| #8 | 'cerebral palsy'/exp |
| #9 | 'cerebral pals*':ab,ti OR 'brain pals*':ab,ti OR 'central pals*':ab,ti OR 'central paralysis':ab,ti OR 'cerebral paralysis':ab,ti OR 'cerebral paresis':ab,ti OR 'diplegia spastica':ab,ti OR 'encephalopathia infantilis':ab,ti OR 'little* disease':ab,ti OR 'spastic diplegia*':ab,ti |
| #10 | #8 OR #9 |
| #11 | 'aphasia'/exp |
| #12 | aphasia:ab,ti OR alogia*:ab,ti OR logasthenia*:ab,ti OR logagnosia*:ab,ti OR logamnesia*:ab,ti OR 'word deafness':ab,ti OR anepia*:ab,ti OR 'dejerine-lichtheim phenomenon':ab,ti OR 'lichtheim* sign':ab,ti OR dysphasia:ab,ti |
| #13 | #11 OR #12 |
| #14 | 'speech disorder'/exp |
| #15 | 'speech disorder*':ab,ti OR cluttering*:ab,ti OR 'verbal fluency disorder*':ab,ti OR dyslalia*:ab,ti OR rhinolalia*:ab,ti OR aprosodia*:ab,ti OR 'aprosodic speech':ab,ti OR dysglossia*:ab,ti OR logopathy:ab,ti OR 'speech defect':ab,ti OR 'speech deficiency':ab,ti OR 'speech disturbance':ab,ti OR 'speech impairment':ab,ti OR 'speech impediment':ab,ti OR 'speech problem':ab,ti OR 'speech-language pathology':ab,ti |
| #16 | #14 OR #15 |
| #17 | 'dysarthria'/exp |
| #18 | dysarthria*:ab,ti OR dysarthos*:ab,ti OR 'scanning speech*':ab,ti OR 'disarthric speech':ab,ti OR 'dysarthric speech':ab,ti OR dysarthry:ab,ti |
| #19 | #17 OR #18 |
| #20 | 'developmental language disorder'/exp |
| #21 | 'language development disorder*':ab,ti OR 'developmental language disorder*':ab,ti OR 'speech delay*':ab,ti OR 'semantic-pragmatic disorder*':ab,ti OR 'central auditory processing disorder':ab,ti OR 'language delay*':ab,ti |
| #22 | #20 OR #21 |
| #23 | #13 OR #16 OR #19 OR #22 |
| #24 | random*:ab,ti OR rct:ab,ti OR 'randomized controlled trial':ab,ti |
| #25 | #7 AND #10 AND #23 AND #24 |

1. **Web of Science**

| # | Search Query |
| --- | --- |
| 1 | Acupuncture (Topic) OR Pharmacopuncture (Topic) OR Electroacupuncture (Topic) OR warm needle acupuncture (Topic) OR moxibustion (Topic) OR catgut implantation at acupoint (Topic) OR Auricular pressure beans (Topic) OR acupoint injection (Topic) and Preprint Citation Index (Exclude – Database) |
| 2 | cerebral pals* (Topic) OR brain pals* (Topic) OR central pals* (Topic) OR central paralysis (Topic) OR cerebral paralysis (Topic) OR cerebral paresis (Topic) OR diplegia spastica (Topic) OR encephalopathia infantilis (Topic) OR Little* Disease (Topic) OR Spastic Diplegia* (Topic) and Preprint Citation Index (Exclude – Database) |
| 3 | Aphasia (Topic) OR Alogia* (Topic) OR Logasthenia* (Topic) OR Logagnosia* (Topic) OR Logamnesia* (Topic) OR Word Deafness (Topic) OR Anepia* (Topic) OR Dejerine-Lichtheim Phenomenon (Topic) OR Lichtheim* Sign (Topic) OR Dysphasia (Topic) OR Speech Disorder* (Topic) OR Cluttering* (Topic) OR Verbal Fluency Disorder* (Topic) OR Dyslalia* (Topic) OR Rhinolalia* (Topic) OR Aprosodia* (Topic) OR Aprosodic Speech (Topic) OR Dysglossia* (Topic) OR logopathy (Topic) OR speech defect (Topic) OR speech deficiency (Topic) OR speech disturbance (Topic) OR speech impairment (Topic) OR speech impediment (Topic) OR speech problem (Topic) OR speech-language pathology (Topic) OR Dysarthria* (Topic) OR Dysarthos* (Topic) OR Scanning Speech* (Topic) OR disarthric speech (Topic) OR dysarthric speech (Topic) OR Dysarthry (Topic) OR Language Development Disorder* (Topic) OR Developmental Language Disorder* (Topic) OR Speech Delay* (Topic) OR Semantic-Pragmatic Disorder* (Topic) OR Central Auditory Processing Disorder (Topic) OR Language Delay* (Topic) and Preprint Citation Index (Exclude – Database) |
| 4 | Random* (Topic) OR RCT (Topic) OR Randomized controlled trial (Topic) and Preprint Citation Index (Exclude – Database) |
| 5 | #4 AND #3 AND #2 AND #1 and Preprint Citation Index (Exclude – Database) |
