# Supplementary material for: Acupuncture combined with language training for aphasia in children with cerebral palsy: a systematic review with meta-analysis and trial sequential analysis
Source: Front Neurol. 2025 Mar 12;16:1502023. doi: 10.3389/fneur.2025.1502023 (PMC11936805; doi:10.3389/fneur.2025.1502023)
Supplement: Supplementary file 2 [file Supplementary_file_2.docx]

1. **McIntyre S, Goldsmith S, Webb A, Ehlinger V, Hollung SJ, McConnell K, et al. Global prevalence of cerebral palsy: A systematic analysis. Dev Med Child Neurol 2022 Dec;64(12):1494-1506.**

**Title:** Global prevalence of cerebral palsy: A systematic analysis

**Authors:** McIntyre S, Goldsmith S, Webb A, Ehlinger V, Hollung SJ, McConnell K, Arnaud C, Smithers-Sheedy H, Oskoui M, Khandaker G, Himmelmann K; Global CP Prevalence Group*

**Abstract:** Aim: To determine trends and current estimates in regional and global prevalence

of cerebral palsy (CP). Method: A systematic analysis of data from participating CP registers/surveillance systems and population- based prevalence studies (from birth year 1995) was performed. Quality and risk of bias were assessed for both data sources. Analyses were

conducted for pre-/perinatal, postnatal, neonatal, and overall CP. For each region, trends were statistically classified as increasing, decreasing, heterogeneous, or no change, and most recent prevalence estimates with 95% confidence intervals (CI) were calculated. Meta-analyses were conducted to determine current birth prevalence estimates (from birth year 2010). Results: Forty-one regions from 27 countries across five continents were represented. Pre-/perinatal birth prevalence declined significantly across Europe and Australia (11 out of 14 regions), with no change in postneonatal CP. From the limited but increasing data available from regions in low-and middle-income countries (LMICs), birth prevalence for pre-/perinatal CP was as high as 3.4 per 1000 (95% CI 3.0-3.9) live births. Following meta- analyses, birth prevalence for pre-/perinatal CP in regions from high- income countries (HICs) was 1.5 per 1000 (95% CI 1.4-1.6) live births, and 1.6 per 1000 (95% CI 1.5-1.7) live births when postneonatal CP was included. Interpretation: The birth prevalence estimate of CP in HICs declined to 1.6 per 1000 live births. Data available from LMICs indicated markedly higher birth prevalence.

1. **Li XJ, Qiu HB, Jiang ZM, Pang W, Guo J, Zhu LJ, et al. Epidemiological characteristics of cerebral palsy in twelve province in China. Chinese Journal of Applied Clinical Pediatrics 2018;33(5):378-383.**

**Title:** Epidemiological characteristics of cerebral palsy in twelve province in China

**Authors:** Li XJ, Qiu HB, Jiang ZM, Pang W, Guo J, Zhu LJ, Lv ZH, Wang LP, Sun QF, Yao SP, Sun Y, Guo LM, Li XZ

**Abstract:** Objective: To investigate the epidemiological characteristics of cerebral palsy(CP) in children aged 1-6 years in China, including the incidence,prevalence, type of CP, etiology, prevention and rehabilitation status. Methods: The survey was carried out by standard questionnaires, multi-center collaboration, stratified-cluster ran-dom sampling method. The surveyed adopted the following principles: streets in the city and villages in the rural areas, and the number of the urban and rural children was the same, and the proportion of children in each age group was balanced. The investigation areas included provinces and autonomous regions, including Heilongjiang, Beijing, Henan, Shandong, Shanxi, Shaanxi, Anhui, Hunan, Guangxi, Guangdong, Chongqing and Qinghai, and 323858 children were in-vestigated. Results: The incidence of CP was 2.48‰(155/62591 cases), and the prevalence was 2.46‰(797/323858 cases)(1-6 years old). The prevalence varied in different regions, in which the highest prevalence was 5.40‰(54/9998 cases) in Qinghai province, and the lowest prevalence was 1.04‰(47/45133 cases)in Shandong province. The prevalence of the males(2.64‰,461/174391 cases)was higher than that of the females(2.25‰, 336/149467 cases), and the difference was statistically significant(*P*<0.05). The types of CP were spastic type(58.85%, 469/797 cases), mixed type(13.17%,105/797 cases), dyskinetic(9.79%,78/797 cases), hypotonic(8.28%,66/797 cases), ataxia(6.25%,52/797 cases)and rigid(3.39%,27/797 cases) respectively in 797 CP children. The first three risk factors for CP were long-term exposure to harmful physical factors during pregnancy, whether there were birth defects among the three generations of relatives of the children,such as children's peers, parents or grandparents, whether there were birth defects among the children's peers, parents or grandparents,and neonatal jaundice or persistent jaundice. Among 797 CP children, 79.67% of the children with CP were timely detected and treated in the local hospitals,while the other 19.93% of them were not timely treated. The places which could give them timely detection and early diagnosis and treatment were general hospitals(42.97%), Maternity and Infant Hospitals (27.03%)and Children's Hospitals(20.31%). The main rehabilitation methods for 797 children with CP were 34.58% in the hospitals or rehabilitation centers, 31.61% in the communities(including at home), 33.80% mainly in the medical institution,and in the communities they could also receive partially rehabilitation services. Conclusions: The prevalence of CP in China is coincident with international levels. The prevalence rate of CP in males is higher than that in females. The types of CP distribution are accorded with international distribution characteristics. There were still some children with CP who could not receive timely detection and treatment.Rehabilitation at the medical institutions is the chief way and proper rehabilitation guidance should be carried out in the communities.

1. **Mou Z, Teng W, Ouyang H, Chen Y, Liu Y, Jiang C, et al. Quantitative analysis of vowel production in cerebral palsy children with dysarthria. J Clin Neurosci. 2019 Aug;66:77-82.**

**Title:** Quantitative analysis of vowel production in cerebral palsy children with dysarthria

**Authors:** Mou Z, Teng W, Ouyang H, Chen Y, Liu Y, Jiang C, Zhang J, Chen Z

**Abstract:** Objective: The present study aimed to identify certain acoustic parameters for speech evaluation in cerebral palsy children with dysarthria. Methods: The subject included 30 native Mandarin-Speaking children with cerebral palsy, who were 5-15 years old, and 13 healthy children in a similar age range. Each subject was recorded while producing a list of 12 Mandarin words, which included three syllables ('ba', 'bi' and 'du'), in all four Mandarin tones. The formants (F1 and F2) of monophthong vowels /a, i, u/ were extracted from each vowel token. Based on F1 and F2, the vowel acoustic indexes VSA, VAI and FCR were calculated and analyzed. Results: Compared with the control group, the cerebral palsy group had significantly low F1 and F2 in vowel /a/ (*P* < 0.05), and F2 in vowel /i/ (*P* < 0.05), while F1 and F2 in vowel /u/ and F1 in vowel /i/ had no significant difference. Between the healthy group and cerebral palsy group, the differences in VSA, VAI and FCR were all statistically significant. Conclusion: Children with cerebral palsy have reduced vowel space and speech articulation. The significant difference in vowel acoustic indexes (VSA, VAI and FCR) among the two groups revealed that the three indexes were sensitive to the variation of the vowels production in children with cerebral palsy, and that these may be used as an evaluation method of speech intelligibility caused by impaired vowel pronunciation in children with cerebral palsy, and the effect of rehabilitation therapy.

1. **Valadão P, Piitulainen H, Haapala EA, Parviainen T, Avela J, Finni T. Exercise intervention protocol in children and young adults with cerebral palsy: theeffects of strength, flexibility and gait training on physical performance, neuromuscular mechanisms and cardiometabolic risk factors(EXECP). BMC Sports Sci Med Rehabil 2021;13(1):17.**

**Title:** Exercise intervention protocol in children and young adults with cerebral palsy:theeffects of strength, flexibility and gait training on physical performance, neuromuscular mechanisms and cardiometabolic risk factors(EXECP)

**Authors:** Valadão P, Piitulainen H, Haapala EA, Parviainen T, Avela J, Finni T

**Abstract:** Background: Individuals with cerebral palsy (CP) have problems in everyday tasks such as walking and climbing stairs due to a combination of neuromuscular impairments such as spasticity, muscle weakness, reduced joint flexibility and poor coordination. Development of evidence-based interventions are in pivotal role in the development of better targeted rehabilitation of CP, and thus in maintaining their motor function and wellbeing. Our aim is to investigate the efficacy of an individually tailored, multifaceted exercise intervention (EXECP) in children and young adults with CP. EXECP is composed of strength, flexibility and gait training. Furthermore, this study aims to verify the short-term retention of the adaptations three months after the end of the EXECP intervention. Methods: Twenty-four children and young adults with spastic CP will be recruited to participate in a 9-month research project with a 3-month training intervention, consisting of two to three 90-min sessions per week. In each session, strength training for the lower limbs and trunk muscles, flexibility training for the lower limbs and inclined treadmill gait training will be performed. We will evaluate muscle strength, joint flexibility, neuromuscular and cardiometabolic parameters. A nonconcurrent multiple baseline design with two pre-tests and two post-tests all interspaced by three months is used. In addition to the CP participants, 24 typically developing age and sex-matched participants will perform the two pre-tests (i.e. no intervention) to provide normative data. Discussion: This study has a comprehensive approach examining longitudinal effects of wide variety of variables ranging from physical activity and gross motor function to sensorimotor functions of the brain and neuromuscular and cardiometabolic parameters, providing novel information about the adaptation mechanisms in cerebral palsy. To the best of our knowledge, this is the first intervention study providing supervised combined strength, flexibility and gait training for young individuals with CP.

1. **Xue J, Mo Y. Application of vocal organ correction combined with language training in the rehabilitation of children with cerebral palsy and language disorder. Transl Pediatr. 2020 Oct;9(5):645-652.**

**Title:** Application of vocal organ correction combined with language training in the rehabilitation of children with cerebral palsy and language disorder

**Authors:** Xue J, Mo Y

**Abstract:** Background: To explore the effect of vocal organ correction combined with language training on the rehabilitation of children with cerebral palsy (CP) and language disorder. Methods: A total of 98 children with CP and language disorder were divided into two groups (49 cases in each group) using a random number table: the control group and the test group. The control group was given language training alone, while the test group received vocal organ correction combined with language training. The changes in language function classification, efficacy, and family satisfaction before and after the treatments were compared. Results: A significant difference was identified in language function classification between the two groups before and after treatment (*P*<0.05). The language function classification of the two groups was also significantly different after treatment (*P*<0.05), as was the distribution of clinical efficacy between the two groups (*P*<0.05). The total effective rate for the test group was 91.84%, which was higher than the 73.47% for the control group (*P*<0.05). Family satisfaction between the two groups differed significantly (*P*<0.05), and the total satisfaction rate of families in the test group was 87.76%, which was higher than the 69.39% in the control group (*P*<0.05). Conclusions: Vocal organ correction combined with language training can improve the language function of children with CP and language disorder, has ideal efficacy, and can also enhance family satisfaction during rehabilitation.

1. **Wang M, Liu W, Ge J, Liu S. The immunomodulatory mechanisms for acupuncture practice. Front Immunol. 2023 Apr 6;14:1147718.**

**Title:** The immunomodulatory mechanisms for acupuncture practice

**Authors:** Wang M, Liu W, Ge J, Liu S

**Abstract:** The system physiology approaches that emerge in western countries in recent years echo the holistic view of ancient Traditional Chinese Medicine (TCM) practices that deal with the root, rather than only the symptoms of diseases. Particularly, TCM practices, including acupuncture, emphasize the mobilization of self-healing mechanisms to bring back body homeostasis. Acupuncture has been practiced for over two thousand years to modulate body physiology via stimulation at specific body regions (acupoints). With the development of various research on acupuncture therapy, its regulatory effect on the immune system has been gradually recognized, especially on immunological diseases, including infectious and allergic diseases. In this study, we reviewed the immunomodulatory mechanism of acupuncture and systematically integrates existing research to respectively elucidate the modulatory mechanisms of acupuncture on the innate immune system, adaptive immune system, and well-known neuroanatomical mechanisms, including intact somatosensory-autonomic reflex pathway. With the advances made in recent systems physiology studies, we now have a great opportunity to gain insight into how acupuncture modulates immunity, and subsequently improves its efficacy.

1. **Li F, He T, Xu Q, Lin LT, Li H, Liu Y, et al. What is the Acupoint? A preliminary review of Acupoints. Pain Med. 2015 Oct;16(10):1905-1915.**

**Title:** What is the Acupoint? A preliminary review of Acupoints

**Authors:** Li F, He T, Xu Q, Lin LT, Li H, Liu Y, Shi GX, Liu CZ

**Abstract:** Background: According to traditional Chinese medicine (TCM) theory, acupoints are specifically chosen sites of acupuncture manipulation, and also the basis for studying the mechanism of acupuncture. Stimulating different acupoints on the body surface could provide various therapeutic benefits. However, what is the acupoint? This question is not clear.

Review summary: We focuse on examining the function of acupoints from different perspectives, including the local and the systemic effects of stimulating acupoints. For example, acupoints may release certain substances or incur some changes, which could adjust the function of organs, maintain homeostasis. Furthermore, the therapeutic effects of verum acupoints versus sham acupoints were discussed. However, due to insufficience in evidence and in current methodologies, research into mechanisms of acupuncture is still incomplete.

Conclusion: This review might explain, to some extent, what an acupoint is. Further research into the identity of acupoints is warranted, and multidisciplinary methods using novel technologies may yield significant advances over existing knowledge.

1. **Zhu Y, Yang Y, Li J. Does acupuncture help patients with spasticity? A narrative review. Ann Phys Rehabil Med. 2019 Jul;62(4):297-301.**

**Title:** Does acupuncture help patients with spasticity? A narrative review

**Authors:** Zhu Y, Yang Y, Li J

**Abstract:** Spasticity is a motor disorder encountered after upper motor neuron lesions. It adversely affects quality of life in most patients and causes long-term burden of care and has significant financial implications. The effect of conventional therapies for spasticity including physical therapy, surgery, and pharmacotherapy are not always satisfying because of the short-term effects or side effects in some patients. Acupuncture is a part of traditional medicine originating from China. It has been used to resolve functional recovery problems after central nervous system injury for many years in Asian countries and is increasingly popular in western countries. Some researches suggest that acupuncture has therapeutic potential to help improve limb movement function and decrease the severity of spasticity. This review synthesizes studies involving stroke, brain injury, spinal cord injury, cerebral palsy, and multiple sclerosis to give an overall picture of the effect and potential mechanisms of acupuncture on spasticity occurring after upper motor neuron lesions. Moderate-quality evidence suggests that electroacupuncture combined with conventional routine care (pharmacological/rehabilitation) could reduce spasticity and improve motor function and activities in daily living after stroke. However, there is not enough evidence to conclude that acupuncture (including electroacupuncture) could reduce spasticity with other central nervous system diseases.

1. **Zhang Z, Chen L, Guo Y, Li D, Zhang J, Liu L, et al. The neuroprotective and neural circuit mechanisms of acupoint stimulation for cognitive impairment. Chin Med. 2023 Jan 20;18(1):8.**

**Title:** The neuroprotective and neural circuit mechanisms of acupoint stimulation for cognitive impairment

**Authors:** Zhang Z, Chen L, Guo Y, Li D, Zhang J, Liu L, Fan W, Guo T, Qin S, Zhao Y, Xu Z, Chen Z

**Abstract:** Cognitive impairment is a prevalent neurological disorder that burdens families and the healthcare system. Current conventional therapies for cognitive impairment, such as cholinesterase inhibitors and N-methyl-d-aspartate receptor antagonists, are unable to completely stop or reverse the progression of the disease. Also, these medicines may cause serious problems with the digestive system, cardiovascular system, and sleep. Clinically, stimulation of acupoints has the potential to ameliorate the common symptoms of a variety of cognitive disorders, such as memory deficit, language dysfunction, executive dysfunction, reduced ability to live independently, etc. There are common acupoint stimulation mechanisms for treating various types of cognitive impairment, but few systematic analyses of the underlying mechanisms in this domain have been performed. This study comprehensively reviewed the basic research from the last 20 years and found that acupoint stimulation can effectively improve the spatial learning and memory of animals. The common mechanism may be that acupoint stimulation protects hippocampal neurons by preventing apoptosis and scavenging toxic proteins. Additionally, acupoint stimulation has antioxidant and anti-inflammatory effects, promoting neural regeneration, regulating synaptic plasticity, and normalizing neural circuits by restoring brain functional activity and connectivity. Acupoint stimulation also inhibits the production of amyloid β-peptide and the phosphorylation of Tau protein, suggesting that it may protect neurons by promoting correct protein folding and regulating the degradation of toxic proteins via the autophagy-lysosomal pathway. However, the benefits of acupoint stimulation still need to be further explored in more high-quality studies in the future.

1. **Lv XL, Sun ZR, Hao JS, Thomas Hodge. Overview of Treatment of Cerebral Palsy with Acupuncture in the Recent Five Years. Acta Chinese Medicine and Pharmacology. 2017;45(01):51-56.**

**Title:** Overview of Treatment of Cerebral Palsy with Acupuncture in the Recent Five Years

**Authors:** Lv XL, Sun ZR, Hao JS, Thomas Hodge

**Abstract:** In recent years, acupuncture has increasingly being integrated into pediatric health care. It was used on ~150,000 children (0.2%). We aim to update the evidence for the efficacy and safety of acupuncture for children and evaluate the methodological qualities of these studies to improve future research in this area. We included 24 systematic reviews, comprising 142 randomized controlled trials (RCTs) with 12,787 participants. Only 25% (6/24) reviews were considered to be high quality (10.00±0.63). High-quality systematic reviews and Cochrane systematic reviews tend to yield neutral or negative results (*P* = 0.052, 0.009 respectively). The efficacy of acupuncture for five diseases (Cerebral Palsy (CP), nocturnal enuresis, tic disorders, amblyopia, and pain reduction) is promising. It was unclear for hypoxic ischemic encephalopathy, attention deficit hyperactivity disorder, mumps, autism spectrum disorder (ASD), asthma, nausea/vomiting, and myopia. Acupuncture is not effective for epilepsy. Only six reviews reported adverse events (AEs) and no fatal side effects were reported. The efficacy of acupuncture for some diseases is promising and there have been no fatal side effects reported. Further high-quality studies are justified, with five diseases in particular as research priorities.

1. **Zhang XY, Fu JN, Gao LJ. Effect of acupuncture therapy for invigorating the kidney and strengthening brain in the adjuvant treatment of children with cerebral palsy and its influence on language development quotient and comprehensive function. Chinese Pediatrics of Integrated Traditional and Western Medicine. 2021;13(01):10-13.**

**Title:** Effect of acupuncture therapy for invigorating the kidney and strengthening brain in the adjuvant treatment of children with cerebral palsy and its influence on language development quotient and comprehensive function

**Authors:** Zhang XY, Fu JN, Gao LJ

**Abstract:** Objective: To explore the effect of acupuncture therapy for invigorating the kidney and strengthening brain combined with language group training in the treatment of children with cerebral palsy(CP) and its influence on language development quotient and comprehensive functions. Methods: A total of 84 children with cerebral palsy who were treated in our hospital from June 2016 to June 2019 were selected and randomly divided into a control group and an observation group with 42 cases each. Both groups were given language group training, and the observation group also received acupuncture therapy for invigorating kidney and strengthening brain. The clinical effects, language development quotient score and comprehensive function score of the two groups of children with cerebral palsy were observed. Results: The total effective rate of the observation group was 83.33%(35/42), which was significantly higher than that of the control group 54.76%(23/42), and the difference was statistically significant(*P*<0.05); after 3 months of treatment, language development quotient score, and the scores of cognitive function, motor function, self-care function and social adaptation in observation group were significantly higher than those of the control group, and the difference was statistically significant(*P*<0.05). Conclusion: Acupuncture therapy for invigorating the kidney and strengthening brain combined with language group training is effective in the treatment of children with cerebral palsy, which can increase language development quotient and improve comprehensive functions.

1. **Fu GJ, Yu Y. Clinical research on treating language disorder of children with cerebral palsy by acupuncture. Clinical Journal of Chinese Medicine. 2019;11(07):41-42.**

**Title:** Clinical research on treating language disorder of children with cerebral palsy by acupuncture

**Authors:** Fu GJ, Yu Y

**Abstract:** Objective: To study the clinical effect of acupuncture on language disorder of children with cerebral palsy. Methods: 36 children with cerebral palsy were selected as the research object, and they were divided into two groups, 18 cases in each. The control group was given conventional treatment, and the experimental group was treated by acupuncture more, and the effects in the two groups were compared. Results: The effective rate in the experimental group was significantly higher than the control group, *P*<0.05. Conclusion: Acupuncture can effectively improve the language function of the patients and help them restore the basic expression ability. It has significant therapeutic effect and is worthy of being popularized in clinic.

1. **Hutton B, Salanti G, Caldwell DM, Chaimani A, Schmid CH, Cameron C, et al. The PRISMA extension statement for reporting of systematic reviews incorporating network meta-analyses of health care interventions: checklist and explanations. Ann Intern Med 2015 Jun 2;162(11):777-784.**

**Title:** The PRISMA extension statement for reporting of systematic reviews incorporating network meta-analyses of health care interventions: checklist and explanations

**Authors:** Hutton B, Salanti G, Caldwell DM, Chaimani A, Schmid CH, Cameron C, Ioannidis JP, Straus S, Thorlund K, Jansen JP, Mulrow C, Catalá-López F, Gøtzsche PC, Dickersin K, Boutron I, Altman DG, Moher D

**Abstract:** The PRISMA statement is a reporting guideline designed to improve the completeness of reporting of systematic reviews and meta-analyses. Authors have used this guideline worldwide to prepare their reviews for publication. In the past, these reports typically compared 2 treatment alternatives. With the evolution of systematic reviews that compare multiple treatments, some of them only indirectly, authors face novel challenges for conducting and reporting their reviews. This extension of the PRISMA (Preferred Reporting Items for Systematic Reviews and Meta-analyses) statement was developed specifically to improve the reporting of systematic reviews incorporating network meta-analyses. A group of experts participated in a systematic review, Delphi survey, and face-to-face discussion and consensus meeting to establish new checklist items for this extension statement. Current PRISMA items were also clarified. A modified, 32-item PRISMA extension checklist was developed to address what the group considered to be immediately relevant to the reporting of network meta-analyses. This document presents the extension and provides examples of good reporting, as well as elaborations regarding the rationale for new checklist items and the modification of previously existing items from the PRISMA statement. It also highlights educational information related to key considerations in the practice of network meta-analysis. The target audience includes authors and readers of network meta-analyses, as well as journal editors and peer reviewers.

1. **Sterne JAC, Savović J, Page MJ, Elbers RG, Blencowe NS, Boutron I, et al. RoB 2: a revised tool for assessing risk of bias in randomised trials. BMJ 2019 Aug 28;366:l4898.**

**Title:** RoB 2: a revised tool for assessing risk of bias in randomised trials

**Authors:** Sterne JAC, Savović J, Page MJ, Elbers RG, Blencowe NS, Boutron I, Cates CJ, Cheng HY, Corbett MS, Eldridge SM, Emberson JR, Hernán MA, Hopewell S, Hróbjartsson A, Junqueira DR, Jüni P, Kirkham JJ, Lasserson T, Li T, McAleenan A, Reeves BC, Shepperd S, Shrier I, Stewart LA, Tilling K, White IR, Whiting PF, Higgins JPT

**Abstract:** Assessment of risk of bias is regarded as an essential component of a systematic review on the effects of an intervention. The most commonly used tool for randomised trials is the Cochrane risk-of-bias tool. We updated the tool to respond to developments in understanding how bias arises in randomised trials, and to address user feedback on and limitations of the original tool.

1. **Salanti G, Del Giovane C, Chaimani A, Caldwell DM, Higgins JP. Evaluating the quality of evidence from a network meta-analysis. PLoS One 2014 Jul 3;9(7):e99682.**

**Title:** Evaluating the quality of evidence from a network meta-analysis

**Authors:** Salanti G, Del Giovane C, Chaimani A, Caldwell DM, Higgins JP

**Abstract:** Systematic reviews that collate data about the relative effects of multiple interventions via network meta-analysis are highly informative for decision-making purposes. A network meta-analysis provides two types of findings for a specific outcome: the relative treatment effect for all pairwise comparisons, and a ranking of the treatments. It is important to consider the confidence with which these two types of results can enable clinicians, policy makers and patients to make informed decisions. We propose an approach to determining confidence in the output of a network meta-analysis. Our proposed approach is based on methodology developed by the Grading of Recommendations Assessment, Development and Evaluation (GRADE) Working Group for pairwise meta-analyses. The suggested framework for evaluating a network meta-analysis acknowledges (i) the key role of indirect comparisons (ii) the contributions of each piece of direct evidence to the network meta-analysis estimates of effect size; (iii) the importance of the transitivity assumption to the validity of network meta-analysis; and (iv) the possibility of disagreement between direct evidence and indirect evidence. We apply our proposed strategy to a systematic review comparing topical antibiotics without steroids for chronically discharging ears with underlying eardrum perforations. The proposed framework can be used to determine confidence in the results from a network meta-analysis. Judgements about evidence from a network meta-analysis can be different from those made about evidence from pairwise meta-analyses.

1. **Wetterslev J, Jakobsen JC, Gluud C. Trial Sequential Analysis in systematic reviews with meta-analysis. BMC Med Res Methodol 2017 Mar 6;17(1):39.**

**Title:** Trial Sequential Analysis in systematic reviews with meta-analysis

**Authors:** Wetterslev J, Jakobsen JC, Gluud C

**Abstract:** Background: Most meta-analyses in systematic reviews, including Cochrane ones, do not have sufficient statistical power to detect or refute even large intervention effects. This is why a meta-analysis ought to be regarded as an interim analysis on its way towards a required information size. The results of the meta-analyses should relate the total number of randomised participants to the estimated required meta-analytic information size accounting for statistical diversity. When the number of participants and the corresponding number of trials in a meta-analysis are insufficient, the use of the traditional 95% confidence interval or the 5% statistical significance threshold will lead to too many false positive conclusions (type I errors) and too many false negative conclusions (type II errors). Methods: We developed a methodology for interpreting meta-analysis results, using generally accepted, valid evidence on how to adjust thresholds for significance in randomised clinical trials when the required sample size has not been reached. Results: The Lan-DeMets trial sequential monitoring boundaries in Trial Sequential Analysis offer adjusted confidence intervals and restricted thresholds for statistical significance when the diversity-adjusted required information size and the corresponding number of required trials for the meta-analysis have not been reached. Trial Sequential Analysis provides a frequentistic approach to control both type I and type II errors. We define the required information size and the corresponding number of required trials in a meta-analysis and the diversity (D2) measure of heterogeneity. We explain the reasons for using Trial Sequential Analysis of meta-analysis when the actual information size fails to reach the required information size. We present examples drawn from traditional meta-analyses using unadjusted naïve 95% confidence intervals and 5% thresholds for statistical significance. Spurious conclusions in systematic reviews with traditional meta-analyses can be reduced using Trial Sequential Analysis. Several empirical studies have demonstrated that the Trial Sequential Analysis provides better control of type I errors and of type II errors than the traditional naïve meta-analysis. Conclusions: Trial Sequential Analysis represents analysis of meta-analytic data, with transparent assumptions, and better control of type I and type II errors than the traditional meta-analysis using naïve unadjusted confidence intervals.

1. **Li HM. Acupuncture plus oral function training promotes recovery of speech disorders in paediatric cerebral palsy. Chinese Journal of Tissue Engineering Research 2004;8(21):4353.**

**Title:** Acupuncture plus oral function training promotes recovery of speech disorders in paediatric cerebral palsy

**Authors:** Li HM

**Abstract:** Objective: To observe the therapeutic effect of acupuncture plus oral function training on speech disorders in children with cerebral palsy, and to test the hypothesis that the therapeutic effect of the addition of acupuncture therapy is better than that of oral function training alone. Methods: 100 cases of cerebral palsy children were randomly divided into acupuncture group, training group and combined group of 30 cases each, acupuncture group used acupuncture method, training group used modern oral function training and rehabilitation method, and combined group used acupuncture and oral function training method, and each group was treated for 60d to compare the efficacy. Results: According to the categories of basically normal, obvious effect, effective and ineffective, there were 4, 6, 13 and 7 cases in the combined group with a total effective rate of 76.6%, and there were 2, 5, 7 and 16 cases in the training group and the combined group with a total effective rate of 53.3% and 1, 3, 9 and 17 cases with a total effective rate of 56.6% respectively. The difference in the total effective rate of each group was significant (*X*^2^=5.71, 6.94, *P*＜0.05). Conclusion: Acupuncture and oral function training are more beneficial to the recovery of speech disorders in children with cerebral palsy.

1. **Liu ZH, Ma MM, Pan PG, Fu WJ, Hu JJ. TREATMENT OF CEREBRAL PALSY WITH APHASIA BY LINGUISTIC TRAINING COMBINED WITH ACUPUNCTURE. World Journal of Acupuncture-Moxibustion 2005;(04):31-33+36.**

**Title:** TREATMENT OF CEREBRAL PALSY WITH APHASIA BY LINGUISTIC TRAINING COMBINED WITHACUPUNCTURE

**Authors:** Liu ZH, Ma MM, Pan PG, Fu WJ, Hu JJ

**Abstract:** Objective: To explore the best remedies for cerebral palsy with aphasia. Methods: A total of 76 cases of cerebral palsy children with aphasia were evenly randomized into control group and treatment group. Patients of treatment group were treated with “consciousness-restoring needling”plus linguistic training and those of control group treated with simple linguistic training method. Acupuncture was given once every other dayand linguistic training conducted once 6 times a weekwith 10 times being a therapeutic course and the interval between two weeks being10-15days. Following 3 courses of treatmentthe therapeutic effect was analyzed. Results: After 3 courses of treatmentof the two 38 cases in treatment and control groups, 27（71.1％） and 13（34.2％）had remarkable improvement in their symptoms. The therapeutic effect of treatment group was significantly superior to that of control group（*P*＜0.01）. The developmental quotient values of speech of treatment and control groups were 56.36±19.77 and 46.96±15.63 respectivelydisplaying that acupuncture could significantly improve cerebral palsy patients’ speaking ability（*P*＜0.05）. Conclusion: The therapeutic effect of acupuncture therapy plus linguistic training is significantly superior to that of simple linguistic training.

1. **Li HM. Acupuncture-based therapy for paediatric cerebral palsy speech disorders//Guangdong Provincial Acupuncture and Moxibustion Society. Compilation of papers from the 10th Academic Exchange Conference of Guangdong Province Acupuncture and Moxibustion Society. Children's Rehabilitation Centre, Boai Hospital, Zhongshan;2007:3.**

**Title:** Acupuncture-based therapy for paediatric cerebral palsy speech disorders

**Authors:** Li HM

**Abstract:** Paediatric cerebral palsy can be associated with mental retardation, epilepsy, behavioural abnormalities and language and perceptual disorders. According to relevant data, the incidence of cerebral palsy in China is 4/1000-18/1000, and 80% of them have different degrees of language impairment. In 2000/2002, the use of acupuncture and oral function training to promote the recovery of speech disorders in children with cerebral palsy achieved satisfactory results. The results are reported as follows.

1. **Yang YQ. Analysis of the therapeutic effects of scalp acupuncture and speech training on cerebral palsy speech disorders and related factors. Heilongjiang: Jiamusi University 2007.**

**Title:** Analysis of the therapeutic effects of scalp acupuncture and speech training on cerebral palsy speech disorders and related factors

**Authors:** Yang YQ

**Abstract:** Object: This study’s aim is to determine that scalp acupuncture in combine with speech treatment is effective and necessary for speech disorders in children witll cerebral palsy. Then, to explore the relationship between the effect of speech training and scalp acupuncture with speech disorder type, the degree of the illness and the level of mental retardation, find a effective way to treat cerebral palsy(CP) in children with language and speech disorder. Methods：This study was conducted among these children with cerebral palsy during one year period from Mac 2006 to Mac 2007, a total of 215 cerebral palsy children 164 with speech and language disorder, we selected 145 suitable cases from them．All patients were asked personal history and examined by CRRC<s-s>and dysarthria scale and intelligent test at the beginning of rehabilitation, they were examed by brainstem auditory evoked potential(BAEP) and brain CT test to exclude auditory disorder. 30 cases belong to contrast group, 76 cases belong to speech and language training group, 49 cases belong to scalp acupuncture in combine with speech and language training group. Contrast group only adopted sythesis rehabilitation therapy(not contain speech therapy and scalp acupuncture), there were no significant difference in age, gender, the type of cerebral palsy, the type of speech and language disorder, the level of founetion and the level of mental retardation between three groups. Three months is a period of treatment, after a period oftreatment, we observed the effect and analysed related factors. Resuits: 1. The results: All patients Were examed by CRRC<s-s>and dysarthriatest at the end of treatment, The results showed difference Was significant(*P*<0．01), the curative effect of speech and language training group. scalp acupuncture in combine with speech and language training group was more excellent than that of contrast group. The total effective rate of contrast group is 60％, speech and language training group is 88.4％, scalp acupuncture in combine with speech and language training group is 95.9％．2．Related factors analysis: The statistical analysis demonstrated that the therapeutic effeetivness was related to some factors such as the type of speech and language disorder, the level of founction and the 1evel of mental retardation. The results shewed the effect of speech bradygenesis was more excellent than that of the other two types(*P*<0.01), moreover there was not difference between the latter two types stastically(*P*>0.05). The results showed the difference was significant among them(*P*<0.01), The higher the level of founction is and the higher the score of intelligent is, The better the effectivness is achieved．Conclusions: The curative effect of speech and language training group. scalp acupuncture in combine with speech and language training group was more excellent than that of contrast group, and the scalp acupuncture in combine with speech and language training group is more effective. Scalp acupuncture unite speech training can improve the effect more significantly, it is a good method to treat cerebral palsy with language disorder and is good for spreading widely for clinic. 2. Related factors analysis: The effect of speech bradygenesis was more effective than other two types. The higher the level of founction is and the higher the Score ofintelligent is, The better the effectivness is achieved．

1. **Jiang ZH. Clinical observation of acupuncture combined with language training in the treatment of language disorders in children with cerebral palsy. Modern Nurse 2009;(6):26-27.**

**Title:** Clinical observation of acupuncture combined with language training in the treatment of language disorders in children with cerebral palsy

**Authors:** Jiang ZH

**Abstract:** Objective: To explore the best treatment method for children with cerebral palsy combined with speech disorders, and to improve the language ability of the children. Methods: 93 cases of children with cerebral palsy combined with language disorders were randomly divided into acupuncture group and control group for clinical observation, and both groups of children underwent language training and systematic rehabilitation treatment, with the acupuncture group adding the acupuncture at the Sishen points, Zhi san zhen, Tongue Three Needles, Brain Three Needles, the Second Area of the Speech, the Feng-chi, and the Ya-men as the main acupuncture; and the treatment of the two groups was carried out for 4 months. Results: The total effective rate of the acupuncture group was 95.8%, and the total effective rate of the control group was 78.3%, the difference was statistically significant (*P*<0.01). Conclusion: In the rehabilitation treatment of cerebral palsy combined with language disorder, the therapeutic effect of acupuncture combined with language training was significantly better than that of the control group with language training alone.

1. **Li HW, Ma BX, Feng Y. Clinical observation on scalp acupuncture for treatment of speech disorders in paediatric cerebral palsy. Chinese Medicine Modern Distance Education of China 2010;8(24):37-38.**

**Title:** Clinical observation on scalp acupuncture for treatment of speech disorders in paediatric cerebral palsy

**Authors:** Li HW, Ma BX, Feng Y

**Abstract:** Objective: To observe the clinical efficacy of scalp acupuncture in the treatment of cerebral palsy (cerebral palsy) speech disorder. Methods: 61 cases of children who met the diagnostic criteria of cerebral palsy and combined with speech disorders were randomly divided into 31 cases in the treatment group and 30 cases in the control group. In the treatment group, scalp acupuncture was used, and in the control group, no scalp acupuncture was used. Both groups were given speech training and basic treatment (tuina massage, exercise therapy and physiotherapy) for 12 weeks. Both groups were given speech training, basic treatment (tuina massage, exercise therapy, physiotherapy), and the treatment course was 12 weeks. Results: ①The children in both groups showed improvement in their speech disorders after treatment, with a total effective rate of 77.42% in the treatment group and 63.33% in the control group, and the difference between the two groups was significant (*P*＜0.05). The total effective rate of cerebral palsy children with speech disorder in the age group of 1-3 years was 80.00%, which was higher than that in the age group of 3-6 years, and the difference was statistically significant. Conclusion: Both groups improved the speech disorders of children with cerebral palsy, and the treatment group was better than the control group. For cerebral palsy speech disorders, the efficacy of treatment is good up to the age of 3 years, so early treatment is the key to this disease.

1. **Li XJ, Yang YQ, Pang W, Zhang SL, Chen BK. The analysis of the effects and related factors of head acupuncture combined with speech therapy in treating the language disorder of cerebral palsy children. Chinese Pediatrics of Integrated Traditional and Western Medicine 2010;2(4):326-330.**

**Title:** The analysis of the effects and related factors of head acupuncture combined with speech therapy in treating the language disorder of cerebral palsy children

**Authors:** Li XJ, Yang YQ, Pang W, Zhang SL, Chen BK

**Abstract:** Objective: To observe the curative effect of head acupuncture combined with speech therapy and analyze the relations among the types of speech disorderdegree of illness and level of mental retardation．Methods: Selected 155 cerebral palsy（CP）children through the examination of CRRC（s-s）, dysarthria scale and intelligent test before and after treatment；all patients were excluded auditory disorder. Three groups were: 30 for cont rast group（group1）, 76 for speech and language training group（group2） and 49 for head acupuncture combined with speech and language training group（group3）. Three months was one treatment period. Results:（1）There was a significant difference on the effective rates（*P*＜0.01） with 60% for group188.4％ for group2 and 95.9％ for group 3；（2）It was more effective for delayed language development disorder（DLDD）than others（*P*＜0.01）. The higher the level of function was the higher the score of intelligence wasthe better the effect was achievedwith significant difference（*P*＜0.01）. Conclusions:（1）The therapy has curative effects in group2 and group3while group3 is more effective；（2）The DLDD is more effective than the others；（3）The better the curative effectthe higher the level of function and the higher the score of intelligence．

1. **Liang B, Ouyang BS, Pu YP. Observation on the efficacy of scalp acupuncture with needle speech training in the treatment of speech dysfunction in paediatric cerebral palsy. Chinese Pediatrics of Integrated Traditional and Western Medicine 2010;2(01):21-23.**

**Title:** Observation on the efficacy of scalp acupuncture with needle speech training in the treatment of speech dysfunction in paediatric cerebral palsy

**Authors:** Liang B, Ouyang BS, Pu YP

**Abstract:** Objective: To observe the efficacy of scalp acupuncture with needle speech training in treating speech disorders in children with cerebral palsy (referred to as cerebral palsy). Methods: 61 children with cerebral palsy were randomly divided into observation group and control group, and both groups underwent overall functional rehabilitation and language therapy in the same environment, the observation group underwent scalp acupuncture therapy, followed by training with needles, while the control group did not undergo scalp acupuncture therapy. 3 courses of treatment were completed to observe the rehabilitation of language functions such as receptive language ability, expressive language ability, IQ measurement, phonological disorders, and the clinical efficacy of the overall rehabilitation of language in the two groups. Results: The clinical efficacy of the observation group was better than that of the control group in terms of language acceptance, expression, comprehension, assessment of dysarthria, and language rehabilitation, and the difference was statistically significant (*P*＜0.05); however, there was no statistically significant difference between the two groups in terms of the improvement of IQ (*P*＞0.05). Conclusion: Scalp acupuncture combined with speech therapy and other comprehensive therapeutic techniques have a better effect on the rehabilitation of language function of children with cerebral palsy than simple rehabilitation.

1. **Zou XY, Yu ZH, He YM, Yang H, Dong XL. Effect of Acupuncture Combined Language Training on Cerebral Palsy Children with Language Retardation. Chinese Journal of Integrated Traditional and Western Medicine 2013;33(07):924-926.**

**Title:** Effect of Acupuncture Combined Language Training on Cerebral Palsy Children with Language Retardation

**Authors:** Zou XY, Yu ZH, He YM, Yang H, Dong XL

**Abstract:** Objective: To observe effects of acupuncture combined speech therapy for cerebral palsy children with linguistic retardation. Methods: Totally 132 cerebral palsy children were randomly assigned to the speech training group (Group A, 44 cases) and the routine acupuncture combined speech training group (Group B, 44 cases), and the acupuncture combined speech training group (Group C, 44 cases). Patients in Group A received one to one training including game therapy, therapy of communication attitudes, and so on. Those in the other two groups were needled at Baihui (GV20), Sishencong (EX-HN1), the first language zone, the second language zone, and the third language zone. Those in Group B were treated with electric needling and then speech training. Those in Group C were treated with language training, while needling with needle maintaining for 40 min. All patients were treated once daily, 5 times per week, 20 times as one course of treatment, 6 courses in total. The efficacy was assessed using S-S phonetic speech developmental retardation examination (CRRC version). The development quotient (DQ) was observed referring to the Gesell intellectual development scale before treatment, after 3 and 6 treatment courses. Results: Compared with Group A (the total effective rate: 51.3%, DQ value: 58.1±13.3), better effects were obtained in Group B (the total effective rate: 77.5%, DQ value: 60.4±13.5) and Group C (the total effective rate: 81.0%, DQ value: 64.0±11.6) (all *P* <0.05). There was no statistical difference in the total effective rate or post-treatment DQ value between Group B and Group C (*P* >0.05). Conclusion: Acupuncture combined speech therapy showed obvious effects on cerebral palsy children with linguistic retardation.

1. **Fan ZL, Yang Y. Clinical observation on acupuncture combined with linguistic training in treatment of cerebral palsy children with linguistic barrier. Maternal and Child Health Care of China 2014;29(34):5700-5701.**

**Title:** Clinical observation on acupuncture combined with linguistic training in treatment of cerebral palsy children with linguistic barrier

**Authors:** Fan ZL, Yang Y

**Abstract:** Objective: To observe the clinical curative effect of acupuncture combined with linguistic training in treatment of cerebral palsy children with linguistic barrier. Methods: A total of 100 children with cerebral palsy were randomly divided into treatment group and control group, 50 children in each group, the children in treatment group were treated with acupuncture combined with linguistic training, while the children in control group were treated with simple linguistic training. The children in the two groups were evaluated before treatment and after eight courses of treatment according to evaluation criteria of dysarthria and language retardation of "Chinese Diagnosis and Treatment Standardization of Rehabilitation Medicine" ; the curative effects in the two groups were compared. Results: After treatment，the total effective rates in treatment group and control group were 80. 0% and 70.0%, respectively, there was statistically significant difference ( *P*＜0. 05) . Conclusion: The effect of acupuncture combined with linguistic training in treatment of cerebral palsy children with linguistic barrier is significant, which is better than that of simple linguistic training, the method is worthy of clinical promotion and application.

1. **Jin YJ, Huang M, Li HX, Wang HY. Association between therapeutic effect of speech disorders and age on treatment of children with cerebral palsy. Chinese Journal of Applied Clinical Pediatrics 2014;29(24):1874-1876.**

**Title:** Association between therapeutic effect of speech disorders and age on treatment of children with cerebral palsy

**Authors:** Jin YJ, Huang M, Li HX, Wang HY

**Abstract:** Objective: To observe the association between therapeutic effect of speech disorder and the age on treatment of children with cerebral palsy, and to explore the best treatment period of speech disorder children with cerebral palsy. Methods: One hundred and twenty-one cases of speech disorder children with cerebral palsy aged 1 to 6 years in Maternal and Child Health Center of Hebei Province, Hebei Children’s Hospital, were selected as subjects, and the patients were randomly divided into training group, which included 64 cases who received scalp acupuncture combined with language and 57 cases treated with speech training group. After 6 courses of treatment, the therapeutic effect for speech disorder of children between 1 to 3 years old and > 3 to 6 years old were compared, the association between children’s age for treatment and treatment effects was explored. Results: The total treatment efficiency of 1 to 3 years and > 3 to 6 years old children in scalp acupuncture group combined with language training group were 95.45% and 83.33% respectively, the difference was significant(*X*^2^ =6.8598, *P* < 0. 05); while in 1 to 3 years and > 3 to 6 year-old children in speech training group the total treatment efficiency were 84.62% and 77.42%, and the difference was significant(*X*^2^= 6.6257, *P* < 0.05). In both groups, total efficiency of 1 to 3 years and > 3 to 6 years old children were 89.58% and 80.82%, respectively, and the difference was statistically significant(*X*^2^=9.6175 , *P* < 0. 01) . Conclusions: The best treatment period of speech disorder children with cerebral palsy is less than 3 years old, as age grows, the effect is gradually decreasing.

1. **Meng J, Zhou YM. Clinical study on treating language disorder in children with cerebral palsy by acupuncture. Clinical Journal of Chinese Medicine 2014;(5):66-67.**

**Title:** Clinical study on treating language disorder in children with cerebral palsy by acupuncture

**Authors:** Meng J, Zhou YM

**Abstract:** Objective: To investigate the research progress on treating language disorder in children with cerebral palsy by acupuncture. Methods: Selected from 1 June 2009 to 1 June 2013 into the hospital for treatment of 30 cases of cerebral palsy, cerebral palsy these 30 cases were randomly divided into two groups, 15 patients of each group, respectively, named for the observation group and control group. The two groups of children in the use of basic treatment, the observation group, and 15 cases were treated with acupuncture treatment, observed two groups of children with clinical outcomes. Results: In the control group, 15 cases were treated with basic treatment methods, effective in 7 cases, accounting for 46.7% of the number of clinical group, effective in 5 cases, accounting for the group 33.3% of the total number of clinical, 3 cases, accounting for the total number of the group of clinical 20%. The control group, the total effective clinical treatment was 80%. Observation group, 15 cases were treated with basic therapy based on further acupuncture treatment, 8 cases, accounting for 53.3% of the number of clinical group, effective in 5 cases, accounting for the group 33.3% of the total number of clinical, 2 cases, accounting for the group, 13.4% of the total number of clinical. Clinical observation group, the total effective rate was 86.6%. Conclusion: The two groups of children with cerebral palsy language barrier improvement observation group than the control group. A child with cerebral palsy using acupuncture treatment effect is obvious language barriers, worthy of promotion.

1. **Yang HJ. Analysis of acupuncture treatment of language barriers of children with cerebral palsy. China Medicine and Pharmacy 2014;(7):106-107,117.**

**Title:** Analysis of acupuncture treatment of language barriers of children with cerebral palsy

**Authors:** Yang HJ

**Abstract:** Objective: To investigate the research progress of the acupuncture treatment of language barriers of children with cerebral palsy. Methods: Thirty children with cerebral palsy treated in our hospital from January 2013 to December 2013 were selected and randomly divided into 2 groups, with 15 children in each group. The two groups were named as the treatment group and the control group. The 15 children of the control group received conventional language training and the 15 patients of the treatment group received acupuncture treatment on the basis of conventional language training. The clinical treatment effects of the two groups were observed. Results: The total effective treatment rate of the treatment group was 96.8% and that of the control group was 77.4%. The total effective treatment rate of the treatment group was significantly higher than that of the control group, with statistical significance between the 2 groups (*P*＜0.05). Conclusion: Acupuncture treatment shows good effects for the language barriers of children with cerebral palsy, thereby worthy of clinical promotion.

1. **Li DD, Qin JF, Xu X, Luo L. Clinical observation of acupuncture combined with language training on cerebral palsy the language barrier. //Proceedings of the 10th Beijing International Rehabilitation Forum. 2015:1666-1669.**

**Title:** Clinical observation of acupuncture combined with language training on cerebral palsy the language barrier

**Authors:** Li DD, Qin JF, Xu X, Luo L

**Abstract:** Objective: Before and after treatment by S-S language retardation examination and dysarthria evaluation, to explore the acupuncture combined with speech training. The treatment efficiency of children with language disorder of cerebral. Methods: 90 cases of spastic cerebral palsy children with language disorder were randomly divided into treatment group and control group with 45 cases in each group, the treatment group were treated by acupuncture combined with speech training, the control group using a simple speech training, after 3 courses of treatment of children with language function improvement and the treatment group patients age and curative effect. Results: The total effective rate of treatment group was 82.2%, control group total effective rate was 60%, there were statistically significant differences between the two groups (*P*<0.05); the curative effect of the treatment group in different age groups, the difference was statistically significant(*P*<0.05). Conclusion: Acupuncture combined with speech training for treatment of cerebral palsy with language disorder is superior to that of simple speech training; and the age, the better the prognosis.

1. **Li LY, Dong SS, Liu ZH. Study on laser acupuncture in children with mental retardation and comorbid language disorders. Chinese Journal of Acupuncture and Moxibustion(Electronic Edition) 2015;(3):105-108.**

**Title:** Study on laser acupuncture in children with mental retardation and comorbid language disorders

**Authors:** Li LY, Dong SS, Liu ZH

**Abstract:** Objective: The objective of this study was to observe the clinical curative effect of laser acupuncture treatment for mental retardation in children with language barrier. Methods: Forty children with mental retardation and comorbid language barrier hospitalized in rehabilitation department of Guangzhou University of Traditional Chinese Medicine Affiliated Nanhai Women and Children's Hospital from October 2013 to October 2014 were selected, and then divided into treatment group (n=20) and control group (n=20) according to random number table. The treatment group received laser acupuncture treatment on the basis of the basic rehabilitation combined with language training, while the control group was treated with basic rehabilitation combined with language training, once every other day. Twenty days made up one course, and three consecutive courses were observed in two groups. Beijing Gesell language development quotient (Gesell language DQ) was used for data analyze before and after treatment in two groups. The chi-square test was used for efficient comparison. Data of Gesell language DQ before and after treatment were measurement data, and expressed with $\overline{\text{x}}$±s. Data of Gesell language DQ within groups before and after treatment were compared with paired samples t-test, while two independent sample t-test was used between two groups. *P*<0.05 denotes the difference possessing statistical significance. Results: The total effective rate was 85% (17/20) in treatment group. The total effective rate was 55% (11/20) in control group. There was a significant difference in the total effective rate between two groups (*X*^2^=4.28, *P*=0.04 ). The Gesell language DQ was significantly higher in treatment group than that of the control group after treatment ( *t*=2.507, *P*=0.02 ). Conclusion: laser acupuncture treatment shows better curative effects on mental retardation children with language disorder, which is worth the clinical promotion.

1. **Li MJ. Correlation study between efficacy of speech disorder of children with cerebral palsy and treatment age. World Latest Medicine Information 2015;15(71):3-4.**

**Title:** Correlation study between efficacy of speech disorder of children with cerebral palsy and treatment age

**Authors:** Li MJ

**Abstract:** Objective: To study correlation between efficacy of speech disorder of children with cerebral palsy and treatment age. Methods: Divide 160 cases children with cerebral palsy randomly into observation group (received scalp acupuncture combined with speech training treatment) and control group (only speech training treatment) , compare speech disorder treatment effect of two groups with different age children with language. Results: Total effective rate of observation and control group of 1-3 years old children with speech disorder is higher than >3-6 years old children of the same group, and *P*<0.05. in addition, total effective rate of speech disorder children of 1-3 years and >3-6 years in observation group was higher than control group, and *P*<0.05. Conclusion 1-3 years age is the best treatment time for children with cerebral palsy, and the younger children has the better treatment effect, and vice versa.

1. **Liu Y, Shi SH. Clinical observation on 60 cases of speech disorders in paediatric cerebral palsy treated with integrated acupuncture combined with speech training. Inner Mongolia Journal of Traditional Chinese Medicine 2015;34(10):61-62.**

**Title:** Clinical observation on 60 cases of speech disorders in paediatric cerebral palsy treated with integrated acupuncture combined with speech training

**Authors:** Liu Y, Shi S

**Abstract:** Objective: To observe the clinical efficacy of acupuncture combined with speech training in treating speech disorders in paediatric cerebral palsy. Methods: 60 patients with cerebral palsy combined with speech disorders were randomly divided into 30 cases in the treatment group and 30 cases in the control group; the treatment group was treated with comprehensive acupuncture combined with speech training, and the control group was treated with simple speech training. Results: The total effective rate of the treatment group was 76.67%, and the total effective rate of the control group was 63.33%, and the comparison of the two groups was *P*<0.05. Conclusion: Acupuncture and moxibustion combined with speech training have better efficacy in treating speech disorders of paediatric cerebral palsy.

1. **Wang YL, Gao C, Song LX. Clinical observation on the treatment of speech disorders in paediatric cerebral palsy by the acupuncture method of benefit enlightenment. China Practical Medicine 2015;(12):278-280.**

**Title:** Clinical observation on the treatment of speech disorders in paediatric cerebral palsy by the acupuncture method of benefit enlightenment

**Authors:** Wang YL, Gao C, Song LX

**Abstract:** Objective: To explore an ideal therapy for treating speech disorders in children with cerebral palsy. Methods: Sixty children with cerebral palsy and concurrent speech disorders were randomly divided into a treatment group (acupuncture + speech training) and a control group (speech training only), with 30 children in each group. After two courses of treatment, the therapeutic effects were compared and observed. Results: The total effective rate in the treatment group was 93.3%, while the total effective rate in the control group was 73.3%. There was a statistically significant difference in therapeutic effect between the two groups (*P*<0.05). Conclusion: The combination of the acupuncture method of benefit enlightenment and speech training exhibits significant efficacy in treating speech disorders in children with cerebral palsy and can be clinically promoted and applied.

1. **Zhang Q. Exploring the clinical effects of acupuncture in the treatment of language disorders in children with cerebral palsy. Guide of China Medicine 2015;13(01):224-225.**

**Title:** Exploring the clinical effects of acupuncture in the treatment of language disorders in children with cerebral palsy

**Authors:** Zhang Q

**Abstract:** Objective: To investigate the clinical effect of acupuncture in treating speech disorders in children with cerebral palsy. Methods: Ninety cases of children with cerebral palsy who were admitted to our hospital from January 2012 to January 2013 were randomly divided into two groups of 45 children each, the treatment group and the control group. The 45 children in the control group were treated with basic treatment, and the 45 children in the treatment group were treated with acupuncture on top of the basic treatment. The clinical effects of the two groups were observed. Results: The total effective rate of clinical treatment for children in the treatment group was 95.6%. In the control group, the total effective rate of clinical treatment was 86.7%. Conclusion: Acupuncture is effective in treating speech disorders in children with cerebral palsy and is worth promoting.

1. **Wang YL. Observation on the efficacy of waking up the brain and opening up the orifices acupuncture combined with oral function training in the treatment of cerebral palsy with speech dysfunction. Modern Journal of Integrated Traditional Chinese and Western Medicine 2016;25(35):3962-3964.**

**Title:** Observation on the efficacy of waking up the brain and opening up the orifices acupuncture combined with oral function training in the treatment of cerebral palsy with speech dysfunction

**Authors:** Wang YL

**Abstract:** Objective: To observe the clinical efficacy of cerebral palsy with speech dysfunction in children with cerebral palsy treated with oral function training in combination with waking up the brain and opening up the orifices acupuncture. Methods: 88 children with cerebral palsy with speech dysfunction were randomly divided into 2 groups, both of them were given oral vitamin D. On the basis of this, 44 cases in the control group were given oral function training, and 44 cases in the study group were given oral function training combined with the waking up the brain and opening up the orifices acupuncture, and the clinical therapeutic effects and the changes of electroencephalography monitoring results, language expression ability, intelligence assessment, and dysarthria in the 2 groups before and after treatment were observed. Results: The total effective rate of the study group was significantly higher than that of the control group (*P*＜0.05). After treatment, the EEG of the two groups improved significantly (*P* < 0.05), and the scores of language expression ability, intelligence, and dysarthria increased significantly (*P* < 0.05), and the improvement of all indexes in the study group was significantly better than that in the control group (*P* < 0.05). Conclusion: Waking up the brain and opening up the orifices with acupuncture combined with oral function training can significantly improve the clinical symptoms of children with cerebral palsy and speech dysfunction, increase the overall effective rate, and improve the speech function, which is worthy of clinical promotion and application.

1. **Yu JJ. Clinical Research Oil the Effects of Acupuncture and Speech Training on Language Retardation in Children with Cerebral Palsy. Chinese Scientific Journal of Hearing and Speech Rehabilitation 2016;14(1):31-34.**

**Title:** Clinical Research Oil the Effects of Acupuncture and Speech Training on Language Retardation in Children with Cerebral Palsy

**Authors:** Yu JJ

**Abstract:** Oblective: To study the effects of acupuncture and speech training on language retardation in children with cerebral palsy. Methods: Sixty-one subjects with cerebral palsy were outpatients from Beijing Children’s Hospital from February 2014 to February 2015, and divided into 2 groups with 31 cases in treatment group and 30 cases in control group. The control group received speech training only and the treatment group received acupuncture and speech training. The clinical outcomes of the two groups were observed and compared. Results: Among the control group, the treatment was markedly effective for 13 cases(43.3％), effective for 11 cases(36.7％) and ineffective for 6 cases(20.0％), so the total effective rate for the control group was 80.0％. Among the treatment group, the treatment was markedly effective for 16 cases (51.6％), effective for 12 cases(38.7％) and ineffective for 3 cases(9.7％), so the total effective rate for the treatment group was 90.3％. The total effective rate of the treatment group was significantly higher than that of the control group(*X*^2^ =4.657, *P*<0.05). Conclusion: The improvement of the treatment group is more significant than the control group. The combined use of acupuncture and speech training is effective in treating language retardation of children with cerebral palsy, so it is worthy of further clinical application．

1. **Guo CG, Zhang HJ, Liu CL, Yan H, Hu JH, Zhou PQ. Clinical observation of the combined treatment of needle technique 'Congnao Tongluo' and speech training for children with cerebral palsy and intellectual disabilities. Guiding Journal of Traditional Chinese Medicine and Pharmacy 2016;22(18):74-76.**

**Title:** Clinical observation of the combined treatment of needle technique 'Congnao Tongluo' and speech training for children with cerebral palsy and intellectual disabilities

**Authors:** Guo CG, Zhang HJ, Liu CL, Yan H, Hu JH, Zhou PQ

**Abstract:** Objective: To observe the clinical efficacy of Congnao Tongluo acupuncture therapy combined with speech training in the treatment of cerebral palsy with intellectual disability in children. Methods: A total of 92 patients were randomly divided into a needle-rehabilitation group and a rehabilitation-only group, with 46 patients in each group. The needle-rehabilitation group received Congnao Tongluo acupuncture therapy combined with speech training, while the rehabilitation-only group received speech training alone. The intellectual levels and overall effective rates of the two groups were compared. Results: There were statistically significant differences in intellectual levels between the two groups after treatment compared with before treatment (*P*<0.05). Additionally, the needle-rehabilitation group showed better intellectual levels and overall effective rates than the rehabilitation-only group (*P*<0.05 or *P*<0.01). Conclusion: Congnao Tongluo acupuncture therapy combined with speech training is superior to speech training alone in terms of QD scores and clinical efficacy in the treatment of cerebral palsy with intellectual disability in children.

1. **Li SZ, Yang ZX, Li XJ, Wang YQ, Yuan HX. Analysis of the clinical efficacy of scalp acupuncture combined with language training in the treatment of language disorders in children with cerebral palsy and its influencing factors. Neural Injury and Functional Reconstruction 2016;11(6):555-557.**

**Title:** Analysis of the clinical efficacy of scalp acupuncture combined with language training in the treatment of language disorders in children with cerebral palsy and its influencing factors

**Authors:** Li SZ, Yang ZX, Li XJ, Wang YQ, Yuan HX

**Abstract:** Objective: To explore the clinical efficacy and influencing factors of scalp acupuncture combined with language training in the treatment of language disorders in children with cerebral palsy. Methods: A total of 120 children with cerebral palsy and language disorders were randomly divided into a control group and an observation group, with 60 children in each group, using a random number table method. Both groups received conventional rehabilitation treatments such as physiotherapy, occupational therapy, and massage. The control group received additional language training on the basis of conventional rehabilitation treatment, while the observation group received additional scalp acupuncture therapy on the basis of the control group's treatment for a period of 3 months. The clinical efficacy of the two groups was evaluated and compared, and the relationships between language disorder types, severity of illness, and intelligence levels of children in the observation group and their clinical efficacy were analyzed. Results: The clinical total effective rate in the observation group was 95.0%, which was higher than the 78.33% in the control group (*P*<0.05). The clinical total effective rate for children with developmental dysphasia in the observation group was significantly better than that for children with pre-verbal stage disorders and those with developmental dysphasia combined with articulatory disorders (*P*<0.05). The clinical total effective rate for children with normal intelligence levels was significantly higher than that for children with intellectual disabilities (*P*<0.05). The clinical total effective rate for children with mild severity of illness was significantly higher than that for children with moderate or severe severity (*P*<0.05). Spearman's rank correlation analysis showed a negative correlation between the severity of illness and the clinical total effective rate (*P*<0.05). Conclusion: Scalp acupuncture combined with language training has significant clinical efficacy in the treatment of language disorders in children with cerebral palsy. The types of language disorders, intelligence levels, and severity of illness have notable impacts on treatment outcomes.

1. **Liao JS. Exploring the efficacy of acupuncture with language training on children with cerebral palsy with delayed language development. Modern Diagnosis and Treatment 2016;27(15):2775-2776.**

**Title:** Exploring the efficacy of acupuncture with language training on children with cerebral palsy with delayed language development

**Authors:** Liao JS

**Abstract:** To investigate the therapeutic effect of acupuncture combined with language training on language developmental delay in children with cerebral palsy. A total of 78 children with cerebral palsy and language developmental delay admitted to the hospital were randomly divided into an observation group and a control group, with 39 children in each group. Both groups received conventional treatment for cerebral palsy. The control group received language training on the basis of conventional treatment, while the observation group received acupuncture combined with language training on the basis of conventional treatment. The therapeutic effects, as well as the developmental quotient (DQ) and oral motor function before and after treatment, were compared between the two groups. The total effective rate in the observation group was significantly higher than that in the control group (*P*<0.05). There were no significant differences in DQ scores and oral motor function scores between the two groups before treatment (*P*>0.05). After treatment, the DQ scores and oral motor function scores in the observation group were significantly higher than those in the control group (*P*<0.05). Acupuncture combined with language training can effectively improve the DQ and oral motor function of children with cerebral palsy and language developmental delay, thereby enhancing the therapeutic effect.

1. **Qin JF, Li DD. Clinical Observation of Acupuncture plus Speech Training for Speech Disorders in Cerebral Palsy. Shanghai Journal of Acupuncture and Moxibustion 2016;35(4):421-422.**

**Title:** Clinical Observation of Acupuncture plus Speech Training for Speech Disorders in Cerebral Palsy

**Authors:** Qin JF, Li DD

**Abstract:** Objective: To observe the therapeutic efficacy of acupuncture plus speech training for speech disorders in children with cerebral palsy based on the sign-significant relations (S-S) method for checking language development delay and dysarthria score. Method: Ninety patients with spastic cerebral palsy presenting with speech disorders were randomized into a treatment group and a control group by random number table, 45 cases in each group. The treatment group was intervened by acupuncture plus speech training, while the control group was by speech training alone. The improvement of speech function and the relationship between age and therapeutic efficacy were evaluated after 3 treatment courses. Result: The total effective rate was 82.2% in the treatment group versus 60.0% in the control group, and the difference was statistically significant (*P*＜0.05); in the treatment group, there were significant differences in comparing the therapeutic efficacy between different age groups (*P*＜0.05). Conclusion: Compared to speech training alone, acupuncture plus speech training can produce a better efficacy in treating speech disorders in cerebral palsy; the younger the patient, the better the prognosis.

1. **Tao F, Ding QY. Therapeutic effect of acupuncture on Fengchi, Tianzhu and Wuanjiao combined with language training in the treatment of speech disorders in children with cerebral palsy. Electronic Journal of Practical Clinical Nursing Science 2016;1(7):177-177,179.**

**Title:** Therapeutic effect of acupuncture on Fengchi, Tianzhu and Wuanjiao combined with language training in the treatment of speech disorders in children with cerebral palsy

**Authors:** Tao F, Ding QY

**Abstract:** Objective: To observe the efficacy of acupuncture at Fengchi, Tianzhu, and Wangu combined with language training in the treatment of language disorders in children with cerebral palsy. Methods: Sixty children with cerebral palsy and language disorders admitted to our hospital from May 2014 to April 2016 were selected as study subjects and randomly divided into a treatment group and a control group, with 30 children in each group. The treatment group received acupuncture combined with language training, while the control group received only language training. Both groups were assessed before treatment and after five courses of treatment, and the efficacy was compared between the two groups. Results: The total effective rate in the treatment group was 83.0%, and the total effective rate in the control group was 70.0%. The difference in efficacy between the two groups was statistically significant (*P*<0.05). Conclusion: The method of acupuncture at Fengchi, Tianzhu, and Wangu combined with language training for the treatment of language disorders in children with cerebral palsy is significantly more effective than the method of language training alone. It is worthy of promotion and application in clinical practice.

1. **Du X, Chen J, Jiang K, Wu ZF, Liang S. The Therapeutic Effect of Acupuncture on Cerebral Palsy in Children with Language Retardation Using the Midnight-noon Ebb-flow Method Combined with Syndrome Differentiation. Acupuncture Research 2017;42(4):346-349.**

**Title:** The Therapeutic Effect of Acupuncture on Cerebral Palsy in Children with Language Retardation Using the Midnight-noon Ebb-flow Method Combined with Syndrome Differentiation

**Authors:** Du X, Chen J, Jiang K, Wu ZF, Liang S

**Abstract:** Objective: To determine the clinical effect of acupuncture treatment on cerebral palsy in children with language retardation using the Midnight-noon Ebb-flow method combined with syndrome differentiation. Methods: One hundred and thirty-six children with cerebral palsy and language retardation were randomly divided into control group and treatment group. The control group received routine treatment for language rehabilitation, while the treatment group received acupuncture treatment using the Midnight-noon Ebb-flow method combined with syndrome differentiation for 3 months based on language rehabilitation. The children were assessed by sign-significate relations for language comprehension and language expression before and after treatment. Results: After treatment, the development quotient scores for language comprehension and language expression were (60.37±4.64) and (51.13±3.81) in the control group, and (73.54±4.73) and (64.08±3.93) in the treatment group, and the differences were statistically significant compared with those before treatment in the same one group, respectively (*P*<0.05, *P*<0.01) .The effects of the treatment group were statistically better than those of the control group (*P*<0.05). Conclusion: Acupuncture treatment using the Midnight-noon Ebb-flow method combined with syndrome differentiation has a better therapeutic effect in improving language comprehension and language expression, and promoting language development in children with cerebral palsy.

1. **Liu XL, Li YR, Song LJ. Curative effects carrying different treatments for cerebral palsy in children with development language delay. Chinese Journal of Applied Clinical Pediatrics 2017;32(11):867-869.**

**Title:** Curative effects carrying different treatments for cerebral palsy in children with development language delay

**Authors:** Liu XL, Li YR, Song LJ

**Abstract:** Objective: To explore effective treatment methods for cerebral palsy (CP) with delayed language development in children by utilizing three approaches: speech therapy alone, speech therapy combined with acupuncture, and speech therapy combined with acupuncture and acupoint injection. Methods: From March to September 2014, 90 children aged 2 to 5 years with CP and delayed language development, admitted to the Rehabilitation Department of Shanxi Children's Hospital, were selected and randomly divided into three groups using a random number table: a speech therapy group, a speech therapy combined with acupuncture group (treatment group 1), and a speech therapy combined with acupuncture and acupoint injection group (treatment group 2), with 30 children in each group. Corresponding clinical treatments were administered to each group. The Chinese version of the Child Language Development Assessment Method from the China Rehabilitation Research Center and the Gesell Developmental Scoring Method were used to evaluate the improvement of language disorders in the children. Results: The speech developmental quotient in the speech therapy group was higher after treatment [(55.47±9.12) points] than before treatment [(46.07±6.38) points], with a statistically significant difference (*t*=-10.67, *P*<0.01); the speech developmental quotient in treatment group 1 was higher after treatment [(57.80±9.18) points] than before treatment [(45.87±5.94) points], with a statistically significant difference (*t*=-9.42, *P*<0.01); the speech developmental quotient in treatment group 2 was higher after treatment [(65.16±9.02) points] than before treatment [(49.13±6.54) points], with a statistically significant difference (*t*=-13.77, *P*<0.01). The speech developmental quotient increased in all groups after treatment, and there was a statistically significant difference in the speech developmental quotient among the three treatment groups after treatment (*F*=8.604, *P*<0.01). Conclusion: Acupuncture combined with acupoint injection and speech therapy significantly improves the speech developmental quotient in children with CP and delayed language development, representing an effective treatment method for this condition.

1. **Shao XH. The clinical research of Jin’s 3-needles combined with speech therapy in treating the language disorder of cerebral palsy children. //Proceedings of the Academic Symposium of Cardiovascular Experts Invited by the Journal of Traditional Chinese Medicine and the Conference on Clinical Experience of Chinese Medicine in Treating Coronary Heart Disease and Angina Pectoris Disease. 2017:49-51.**

**Title:** The clinical research of Jin’s 3-needles combined with speech therapy in treating the language disorder of cerebral palsy children

**Authors:** Shao XH

**Abstract:** Objective: To observe the clinical effect of Jin's 3-needles combined with speech therapy training in treating the language disorder of cerebral palsy. Methods: Random allocation was taken to divide patients into 3 groups, Jin’s 3-needles group, language training group and the combined therapy group. Curative effect and cerebral blood flow were examined in post and pre-therapy stages for all the three groups. Results: The effective rate is 97.4% for the combined therapy group, significantly better than that of the other two groups （80.5% for Jin’s 3-needles and 77.5% for language training group）（*P*<0.05）. The result has statistical significance. The result has no statistical significance based on the comparison among Vs, Vm, RI of MCA, ACA, BA in the pre-therapy stage（*P*>0.05）. But in the post treatment stage, there is no big difference in Vs, Vm, RI of MCA, ACA, BA between Jin’s 3-needles group and the combined therapy group, but the result for those two groups is much better than that of language training group （*P*<0.05）. Conclusion: Jin’s 3-needles therapy could increase cerebral blood flow and could help restore brain function. The combined treatment of Jin’s 3-needles and language training can improve the curative effect of the language disorder of cerebral palsy children.

1. **Zhang YJ. Effect of acupuncture combined with language training on language development delay in children with cerebral palsy. Clinical Journal of Chinese Medicine 2017;9(06):90-91.**

**Title:** Effect of acupuncture combined with language training on language development delay in children with cerebral palsy

**Authors:** Zhang YJ

**Abstract:** Objective: To observe the effect of acupuncture combined with language training on language development delay in children with cerebral palsy. Methods: 76 cases of children with cerebral palsy treated in our hospital from March 2014 to March 2016 were selected, randomly divided into two groups, with 38 cases in each group. The control group was given language training, the observation group was given language training combined with acupuncture treatment, the improvement effect of acupuncture combined with language training on language development delay was compared. Results: After treatment, the total effective rate of the observation group was 92.1% (35/38), and that of the control group was 71.1% (27/38), the efficacy of the observation group was significantly higher than that the control group, the difference was statistically significant (*P* < 0.05). Conclusion: Acupuncture combined with language training on children with cerebral palsy could significantly relieve the language development delay symptoms, the curative effect was remarkable.

1. **Zhao XY. Clinical effect observation of acupuncture combined with language training in the treatment of language disorders in children with cerebral palsy. Clinical Research and Practice 2017;2(30):117-118.**

**Title:** Clinical effect observation of acupuncture combined with language training in the treatment of language disorders in children with cerebral palsy

**Authors:** Zhao XY

**Abstract:** Objective: To study the clinical curative effect of acupuncture com bined with language training in the treatment of language disorders in children with cerebral palsy. Methods: Sixty cases of children with language disorders and cerebral palsy in our hospital from February 2014 to January 2016 were selected as the subjects, and were randomly divided into control group and the observation group, with 30 cases in each group. The control group was given single language training, and the observation group received acupuncture treatment on the basis of the control group. The clinical efficacy of the two groups after treatment was observed and compared. Results: The total effective rate was 83.33% in the observation group and 60.00% in the control group. The total effective rate of the observation group was significantly higher than that of the control group, the difference between the two groups was statistically significant (*P*<0.05). Conclusion: Acupuncture combined with language training in children with cerebral palsy language disorder can significantly improve the language barrier of children, the effect is significant, it is worth popularizing in clinical practice.

1. **Bao LL. Analysis of the effects of Jin’s 3-needles combined with speech therapy on treating the language disorder of cerebral palsy children. Journal of Shandong First Medical University & Shandong Academy of Medical Sciences 2017;38(7):744-746.**

**Title:** Analysis of the effects of Jin’s 3-needles combined with speech therapy on treating the language disorder of cerebral palsy children

**Authors:** Bao LL

**Abstract:** Objective: To observe the clinical effect of Jin’s 3-needles combined with speech therapy training in treating the language disorder of cerebral palsy，and explore its mechanism． Methods: Random allocation was taken to divide patients into the 3 groups, Jin’s 3-needles group，language training group and the combined therapy group． Curative effect and cerebral blood flow were examined in post-therapy and pre-therapy stages for all the three groups. Results: The effective rate was 97.4% for the combined therapy group, which was significantly better than that of the other two groups ( 80.5% for Jin’s 3needles and 77.5% for language training group) ( *P*＜0.05). The result has no statistical significance( *P*＞0. 05) based on the comparison among Vs, Vm, RI of MCA, ACA, BA in the pre-therapy stage. But in the post-treatment stage，there was no big difference in Vs, Vm, RI of MCA, ACA, BA between Jin’s 3 needles group and the combined therapy group, but the result for those two groups was much better than that of language training group ( *P*＜0. 05)．Conclusion: Jin’s 3-needles therapy can increase cerebral blood flow and help restore brain function．The combined treatment of Jin’s 3-needles and language training can improve the curative effect of the language disorder of cerebral palsy children．

1. **Chen JJ. Analysis of the effect of rehabilitation therapy for language disorders in children with cerebral palsy. The Medical Forum 2017;21(03):341-343.**

**Title:** Analysis of the effect of rehabilitation therapy for language disorders in children with cerebral palsy

**Authors:** Chen JJ

**Abstract:** Objective: To explore the rehabilitation treatment effects on language disorders in children with cerebral palsy. Methods: A total of 100 pediatric patients with cerebral palsy and language disorders admitted to our hospital from January 2011 to December 2015 were selected as study subjects. These patients were randomly divided into a control group and an observation group using computer-generated randomization, with 50 patients in each group. The control group received language training rehabilitation treatment, while the observation group received acupuncture treatment in addition to language training. The rehabilitation effects were compared between the two groups. Results: The clinical total effective rate in the observation group was 96%, significantly higher than the 78% in the control group (*P*<0.05). Compared to before treatment, the intelligence scores of patients in both groups significantly improved after treatment (*P*<0.05), and the improvement was more pronounced in the observation group (*P*<0.05). Conclusion: The combination of language training and acupuncture treatment has significant rehabilitation effects in pediatric patients with cerebral palsy and language disorders, effectively improving patients' intellectual level.

1. **Chen SY. Analysing the clinical value of acupuncture and language training in the combined treatment of delayed language development in children with cerebral palsy. Health for Everyone 2018;(02):125.**

**Title:** Analysing the clinical value of acupuncture and language training in the combined treatment of delayed language development in children with cerebral palsy

**Authors:** Chen SY

**Abstract:** Objective: To explore the clinical treatment outcomes of applying acupuncture and language training in combination for the treatment of language developmental delay symptoms in children with cerebral palsy. Methods: 94 children with cerebral palsy admitted to our hospital for diagnosis and treatment were selected as study samples and randomly divided into two groups, namely the control group and the observation group, with 47 children in each group. All children received conventional basic treatment, followed by language training for the control group and acupuncture in addition to language training for the observation group. The clinical treatment effectiveness of the two groups was observed and compared. Results: The clinical treatment effectiveness rate in the observation group was 93.62%, showing a significant increase compared to the corresponding value of 82.98% in the control group (*P* < 0.05). Conclusion: The application of acupuncture and language training in combination for the treatment of children with cerebral palsy has a positive effect on improving their language developmental delay symptoms and is of great significance in improving the clinical treatment effectiveness rate of these children.

1. **Wu JJ, Zhang SY, Zhang XX, Hu YJ. A case-control study of group language training combined with acupuncture on children with cerebral palsy language disorder. Journal of Clinical Psychosomatic Diseases 2018;24(6):108-110,116.**

**Title:** A case-control study of group language training combined with acupuncture on children with cerebral palsy language disorder

**Authors:** Wu JJ, Zhang SY, Zhang XX, Hu YJ

**Abstract:** Objective: To study the clinical effect of group language training combined with acupuncture on children with cerebral palsy language disorder. Methods: 76 children with cerebral palsy language disorder were randomly divided into two groups, 38 cases in each group. The control group was treated with group language training, the observation group was treated with group language training combined with Xingnao Kaiqiao acupuncture, both groups were observed for 3 months. Clinical treatment effects of the two groups were compared. EEG situation and language function, intelligence assessment and dysarthria assessment of the two groups were compared as well. Results: After treatment, the EEG result of the observation group was better than that of the control group（*P*＜0.05）; the total effective rate in the observation group significantly higher than that in the control group（*P* ＜0.05）. After treatment, the language function, dysarthria assessment and intelligence assessment scores of the two groups were significantly in creased （*P*＜0.01）, and the observation group was significantly higher than the control group（*P*＜0.05 or 0.01）. Conclusion: Group language training combined with Xingnao Kaiqiao acupuncture in the treat ment of children with cerebral palsy language disorder can significantly improve the abnormal situation of EEG, improve the language function and intelligence level, with significant clinical effect.

1. **Zhao D, Shang Q, Ma CY. The effect of acupuncture with oral function training on the prognosis of 49 children with cerebral palsy and speech disorders. Guangming Journal of Chinese Medicine 2018;33(3):402-404.**

**Title:** The effect of acupuncture with oral function training on the prognosis of 49 children with cerebral palsy and speech disorder

**Authors:** Zhao D, Shang Q, Ma CY

**Abstract:** Objective: To investigate the impact of acupuncture combined with oral function training on the prognosis of children with cerebral palsy (CP) and language disorders. Methods: Ninety-eight children with CP and language disorders were randomly divided into a study group and a conventional group, with 49 children in each group using a random number table method. The study group received acupuncture plus oral function training, while the conventional group received only oral function training. The efficacy of the two groups was compared, and changes in motor function [Gross Motor Function Measure (GMFM)], intelligence, language behavior, and dysphasia scores were observed before and after treatment. Results: The total effective rate of treatment in the study group (91.84%) was higher than that in the conventional group (75.51%) (*P* < 0.05). Three months after treatment, the GMFM, intelligence, language behavior, and dysphasia scores of both groups increased compared to before treatment, and the scores in the study group were higher than those in the conventional group (*P* < 0.05). Conclusion: Acupuncture combined with oral function training can effectively improve the clinical symptoms of children with CP, enhancing their language, motor function, and intellectual abilities.

1. **Ai YF, Tao M, Liu YT, Gong ZK. The effect of acupuncture on the speech nerve centre in children with cerebral palsy. Chinese Journal of Trauma and Disability Medicine 2018;26(8):58-59.**

**Title:** The effect of acupuncture on the speech nerve centre in children with cerebral palsy

**Authors:** Ai YF, Tao M, Liu YT, Gong ZK

**Abstract:** Objective: To observe the therapeutic effect of acupuncture on the language neural center in children with cerebral palsy. Methods: Thirty children who met the diagnostic criteria for cerebral palsy and had concurrent language disorders were randomly divided into an observation group and a control group, with 15 children in each group. The observation group received scalp acupuncture treatment, while the control group did not receive scalp acupuncture treatment. Both groups received language therapy. Language ability was assessed using the S-S method before and after treatment to observe the clinical treatment effect. Results: After the treatment course, the language status of all 30 children improved. The total effective rate in the observation group was 93.3%, and the total effective rate in the control group was 46.7%. The difference between the two groups was statistically significant (*P* < 0.05). Conclusion: Acupuncture combined with language therapy has a good therapeutic effect on language disorders in children with cerebral palsy and is superior to language therapy alone.

1. **Huang YZ, Li XJ, Zhou L, Zhu Q, Wang T. Therapeutic effect of oral muscle training combined with scalp acupuncture on dysarthria in children with spastic cerebral palsy. Chinese Pediatrics of Integrated Traditional and Western Medicine 2019;11(6):502-505.**

**Title:** Therapeutic effect of oral muscle training combined with scalp acupuncture on dysarthria in children with spastic cerebral palsy

**Authors:** Huang YZ, Li XJ, Zhou L, Zhu Q, Wang T

**Abstract:** Objective: To explore the clinical efficacy of oral muscle training combined with scalp acupuncture in the treatment of dysarthria in children with spastic cerebral palsy. Methods: Sixty-six children with spastic cerebral palsy admitted to the Third Affiliated Hospital of Jiamusi University from June 2018 to June 2019 were randomly divided into experimental group and control group, 33 cases in each group. Both groups were treated for 6 months. The control group was trained in oral muscles. The experimental group received oral muscle training combined with scalp acupuncture treatment. The S-S language development retardation scale and the simple oral motor function scale were used to evaluate the language function and mouth movement of the two groups before and after treatment. Results: After 6 months of treatment, the speech developmental quotient and understanding development quotient of the two groups were significantly higher than those before treatment, and the improvement level of the experimental group was significantly higher than that of the control group(*P*<0.05). The level of function of the mandible, lip, tongue and the whole mouth was significantly higher than that before treatment, and the improvement level of the experimental group was significantly higher than that of the control group(*P*<0.05). Conclusion: Oral muscle training and scalp acupuncture treatment for dysarthria in children with spastic cerebral palsy can effectively improve children's language function and improve oral motor function, and has significant clinical efficacy.

1. **Jiao XB. Clinical effect of language rehabilitation combined with acupuncture on language barrier in children with cerebral palsy. Journal of Yan'an University(Medical Science Edition) 2019;17(3):57-59.**

**Title:** Clinical effect of language rehabilitation combined with acupuncture on language barrier in children with cerebral palsy

**Authors:** Jiao XB

**Abstract:** Objective: To explore the effects of language rehabilitation training combined with acupuncture on language function and development of children with cerebral palsy. Methods: A total of 111 children with cerebral palsy and language disorder who were hospitalized in the rehabilitation department of Luoyang Women and Children’s Health Care Center were randomly divided into two groups. Among them, 55 patients in the control group were treated with language rehabilitation alone, and 56 patients in the experimental group were added on the basis of language rehabilitations. The developmental status and language development quotient of children with cerebral palsy language barrier were compared. Results: After treatment, the behavioral scores, language expression and comprehension scores and total effective rate of the Gesell development scale in the experimental group were higher than those in the control group( *P*＜0.05). Conclusion: Language rehabilitation training combined with acupuncture can improve the development and language function of children with cerebral palsy with language disorders, and the clinical effect is remarkable.

1. **Li HZ. The effect of scalp acupuncture combined with verbal cognitive training on the intellectual development level of children with cerebral palsy. China Health Care & Nutrition 2019;29(33):336.**

**Title:** The effect of scalp acupuncture combined with verbal cognitive training on the intellectual development level of children with cerebral palsy

**Authors:** Li HZ

**Abstract:** Objective: To study and analyze the impact of scalp acupuncture combined with speech training on the intellectual development of children with cerebral palsy. Methods: 80 children with cerebral palsy who were treated in our hospital from January 2018 to December 2018 were selected and randomly divided into Group A (40 cases) and Group B (40 cases). Group A received speech training intervention, while Group B received scalp acupuncture intervention on this basis. The DQ (Developmental Quotient) scores and treatment effects before and after treatment were observed, recorded, and compared between the two groups. Results: The total clinical effective rates of Group A and Group B were 80.00% and 92.50% respectively, with Group B showing a higher rate when compared between the two groups (*P* < 0.05). Three months after treatment, the DQ scores of Group B were significantly higher than those of Group A (*P* < 0.05). Conclusion: For children with cerebral palsy, the combination of scalp acupuncture and speech training can effectively improve their intellectual development, which is beneficial for improving prognosis and has considerable application value in clinical practice.

1. **Yuan S, Ying YH, Zhang M. Study on the Treatment of Language Disorders in Preschool Children with Cerebral Palsy. Chinese Scientific Journal of Hearing and Speech Rehabilitation 2019;17(5):379-381,400.**

**Title:** Study on the Treatment of Language Disorders in Preschool Children with Cerebral Palsy

**Authors:** Yuan S, Ying YH, Zhang M

**Abstract:** Objective: To investigate the therapeutic effectof electroacupuncture combined with oral exercise therapy on language barrier in preschool children with cerebral palsy. Methods: From July 2017 to December 2018, 60 preschool cerebral palsy children were selected and treated in our hospital. The children were divided into the combined group and the control group according to the random number table method, 30 cases each. The control group was treated with oral exercise therapy, and the combined group was treated with electroacupuncture on the basis of the control group. The two groups were observed for 1 month, and the prognosis of the children was recorded. Results: The total effective rate of the combined group was 100.0%, which was significantly higher than that of the control group (76.7%) (*P*<0.05). The FMFM scores of the two groups were significantly higher than those before treatment (*P*<0.05), and the combined group scores were also higher than the control group (*P*<0.05). The scores of adaptation and language behavior after treatment were significantly higher than those before treatment (*P*<0.05), and the combination group was also significantly higher than the control group (*P*<0.05). The Vs and Vd of middle cerebral artery were significantly higher in the two groups than in the control group (*P*<0.05), and the combination group was significantly higher than the control group (*P*<0.05). Conclusion: Electroacupuncture combined with oral exercise therapy for the treatment of language barriers in preschool children with cerebral palsy can significantly improve the children's exercise and language barriers, improve treatment efficiency, and can be applied clinically.

1. **Lian HJ. Discussion on the effect of acupuncture combined with language training method in the treatment of speech delay due to cerebral palsy. Contemporary Medical Symposium 2020;18(5):32-33.**

**Title:** Discussion on the effect of acupuncture combined with language training method in the treatment of speech delay due to cerebral palsy

**Authors:** Lian HJ

**Abstract:** Objective: To explore the efficacy of acupuncture combined with language training in the treatment of children with language developmental delay caused by cerebral palsy. Methods: Eighty children with language developmental delay due to cerebral palsy who were diagnosed and treated at the Shanxi Rehabilitation Research Center between March 2014 and January 2019 were selected as study subjects. They were divided into a conventional group (n=40) and a combined group (n=40) using a random number table method. Children in the conventional group received language training, while those in the combined group received acupuncture combined with language training. The treatment efficacy and language developmental quotient (LDQ) scores were then compared between the two groups. Results: Compared with the conventional group, the combined group had a higher treatment efficacy, with *P*<0.05. After treatment, the LDQ scores of both groups significantly increased compared to before treatment, and the LDQ scores of the combined group were higher than those of the conventional group, with *P*<0.05. Conclusion: The application of acupuncture combined with language training in the treatment of children with language developmental delay caused by cerebral palsy can achieve significant efficacy and effectively promote the development of their language function.

1. **Lin W, Wang ML, Liang S. Clinical Study on the Treatment of Cerebral Palsy with Speech Disorders by Auricular Point Pressing Combined with Speech Training. World Latest Medicine Information 2020;20(32):196-197.**

**Title:** Clinical Study on the Treatment of Cerebral Palsy with Speech Disorders by Auricular Point Pressing Combined with Speech Training

**Authors:** Lin W, Wang ML, Liang S

**Abstract:** Objective: To study the clinical efficacy of auricular point pressing combined with speech training in the treatment of speech disorders in children with cerebral palsy. Methods: A total of 86 children with cerebral palsy and speech disorders were randomly divided into a speech training group and an auricular point pressing combined group, with 43 children in each group. The speech training group received conventional exercise therapy and speech training, while the auricular point pressing combined group received auricular point pressing therapy in addition to the speech training. The main acupoints used were Heart, Brain, and Tongue. Treatment was administered for 5 consecutive days followed by a 2-day rest, for a total of 3 months. The developmental quotient and severity of dysphasia were assessed before and after treatment in both groups. Results: After 3 months of treatment, improvements were observed in language behavior, gross motor skills, fine motor skills, social behavior, and adaptability in both groups compared to before treatment. The auricular point combined group showed better improvements than the speech training group (*P*<0.05), with a statistically significant difference. Comparison of dysphasia assessments before and after treatment showed that the auricular point pressing combined group had a higher effectiveness rate than the speech training group. Conclusion: The addition of auricular point pressing therapy to speech training can better improve the intellectual level and speech function of children with cerebral palsy, without increasing treatment time costs. It is worthy of promotion and application in clinical treatment.

1. **Liu L. The role of acupuncture with language training in children with cerebral palsy with delayed speech development. World Latest Medicine Information 2020;20(69):165-166.**

**Title:** The role of acupuncture with language training in children with cerebral palsy with delayed speech development

**Authors:** Liu L

**Abstract:** Objective: To observe and analyze the role of acupuncture combined with language training in the treatment of language developmental delay in children with cerebral palsy. Methods: A total of 150 children with cerebral palsy and language developmental delay admitted to our hospital from February 2018 to February 2019 were selected as study subjects and randomly divided into a control group and a study group, with 75 children in each group. The control group received language training, while the study group received acupuncture combined with language training. The effects of different intervention methods were compared. Results: The oral motor function scores of the children in the study group were significantly higher than those in the control group, with a statistically significant difference (*P*<0.05). Conclusion: For children with cerebral palsy and language developmental delay, acupuncture combined with language training can have a positive effect on improving their oral motor function and promoting the recovery of language function.

1. **Liu ML, Mei SX, Yang LD, Mao PA. Clinical observation on 54 cases of paediatric cerebral palsy combined with speech disorders treated by Tong Guan Li Qiao acupuncture combined with speech training. Journal of Pediatrics of Traditional Chinese Medicine 2020;16(06):101-104**

**Title:** Clinical observation on 54 cases of paediatric cerebral palsy combined with speech disorders treated by Tong Guan Li Qiao acupuncture combined with speech training

**Authors:** Liu ML, Mei SX, Yang LD, Mao PA

**Abstract:** Objective: To observe the clinical efficacy of Tongguan Liqiao acupuncture therapy combined with speech training in the treatment of children with cerebral palsy combined aphasis. Methods: From May 2016 to May 2019, 107 children with cerebral palsy combined aphasis admitted into rehabilitation department of Lishui The People’s Hospital were divided into control group with 53 cases and treatment group with 54 cases according to random single blind method. The control group was given individualized speech training program, the treatment group was supplemented with Tongguan Liqiao acupuncture therapy on the basis of the control group. After 3 months of treatment, we kept statistics about the clinical efficacy, and evaluated children’s activities of daily life( ADL) scale and gross motor function measure(GMFM) scale of the 2 groups. Results: The total effective rate of the control group was 77.36%( 41 /53), that of the treatment group was 92.59%( 51 /54), and the difference was statistically significant between the 2 groups( *P*＜0.05). The total effective rate of the 1-3 age bracket was higher than that of the 4-6 age bracket in the same group, and the difference was statistically significant( *P*＜0.05), moreover, the curative effect of the 1-3 age bracket in the treatment group was more significant( *P*＜0.05). There was no significant difference in GMFM and ADL scores between the 2 groups before treatment (*P*＞0.05), and they were comparable; after treatment, GMFM and ADL scores of the two groups were significantly improved, and the difference was statistically significant compared with the same group before treatment(*P*＜0.05), moreover, the GMFM and ADL scores of the treatment group more significantly improved(*P*＜0.05). Conclusion: It can significantly improve children’s language function, ADL and GMFM to use Tongguan Liqiao acupuncture therapy combined with speech training in the treatment of children with cerebral palsy combined aphasis, and the younger the age, the better the curative effect.

1. **Ma DD. Clinical observation on the treatment of cerebral palsy combined with delayed speech development by acupuncture combined with speech training using the meridian flow injection najia method. Clinical Research 2020;28(12):132-134.**

**Title:** Clinical observation on the treatment of cerebral palsy combined with delayed speech development by acupuncture combined with speech training using the meridian flow injection najia method

**Authors:** Ma DD

**Abstract:** Objective: To explore the efficacy of acupuncture based on the meridian flow injection najia method combined with language training in children with cerebral palsy and delayed language development. Methods: A total of 80 children with cerebral palsy and delayed language development admitted to our hospital from June 2018 to June 2019 were selected as observation subjects and randomly divided into an observation group and a control group, with 40 cases in each group. The control group received language training, while the observation group received acupuncture based on the meridian flow injection najia method in addition to language training. The intervention effects of the two groups were compared. Results: The treatment effective rate in the observation group was 95.00%, which was significantly higher than the 82.50% in the control group (*P*＜0.05). Before treatment, there was no significant difference in language comprehension and expression scores between the two groups (*P*＞0.05). After treatment, the language comprehension and expression scores in the observation group were significantly higher than those in the control group (*P*＜0.05). Conclusion: Acupuncture based on the meridian flow injection najia method combined with language training in children with cerebral palsy and delayed language development can effectively improve their language comprehension and expression abilities, enhance clinical treatment efficacy, and promote their rehabilitation.

1. **Song LJ. Clinical observation of acupuncture combined with language rehabilitation training in the treatment of cerebral palsy with speech delay. China's Naturopathy 2020;28(2):30-32.**

**Title:** Clinical observation of acupuncture combined with language rehabilitation training in the treatment of cerebral palsy with speech delay

**Authors:** Song LJ

**Abstract:** Objective: To explore the clinical efficacy of acupuncture combined with speech rehabilitation training in the treatment of children with cerebral palsy and delayed speech. Methods: A total of 66 children with cerebral palsy and delayed speech were selected and randomly divided into a control group and an observation group, with 33 children in each group. Both groups received basic treatment. The control group received speech rehabilitation training, while the observation group received acupuncture in addition to the treatment given to the control group for a continuous period of 3 months. The improvement in intelligence and oral motor function of the children in both groups was observed, and the clinical efficacy was compared. Results: After treatment, the ability to respond to people, objects, actions, and language in both groups was improved compared with before treatment (*P*<0.05), and these four indicators were more significantly improved in the observation group compared with the control group (*P*<0.05). After treatment, the oral motor function scores of both groups were higher than those before treatment (*P*<0.05), and the oral motor function score of the observation group was significantly higher than that of the control group (*P*<0.05). The total effective rate in the observation group was 87.88% (29/33), which was higher than the 66.67% (22/33) in the control group, with a statistically significant difference (*P*<0.05). Conclusion: Acupuncture combined with speech rehabilitation training has good clinical efficacy in the treatment of children with cerebral palsy and delayed speech, effectively improving the children's speech function and promoting intellectual development.

1. **Yu N. Analysis of the efficacy of speech training combined with electroacupuncture therapy on cerebral palsy with speech disorder. Kang Yi 2020;(12):180.**

**Title:** Analysis of the efficacy of speech training combined with electroacupuncture therapy on cerebral palsy with speech disorder

**Authors:** Yu N

**Abstract:** Objective: To evaluate the therapeutic effect of speech training combined with electroacupuncture in patients with cerebral palsy and speech disorders. Methods: For this study, 48 children with cerebral palsy and speech disorders admitted to our hospital from January 2018 to July 2019 were selected as research subjects. The children were randomly divided into two groups, with 24 children in each group. The control group received speech training, while the experimental group received speech training combined with electroacupuncture. The treatment outcomes of the two groups were statistically analyzed. Results: After treatment, the articulatory disorder scores of the children with cerebral palsy and speech disorders in the experimental group were significantly better than those in the control group, with statistical significance (*P*<0.05). The treatment efficacy rate of the children in the experimental group was significantly better than that of the control group, with statistical difference (*P*<0.05). Conclusion: Speech training combined with electroacupuncture for children with cerebral palsy and speech disorders can effectively improve their speech function and enhance their quality of life, which is clinically significant.

1. **Qiu L. Exploring the clinical efficacy of acupuncture with language training in the treatment of delayed language development in children with cerebral palsy. Heilongjiang Journal of Traditional Chinese Medicine 2021;50(06):37-38.**

**Title:** Exploring the clinical efficacy of acupuncture with language training in the treatment of delayed language development in children with cerebral palsy

**Authors:** Qiu L

**Abstract:** Objective: To observe the application effect of acupuncture and moxibustion combined with speech training in the treatment of language developmental delay in children with cerebral palsy. Methods: Sixty children with cerebral palsy and language developmental delay admitted to our hospital from January 2019 to December 2020 were randomly selected and grouped using a parallel control method. The control group (n=30) received speech training treatment, while the observation group (n=30) received acupuncture and moxibustion combined with speech training. The treatment effects of the two groups were compared. Results: In terms of scores for social interaction, motor ability, language ability, and object manipulation before treatment, there were small differences between the two groups (*P*>0.05). After treatment, the scores in the observation group were higher, with significant differences compared to the control group (*P*<0.05). In terms of the total effective rate of treatment, the observation group (90.00%) was higher than the control group (66.67%), with a significant difference (*P*<0.05). There were no significant differences in oral motor function scores between the two groups before treatment (*P*>0.05). After treatment, the observation group had significantly higher scores than the control group (*P*<0.05). Conclusion: Acupuncture and moxibustion combined with speech training have significant application effects in the treatment of language developmental delay in children with cerebral palsy. In addition to improving children's language function, it can also alleviate their language developmental delay and oral motor dysfunction, enhancing the overall treatment effect. This method is worthy of clinical adoption and promotion.

1. **Wang CX. Clinical efficacy observation of acupuncture combined with language training in treating speech disorders in paediatric cerebral palsy. Healthy Women 2021;(21):122.**

**Title:** Clinical efficacy observation of acupuncture combined with language training in treating speech disorders in paediatric cerebral palsy

**Authors:** Wang CX

**Abstract:** Objective: To analyze and study the clinical effect of acupuncture combined with speech training in the treatment of language disorders in children with cerebral palsy, in order to provide a meaningful reference for the treatment of this condition. Methods: The study inclusion period was set from March 2020 to March 2021. During this time, 70 children with cerebral palsy and language disorders treated in our hospital were selected and randomly divided into two groups using a computer-generated randomization method. The control group received speech training alone, while the experimental group received acupuncture in addition to speech training. The improvement of indicators in both the experimental and control groups was statistically analyzed. Results: After statistical analysis, the clinical treatment effect was significantly better in the experimental group, with statistically significant differences (*P*<0.05). Conclusion: The application of acupuncture combined with speech training in the treatment of language disorders in children with cerebral palsy can significantly improve various indicators of the patients and facilitate their recovery. Therefore, it is worth promoting the use of this method in children with cerebral palsy and language disorders.

1. **Yan TQ, Wang S, Guan LJ. Effects of acupuncture combined with language training on Frenchhay articulation score in children with cerebral palsy combined with dysarthria. Clinical Journal of Traditional Chinese Medicine 2021;33(5):957-960.**

**Title:** Effects of acupuncture combined with language training on Frenchhay articulation score in children with cerebral palsy combined with dysarthria

**Authors:** Yan TQ, Wang S, Guan LJ

**Abstract:** Objective: To explore the effect of acupuncture and language training on Frenchhay scores of children with cerebral palsy and dysarthria; Methods: To select 60 children with cerebral palsy and dysarthria, and randomly divide them into observation group and control group, each with 30 cases, control The group was given comprehensive language training, and the observation group was given acupuncture combined with language training. After the treatment, the GESELL development scale and Frenchhay articulation scores of the two groups were compared. Results: There was no statistical comparison between the scores of the two groups of children on the GESELL scale before treatment Significance (*P*>0.05). After treatment, the scores of the two groups of children in major motor, fine motor, language, and social interactions were improved compared with the previous ones. The language and social scores of the observation group were greater than those of the control group, and the difference was statistically significant (*P*<0.05); There was no statistically significant difference between the two groups of children’s articulation evaluation items (*P*>0.05). After treatment, except for the jaw position, the scores of the observation group were all higher than before, and the difference was statistically significant (*P*<0.05). The scores of breathing, lip movement, tongue movement, throat control, and speech comprehension were all higher than those of the control group, and the difference was statistically significant (*P*<0.05). Conclusion: Acupuncture combined with language training can improve oral function, improve articulation and communication skills.

1. **Yu N. Observation on the clinical efficacy of Congnao Tongluo Acupuncture combined with speech training in treating paediatric cerebral palsy with mental retardation. Clinical Nursing Research 2021;30(1):272-273.**

**Title:** Observation on the clinical efficacy of Congbrain Tongluo Acupuncture combined with speech training in treating paediatric cerebral palsy with mental retardation

**Authors:** Yu N

**Abstract:** Objective: To analyze the clinical efficacy of Congnao Tongluo acupuncture combined with speech training in the treatment of children with cerebral palsy accompanied by intellectual disability. Methods: Sixty children with cerebral palsy accompanied by intellectual disability who were treated in our hospital from January 2018 to January 2020 were selected and randomly divided into two groups with an equal number of patients. The control group received speech training alone, while the experimental group received Congnao Tongluo acupuncture combined with speech training. The effectiveness rate, intellectual level, and language function of the patients after treatment were observed and compared between the two groups. Results: The effective treatment rate, intellectual level, and language function of the children with cerebral palsy accompanied by intellectual disability in the experimental group were higher than those in the control group, with statistical significance (*P* < 0.05). Conclusion: The treatment of children with cerebral palsy accompanied by intellectual disability using Congnao Tongluo acupuncture combined with speech training is beneficial for improving the treatment outcome and has value for promotion.

1. **Jin YJ, Huang M. Comparison of two kinds of cephalic acupuncture therapy for speech disorder in children with cerebral palsy. Medicine and Health 2022;3:8-11.**

**Title:** Comparison of two kinds of cephalic acupuncture therapy for speech disorder in children with cerebral palsy

**Authors:** Jin YJ, Huang M

**Abstract:** Objective: To observe the curative effect of two different acupoints combination head acupuncture combined with speech therapy. Methods: Selected 190 cerebral palsy(CP) children randomly divided into three groups, 66 for routine treatment and language training group(control group), 63 for routine treatment and language training combined with Jin’s head acupuncture(Jin’s group), 61 for routine treatment and language training combined with Jiao’s head acupuncture (Jiao’s group). Results: The therapeutic effect of Jin’s group and Jiao’s group was obviously better than that of contrast group, There was a significant difference on the effective rates(P&lt;0.05); The therapeutic effect of Jiao’s group was better than that of Jin’s group, but there was no significant difference (P&gt;0.05). Conclusions: The therapy has curative effects in 3 groups, but The therapeutic effect of two different acupoints combination head acupuncture combined with speech therapy was obviously better than pure language training therapy; there was no significant difference in total effective rate between the two combinations of acupoints.

1. **Yang HY, Liu CP. Observation on the efficacy of scalp acupuncture with needle speech training in the treatment of speech dysfunction in paediatric cerebral palsy. ZHONGHUA YANGSHENG BAOJIAN 2022;40(18):60-62.**

**Title:** Observation on the efficacy of scalp acupuncture with needle speech training in the treatment of speech dysfunction in paediatric cerebral palsy

**Authors:** Yang HY, Liu CP

**Abstract:** Objective: To explore the efficacy of scalp acupuncture combined with in-needle speech training in treating language dysfunction in children with cerebral palsy. Methods: A total of 100 children with cerebral palsy and language dysfunction admitted to Dezhou Traditional Chinese Medicine Hospital from January 2019 to December 2020 were selected as study subjects. The children were divided into an experimental group and a control group using a random number table method, with 50 children in each group. The control group received conventional functional rehabilitation and speech therapy interventions, while the experimental group received scalp acupuncture combined with in-needle speech training on the basis of the control group's interventions. The treatment outcomes, intellectual development, changes in language function, and Gesell Developmental Scales scores were compared between the two groups. Results: The total effective rate in the experimental group was higher than that in the control group, with a statistically significant difference (*P*<0.05). After treatment, the dysarthria scores of both groups were lower than those before treatment, while the expressive language ability and receptive language ability scores were higher than those before treatment. Furthermore, the experimental group had lower dysarthria scores and higher expressive and receptive language ability scores compared to the control group, with statistically significant differences (*P*<0.05). After treatment, the Gesell scores for personal-social skills, language, adaptive behavior, and motor skills in both groups were higher than those before treatment, with statistically significant differences (*P*<0.05). After treatment, the experimental group had higher Gesell scores for personal-social skills, language, adaptive behavior, and motor skills compared to the control group, with statistically significant differences (*P*<0.05). Conclusion: Scalp acupuncture combined with in-needle speech training can improve the treatment outcomes and better alleviate language and intellectual impairments in children with cerebral palsy.

1. **Zhang W, He X, Huang Y. Effect of Language Rehabilitation Training Combined with Acupuncture in Children with Cerebral Palsy. Henan Medical Research,2022,31(3):485-488.**

**Title:** Effect of Language Rehabilitation Training Combined with Acupuncture in Children with Cerebral Palsy

**Authors:** Zhang W, He X, Huang Y

**Abstract:** Objective: To explore the effect of language rehabilitation training combined with acupuncture in children with cerebral palsy. Methods: A total of 92 children with cerebral palsy who were treated in Shangqiu First People's Hospital from October 2017 to December 2020 were selected as the study subject. According to the random number table method, they were divided into study group(46 cases) and reference group(46 cases). The reference group received basic treatment and language rehabilitation training, and the study group received acupuncture treatment on the basis of the reference group. Before and after treatment, children's gross motor function measure(GMFM), Gesell's overall development assessment, language development quotient(DQ), child development cenler of China(CDCC) and comprehensive function rating scale for disabled children were used to evaluate the clinical efficacy of the two groups. Results: After treatment, the scores of area A, B, C, D and E in study group were higher than those in reference group(*P*<0.05), and the scores of Gesell, DQ, mental development index(MDI) and psychomotor development index(PDI) in study group were higher than those in reference group(*P*<0.05). The scores of function, self-care action, cognitive function, language function and motor function were higher than those of reference group(*P*<0.05), and the total effective rate of study group(95.65%) was higher than that(82.61%) of reference group(*P*<0.05). Conclusion: Language rehabilitation training combined with acupuncture in the treatment of children with cerebral palsy can improve the function of various brain regions and language developmental delay, and improve the clinical efficacy of children.

1. **Yang M, Bai S. Effects of Xingnao Kaiqiao acupuncture combined with language training on language development delay in children with cerebral palsy. Medical Journal of Chinese People's Health 2023;35(14):69-71,75.**

**Title:** Effects of Xingnao Kaiqiao acupuncture combined with language training on language development delay in children with cerebral palsy

**Authors:** Yang M, Bai S

**Abstract:** Objective: To observe effects of Xingnao Kaiqiao acupuncture combined with language training on language development delay in children with cerebral palsy. Methods: A prospective study was conducted on 88 children with cerebral palsy with language development delay admitted to the hospital from July 2019 to July 2022. They were divided into control group and observation group according to the random number table method, 44 cases in each. The control group was treated with language training, while the observation group was treated with Xingnao Kaiqiao acupuncture on the basis of that of the control group. The clinical efficacy, the activities of daily living [activities of daily living scale (ADL)] score, the balance function [Berg balance scale (BBS)] score, the cerebral hemodynamic index levels [cerebral artery mean blood flow velocity (Vm), vascular resistance index (RI), vascular pulsation index (PI)] were compared between the two groups before and after the treatment. Results: The total effective rate of treatment in the observation group was 88.64%, which was higher than 62.36% in the control group, and the difference was statistically significant (*P*<0.05). After the treatment, the ADL and the BBS scores of the observation group were higher than those of the control group, and the differences were statistically significant (*P*<0.05). Further, the Vm and PI of the observation group were higher than those of the control group, the RI was lower than that of the control group, and the differences were statistically significant (*P*<0.05). Conclusions: Xingnao Kaiqiao acupuncture combined with language cognitive training in the treatment of the children with cerebral palsy with language development delay can improve the clinical efficacy, improve the activities of daily living and the balance ability of the children, and improve the cerebral blood flow velocities. Moreover, it is superior to single language training.

1. **Yang Y, Cui YJ, Xu Y, Yang YX, Guo YM, Wang XY, et al. Research Progress on the Advantages of Acupuncture and Moxibustion Rehabilitation Techniques in Rehabilitation Medicine and the Key Mechanisms of Action. Journal of Liaoning University of Traditional Chinese Medicine 2023;25(11):92-96.**

**Title:** Research Progress on the Advantages of Acupuncture and Moxibustion Rehabilitation Techniques in Rehabilitation Medicine and the Key Mechanisms of Action

**Authors:** Yang Y, Cui YJ, Xu Y, Yang YX, Guo YM, Wang XY, Liu YY

**Abstract:** Acupuncture and moxibustion rehabilitation techniques are widely used in various clinical rehabilitation departments,and have become a rehabilitation treatment technique with practical value; In the field of neurological rehabilitation,it can promote the recovery of somatosensory or motor functions, repair nerve damage,promote the repair of speech functions and improve cognitive functions; In postnatal rehabilitation, it is suitable for pelvic floor muscle relaxation, improving postnatal depression and obesity; In paediatric rehabilitation, it can promote the rehabilitation of somatic and speech functions of young children and the improvement of behavioural in paediatric rehabilitation, it is used to promote the rehabilitation of somatic and speech functions and the improvement of behavioural abnormalities in children. In this paper,we review the research progress of the therapeutic advantages and key mechanisms of action of acupuncture and moxibustion rehabilitation techniques in orthopaedic rehabilitation, neurological rehabilitation, cardiopulmonary rehabilitation, gynaecological rehabilitation and paediatric rehabilitation, as well as their specific application techniques. It is expected to provide effective clinical reference for the general rehabilitation medical practitioners.

1. **Zhang Y, Tang YW, Peng YT, Yan Z, Zhou J, Yue ZH. Acupuncture, an effective treatment for post-stroke neurologic dysfunction. Brain Res Bull. 2024 Sep;215:111035.**

**Title:** Acupuncture, an effective treatment for post-stroke neurologic dysfunction

**Authors:** Zhang Y, Tang YW, Peng YT, Yan Z, Zhou J, Yue ZH

**Abstract:** Stroke episodes represent a significant subset of cerebrovascular diseases globally, often resulting in diverse neurological impairments such as hemiparesis, spasticity, dysphagia, sensory dysfunction, cognitive impairment, depression, aphasia, and other sequelae. These dysfunctions markedly diminish patients' quality of life and impose substantial burdens on their families and society. Consequently, the restoration of neurological function post-stroke remains a primary objective of clinical treatment. Acupuncture, a traditional Chinese medicine technique, is endorsed by the World Health Organization (WHO) for stroke treatment due to its distinct advantages in managing cerebrovascular diseases, including ischemic stroke. Numerous clinical studies have substantiated the efficacy of acupuncture in ameliorating neurological dysfunctions following stroke. This review systematically examines the improvements in post-stroke neurological dysfunction attributable to acupuncture treatment and elucidates potential mechanisms of action proposed in recent years. Additionally, this article aims to present novel therapeutic concepts and strategies for the clinical management of post-stroke neurological dysfunction.

1. **Li J, Jiang Y, Li S, Zhuang L. Meta-analysis of clinical efficacy of acupuncture and moxibustion in rehabilitation treatment for post-stroke aphasia. Am J Transl Res. 2024 Oct 15;16(10):5182-5190.**

**Title:** Meta-analysis of clinical efficacy of acupuncture and moxibustion in rehabilitation treatment for post-stroke aphasia

**Authors:** Li J, Jiang Y, Li S, Zhuang L

**Abstract:** Objective: To systematically evaluate the clinical efficacy of traditional Chinese acupuncture and moxibustion in treating post-stroke aphasia through meta-analysis. Methods: Major Chinese and international databases were searched from their inception to December 2023. The search terms included "randomized controlled clinical studies on acupuncture treatment for stroke-related aphasia". After screening by specialists, the selected studies were analyzed using RevMan 5.3 software. Results: A total of 1,900 documents were retrieved, of which 10 studies involving 848 cases met the inclusion criteria and were included in the meta-analysis. The results indicated that traditional Chinese acupuncture could enhance the effectiveness of language rehabilitation training both as a standalone treatment and in combination with other assessment measures. Acupuncture improved the clinical effectiveness of language rehabilitation training for patients with post-stroke aphasia (RR = 3.75, 95% CI [2.54, 2.55], *P* < 0.00001). When combined with language rehabilitation therapy, acupuncture significantly improved patients' comprehension (MD = 0.95, 95% CI [0.89, 1.02], *P* < 0.00001), repetition (MD = 0.82, 95% CI [0.76, 0.88], *P* < 0.00001), reading (MD = 1.95, 95% CI [1.89, 2.01], *P* < 0.00001), and spontaneous speech abilities (MD = 10.90, 95% CI [9.56, 12.23], *P* < 0.00001), compared to the control group. Conclusion: Acupuncture improves the clinical efficacy of language rehabilitation training for stroke-related aphasia. It also enhanced patients' comprehension, repetition, and spontaneous speech abilities more effectively than monotherapy. However, the overall quality of the included studies was low, emphasizing the need for high-quality randomized controlled trials to further validate these findings.

1. **Danniyaer H, Zhou Y. Progress of clinical research on acupuncture in the treatment of paediatric cerebral palsy. Xinjiang Journal of Traditional Chinese Medicine 2022;40(03):104-106.**

**Title:** Progress of clinical research on acupuncture in the treatment of paediatric cerebral palsy

**Authors:** Danniyaer H, Zhou Y

**Abstract:** Cerebral palsy in children is a motor disorder that emerges early in life, characterized by a high incidence of disability and posing a burden on the healthy growth of affected children and their families. With advancements in medicine, the treatment options for cerebral palsy in children have gradually increased, particularly with acupuncture demonstrating exceptional efficacy. The rational application of various acupuncture techniques has proven to be effective in the treatment of cerebral palsy in children.

1. **Liu ZH, Pan PG, Qi YC, Zhao Y, Chai TQ, Tang CZ, et al. Effects of Tongdu Xingshen Acupuncture on Neuronal Cell Apoptosis and Nerve Growth Factor Protein Expression in the Brain Tissue of Young Rats with Cerebral Palsy. Clinical Journal of Traditional Chinese Medicine2010;22(01):36-40+95.**

**Title:** Effects of Tongdu Xingshen Acupuncture on Neuronal Cell Apoptosis and Nerve Growth Factor Protein Expression in the Brain Tissue of Young Rats with Cerebral Palsy

**Authors:** Liu ZH, Pan PG, Qi YC, Zhao Y, Chai TQ, Tang CZ, Wang QY, Yang JJ, Lin JQ

**Abstract:** Objective: To observe the effects of Tongdu Xingshen acupuncture therapy on neuronal apoptosis, nerve growth factor (NGF) protein expression, and limb function in neonatal rats with cerebral palsy, and to analyze whether these effects are related to the timing of acupuncture treatment. Methods: The experiment was conducted in the clean laboratory of the Experimental Animal Center and the Acupuncture-Moxibustion and Tuina School of Guangzhou University of Chinese Medicine between August and October 2005. ① A total of 109 seven-day-old neonatal SD rats were selected. After ligation of the left common carotid artery for 2 hours, the rats were placed in a transparent, airtight container and exposed to hypoxic gas (0.08 oxygen and 0.92 nitrogen by volume) at a rate of 1 L/min in 37°C water. After 2.5 hours, the animals were removed, and survivors were kept warm for an additional hour before behavioral testing. Rats that could not roll over, had abnormal balance, or rotated to the left were considered successful models (72 rats). ② The remaining 24 rats were assigned to the sham surgery group, in which only the left common carotid artery was isolated without ligation or hypoxic treatment. The 72 successfully modeled rats were randomly divided into three groups using a random drawing method: cerebral palsy rats + acupuncture group I, cerebral palsy rats + acupuncture group II, and model group, with 24 rats in each group. The acupuncture points selected were Baihui, Lvgu (penetrating Jiansun) on the affected side, Neiguan, Quchi, Zusanli, and Yongquan. Acupuncture treatment for the cerebral palsy rats + acupuncture group I began 24 hours after modeling, with acupuncture at the limbs only for the first 7 days, followed by the addition of head acupuncture points from the second day onward. Acupuncture treatment for the cerebral palsy rats + acupuncture group II began on the 8th day after modeling, with simultaneous acupuncture at both head and body points. In both groups, acupuncture needles were used for rapid puncturing with slight bleeding at Neiguan and Yongquan, without needle retention. Then, acupuncture was performed at the head and at Quchi and Zusanli. Baihui, Lvgu (penetrating Jiansun), Quchi, and Zusanli were connected to a G6805-II electro-acupuncture apparatus with continuous waves at a frequency of 5-10 Hz for 10 minutes, once a day. The cerebral palsy rats + acupuncture group I received continuous acupuncture for 20 days, while the cerebral palsy rats + acupuncture group II received continuous acupuncture for 13 days. The sham surgery group received acupuncture treatment identical to that of the cerebral palsy rats + acupuncture group I; the model group only underwent modeling without any treatment. ③ On the 7th, 14th, and 21st days after surgery, adhesive tape was attached to the ventral surface of the two forepaws of the rats, and the time taken to remove the tape was recorded. ④ On the 21st day after surgery, 10 rats from each group were randomly selected for the following tests: neuronal apoptosis in the frontal cortex and hippocampus and NGF expression in the hippocampus were measured using the terminal deoxynucleotidyl transferase dUTP nick end labeling (TUNEL) method and immunohistochemical staining, respectively. The number of apoptotic neurons and NGF-positive cells was counted under a light microscope (magnification, ×400) using an image analyzer. Results: Of the 109 rats included, 13 were lost due to modeling failure, and there were no deaths in the sham surgery group. Seven days after modeling, 2, 5, and 3 rats died in the cerebral palsy rats + acupuncture group I, cerebral palsy rats + acupuncture group II, and model group, respectively; between 7 and 14 days after modeling, 2, 2, and 4 rats died in these three groups, respectively; and between 14 and 21 days after modeling, 0, 0, and 2 rats died in these three groups, respectively. ① Neuronal apoptosis in the hippocampus and frontal cortex of rats: The number of TUNEL-positive cells in the hippocampus and frontal cortex was significantly reduced in both acupuncture treatment groups compared with the model group (*P*<0.05). Additionally, the count of apoptotic neurons was significantly lower in the cerebral palsy rats + acupuncture group I than in the cerebral palsy rats + acupuncture group II (*P*<0.01). ② NGF protein expression in hippocampal neurons: NGF protein expression was significantly enhanced in both acupuncture treatment groups, with a greater number and intensity of NGF-positive cells compared with the sham surgery and model groups (*P*<0.01). The cerebral palsy rats + acupuncture group I had significantly more NGF-positive cells than the cerebral palsy rats + acupuncture group II (*P*<0.01). ③ On the 7th day after surgery, the time taken to remove the adhesive tape was significantly shorter in the cerebral palsy rats + acupuncture group I than in the model group (*P*<0.01); on the 14th day after surgery, the model group took significantly longer than the other three groups (*P*<0.05 or 0.01); and on the 21st day after surgery, the forelimb function of both acupuncture groups and the model group improved significantly, with the cerebral palsy rats + acupuncture group I nearly returning to normal levels (no significant difference compared with the sham surgery group), but the model group still took significantly longer than the cerebral palsy rats + acupuncture group I and the sham surgery group (*P*<0.01). Conclusion: Acupuncture can inhibit neuronal apoptosis, enhance NGF expression in the brain tissue of neonatal rats with cerebral palsy, and improve limb function. It has a certain protective effect on brain tissue damage in neonatal rats with cerebral palsy, and earlier intervention results in better outcomes.

1. **Zhang H, Gao J, Wang M, Yu X, Lv X, Deng H, et al. Effects of scalp electroacupuncture on the PI3K/Akt signalling pathway and apoptosis of hippocampal neurons in a rat model of cerebral palsy. Acupunct Med. 2018 Apr;36(2):96-102.**

**Title:** Effects of scalp electroacupuncture on the PI3K/Akt signalling pathway and apoptosis of hippocampal neurons in a rat model of cerebral palsy

**Authors:** Zhang H, Gao J, Wang M, Yu X, Lv X, Deng H, Fan X, Chen K

**Abstract:** Background: Substantial evidence from clinical reports has established that most cerebral palsy (CP) patients benefit from a comprehensive rehabilitation exercise training programme. Such advances are enhanced when scalp electroacupuncture (EA), applied at a location corresponding to the projection of the motor area, is combined with rehabilitation exercise training. However, little information exists regarding the mechanistic basis for these effects. Objective: To examine whether EA stimulation within the scalp projection location of the motor area can inhibit apoptosis of hippocampal neurons by regulating the PI3k/Akt signalling pathway in a rat model of CP. Methods: Fifty male Sprague-Dawley rats underwent surgical modelling of CP. Five were used to confirm successful establishment of the model and the remaining 45 rats were randomly divided into one of three groups that remained untreated (CP group, n=15) or received EA treatment alone (CP+EA group, n=15) or EA in combination with a PI3K/Akt inhibitor (CP+EA+LY294002 group, n=15)). An otherwise healthy negative control group of rats undergoing sham surgery was also included (Control group, n=15). In the CP+EA and CP+EA+LY294002 groups, EA was applied to the scalp surface at alocation corresponding to the projection of the motor area. Basso, Beattie and Bresnahan (BBB) locomotor scores, hippocampal protein expression of Akt and p-Akt (by Western blot analysis) and neuronal apoptosis in hippocampal tissue (by histopathology) were assessed at 7, 14 and 21 days post-CP induction. Results: CP rats receiving scalp EA treatment demonstrated improved behavioural scores, less hippocampal neuronal apoptosis and higher expression levels of Akt and p-Akt (*p*<0.05) at all time points studied compared with untreated CP rats. There were no significant differences observed between CP+EA+LY294002 and untreated CP model groups. Conclusions: The effects of scalp EA on the PI3K/ Akt signalling pathway may represent one of the mechanisms involved in the inhibition of hippocampal neuronal apoptosis and improvement of deficits associated with CP in a rat model.

1. **Zhou L, Zhang XY, Zhang HX, Zou R, Liu YN, Wang Q. Scalp-acupuncture induced the proliferation and differentiation of neural stem cell. Stroke and Nervous Diseases2011;18(03):138-141.**

**Title:** Scalp-acupuncture induced the proliferation and differentiation of neural stem cell

**Authors:** Zhou L, Zhang XY, Zhang HX, Zou R, Liu YN, Wang Q

**Abstract:** objective: To observe the change of proliferation and differentiation of neural stem cells (NSCs) of acute cerebral ischemia/reperfusion injury and the effects of intervention of scalp-acupuncture. Methods: 70 wistar rats were randomized into sham group(10), model group(30) and SA group(30). Model group and SA group with middle cerebral artery occlusion (MCAO) were divided into 3 subgroups respectively according to ischemic time (7d、14d、28d), and each subgroups with 10 rats SA rats were treated after MCAO experiments (once a day until death). The abdoman of rat of subgroups wrer injected bromodeoxyuridine (BrdU) solution. Neurological severity score (NSS) was applied for each phase point rats. Last the BrdU positive cells and the BrdU/PSA-NCAM positive cells of each set was observed with immunofluorescence assay (IFA). Results: On NSS at each phase point: SA group demonstrated obviously declined at 28d comparing with that of model group(*P*<0. 05). On observation of positive cells at each group, model group and SA group significantly increased in contrast to sham group. A clearly difference can be seen on observation of positive cells of model group and SA group at each phase point. Conclusions: SA can stimulate the proliferation and the differentiation of NSCs and is helpful for the reparation of neurologic, which may become the theoretical basis of SA intreating cerebral ischemia.

1. **Chen JK, Liu ZH. New advances in acupuncture for neural development and repair in the brain. Journal of Changchun University of Chinese2012;28(04):756.**

**Title:** New advances in acupuncture for neural development and repair in the brain

**Authors:** Chen JK, Liu ZH

**Abstract:** Research indicates that acupuncture can facilitate neural development and repair in the brain. In experimental animals, studies have demonstrated that acupuncture promotes the expression of various neurotrophic factors and growth factors, thereby enhancing the development, repair, and regeneration of brain neurons. It also stimulates the proliferation and differentiation of neural stem cells, fosters the sprouting, growth, and elongation of axons, and promotes the formation of myelin sheaths, ultimately contributing to the establishment of neural networks. Furthermore, acupuncture exerts beneficial regulatory effects on astrocyte proliferation. Additionally, acupuncture promotes angiogenesis in the brain and modulates various factors that influence cerebrovascular dilation and constriction, thereby improving cerebral microcirculation and increasing cerebral blood perfusion. It also protects mitochondria, increases the content of glucose transporter receptors in brain tissue, enhances cerebral energy metabolism, and boosts neuronal vitality. Clinically, studies have shown that acupuncture can inhibit abnormal cerebral discharges, thereby reducing the damage to brain cells caused by such discharges. It promotes the repair of damaged auditory neural pathways and improves neural conduction in the brain. Moreover, acupuncture increases cerebral blood supply and enhances energy metabolism in brain cells.

1. **Huang YT, Liang HC. Observation on the Curative Effect of Head Acupuncture Combined with Rehabilitation Training in Children with Mental Retardation. Journal of External Therapy of Traditional Chinese Medicine 2023;32(03):1-3.**

**Title:** Observation on the Curative Effect of Head Acupuncture Combined with Rehabilitation Training in Children with Mental Retardation

**Authors:** Huang YT, Liang HC

**Abstract:** Objective: To observe the clinical efficacy of head acupuncture combined with rehabilitation training in the treatment of children with mental retardation. Methods: Sixty children with mental retardation treated in our hospital from December 2020 to December 2021 were selected as study subjects. They were randomly divided into a control group (n=30) and an observation group (n=30) using a random number table method. The control group received rehabilitation training alone, while the observation group received head acupuncture combined with rehabilitation training. The Chinese-Wechsler Intelligence Scale for Children (C-WISC), Gesell Developmental Scales, and Social Maturity Scale for Infants and Juveniles (S-M) were used to compare improvements in IQ, developmental levels, and social functioning between the two groups. Results: After treatment, the observation group had higher scores for Verbal IQ (VIQ), Performance IQ (PIQ), and Full-Scale IQ (FIQ) than the control group, with statistically significant differences (*P*<0.05). The observation group also scored higher in personal-social skills, language, fine motor skills, adaptability, and gross motor skills than the control group, with statistically significant differences (*P*<0.05). Additionally, the observation group scored higher in independent living, motor skills, schoolwork, social interaction, participation in group activities, and self-management than the control group, with statistically significant differences (*P*<0.05). Conclusion: The implementation of head acupuncture combined with rehabilitation training in children with mental retardation can significantly improve IQ, promote physical and mental development, and enhance social functioning.

1. **Liu Q, Hu W, Lv LL, Fu XM, Chen LN. Clinical Study on Scalp Acupuncture Combined with Collective Language Training for Delayed Language Development in Children. New Chinese Medicine 2024;56(01):155-159.**

**Title:** Clinical Study on Scalp Acupuncture Combined with Collective Language Training for Delayed Language Development in Children

**Authors:** Liu Q, Hu W, Lv LL, Fu XM, Chen LN

**Abstract:** Objective: To observe the clinical effect of scalp acupuncture combined with collective language training for delayed language development in children. Methods: A total of 60 cases of children with delayed language development were divided into the treatment group and the control group according to the random number table method,with 30 cases in each group. The control group was given collective language training,and the treatment group was additionally treated with scalp acupuncture based on the treatment of the control group. Traditional Chinese medicine(TCM) syndrome scores and Gesell Developmental Schedules(GDS), S-S language retardation evaluation method(S-S) score, and Chinese functional communication profile(CFCP) score before and after treatment as well as clinical effects were compared between the two groups. Results: After treatment,the total effective rate was 96.67% in the treatment group, higher than that of 80.00% in the control group(*P*<0.05). After treatment, the scores of each domain(language,adaptability,fine motor skills,gross motor skills, and personal social activity) of GDS in the two groups were increased when compared with those before treatment(*P*<0.05), and the scores of domain after treatment in the treatment group were higher than those in the control group(*P*<0.05). After treatment,TCM syndrome scores in the two groups were decreased when compared with those before treatment(*P*<0.05), and the scores of S-S and CFCP were increased(*P*<0.05); TCM syndrome scores in the treatment group were lower than those in the control group(*P*<0.05), and the scores of S-S and CFCP in the treatment group were higher than those in the control group(*P*<0.05). Conclusion: Scalp acupuncture combined with collective language training has a significant curative effect in the treatment of delayed language development in children,and can effectively relieve clinical symptoms and improve the language function and communication skills.

**[83] Tan F, Wang J, Chen J, Gu Y, Zhan J, Gu MH, et al. Effect of Electroacupuncture on the Expression of Hippocampal e NSCs in MCAO Model Rats. Chinese Journal of Integrated Traditional and Western Medicine 2017;37(2):198-203.**

**Title:** Effect of Electroacupuncture on the Expression of Hippocampal e NSCs in MCAO Model Rats

**Authors:** Tan F, Wang J, Chen J, Gu Y, Zhan J, Gu MH, Tan JQ, Liang YG, Liu XF

**Abstract:** Objective: To observe the effects of electroacupuncture (EA) on hippocampal endogenous neural stem cells (e NSCs) expression of middle cerebral artery occlusion (MCAO) model rats after cerebral ischemiareperfusion (I / R) at different time points, and to observe possible mechanisms of EA for keeping away fromdamage in acute cerebral infarction (ACI). Methods: MCAO model was prepared in male SPF grade SD rats by suture method. Totally 90 rats were divided into the sham-operated group, the model group, and the EA group according to random number table, 30 in each group. Rats in the sham-operated group only received surgical trauma. Rats in the model group only received MCAO I / R injury. Rats in the EA group received EA at Baihui ( DU20) and Dazhui ( DU14) , once per day, 30 min each time. Nerve defects of rats were tested by neural function defect scale at day 1, 7, 14 of treatment, respectively. Meanwhile, 6rats were executed randomly from each group. Their hippocampus tissues were isolated. Then the proliferation and differentiation expression of e NSCs in the hippocampus area were detected by immunofluorescence method. Results: (1) The scores of nerve function defect scale: The scores of the model group increased at day 1, 7, 14 of treatment, being higher as compared with those of the sham-operated group (*P*<0.05).The scores of the EA group were lower than those of the model group at day 1, 7, 14 of treatment (*P*<0.05). (2) The expression of Brd U positive cells: Compared with the sham-operated group, the expression of Brd U positive cells in the model group were increased at day 1, 7, 14 of treatment (*P*<0.05) . Compared with the model group at each time points, the expression of Brd U positive cells in the EA group were increased more at day 1, 7, 14 of treatment (*P*<0.05). (3) The expression of Nestin positive cells: The expression of Nestin positive cells were increased more in the model group than in the sham-operated group at day 1, 7, 14 of treatment (*P*<0.05). Compared with the model group, the expression of Nestin positive cells increased more in the EA group, but only with statistical difference at day 7 of treatment (*P*<0.05). (4) the expression of DCX positive cells: the expression of DCX positive cells were increased more in the model group than in the sham-operated group at day 1 and 7 of treatment (*P*<0.05). Compared with the model group, the expression of DCX positive cells were increased more in the EA group at day 7and 14 of treatment (*P*<0.05). (5) the expression of Neu N positive cells: The expression Neu N of positive cells were increased more in the model group than in the sham-operated group at day 1, 7, and 14 of treatment, but only with statistical difference at day 14 of treatment (*P*<0.05). Compared with the model group, the expression of Neu N positive cells were increased more obviously, but only with statistical difference at day 1 and 14 of treatment (*P*<0.05). (6) the expression of GFAP positive cells: The expression of GFAP positive cells increased more obviously in the model group than in the sham-operated group at day 1, 7, and 14 of treatment (*P*<0.05). Compared with the model group, the expression of GFAP positive cells were not obviously increased in the EA group, but only with statistical difference at day14 of treatment (*P*<0.05). Conclusions: The proliferation and differentiation of e NSCs exist in the hippocampus area after cerebral I / R in MCAO model rats. EA could improve the recovery of damaged nerve function. Its possible mechanism might lie in that EA could promote the proliferation and differentiation of eNSCs in hippocampus area, inhibit excessive differentiation of e NSCs into astrocytes, promote differentiation of eNSCs into neurons, and improve regeneration of nerve cells.

**[84] Wang B, Zhang XM, Wu S, Huang W, Li D, Lu W, et al. Effect on p53 and caspase-3 of hippocampal neuron in the rats with cerebral ischemiareperfusion injury treated with electroacupuncture preconditioning at the acupoints on the basis of biao and ben relationship. Chinese Acupuncture & Moxibustion 2019;39(9):957-962.**

**Title:** Effect on p53 and caspase-3 of hippocampal neuron in the rats with cerebral ischemiareperfusion injury treated with electroacupuncture preconditioning at the acupoints on the basis of biao and ben relationship

**Authors:** Wang B, Zhang XM, Wu S, Huang W, Li D, Lu W, Xie J

**Abstract:** Objective: To explore the protective effect and apoptosis-related mechanism of electroacupuncture (EA) preconditioning in the rats with cerebral ischemia-reperfusion injury. Methods: Sixty male SD rats, 3 months old, at SPF grade were randomized into a sham-operation group, an ischemia-reperfusion group and an EA preconditioning group, 20 rats in each one. In the ischemia-reperfusion group and EA preconditioning group, the modified MCAO suture-occlusion method was adopted to exert ischemia for 2h and reperfusion for 3h, and thus, the models of focal cerebral ischemiareperfusion injury were prepared on the right side. In the sham-operation group, the right common carotid artery was separated and no more management was given. In the EA preconditioning group, EA at “Baihui” (GV 20) , “Shenshu” (BL 23) and “Sanyinjiao” (SP 6) was provided before modeling, with disperse-dense wave, at 2 Hz/100 Hz, 1 mA in intensity. The stimulation for 15 min was taken as one unit (meaning electric stimulation for 10 min and needle retaining for 5 min without electric stimulation). Such preconditioning was repeated continuously for 4 times, totally for 1h. The neuroethologic condition was assessed in 3h of reperfusion in each group. TTC staining method was used to determine the percentage of cerebral infarction zone, TUNEL method was to determine the apoptosis index (AI) in hippocampal neuron and the immunohistochemical method (IHC) was to determine the protein expression of p53 and caspase-3. Results: Compared with the sham-operation group, the neuroethologic score, the percentage of cerebral infarction zone and neuronal AI were all increased obviously in the ischemia-reperfusion group (all *P*<0.01). Compared with the ischemia-reperfusion group, the neuroethologic score, the percentage of cerebral infarction zone and neuronal AI were all reduced obviously in the EA preconditioning group (all *P*<0.01). p53's nuclei and caspase-3's cytoplasms were stained. The positive cells of both of them were brown-yellow in color. In the sham-operation group, the structure of the right hippocampal CA3 neurons of rats was clear, with few positive cells. In the ischemia-perfusion group, the positive expressions of p53 and caspase-3 in the right hippocampal CA3 were increased obviously (*P*<0.01). Compared with the ischemia-reperfusion group, the positive expressions of caspase-3 and p53 in the right hippocampal CA3 were significantly reduced in the EA preconditioning group (*P*<0.01). Conclusion: Electroacupuncture preconditioning relieves ischemic injury in brain tissue of rats probably through inhibiting the expressions of p53 and caspase-3 to resisting neuronal apoptosis.

**[85] Tao WT, Chen JJ, Zheng Z. Protective effect of electroacupuncture preconditioning at Baihui, Shenshu and Sanyinjiao acupoints on cerebral ischemia-reperfusion injury in rats. Journal of Hubei University of Chinese Medicine 2023;25(1):15-20.**

**Title:** Protective effect of electroacupuncture preconditioning at Baihui, Shenshu and Sanyinjiao acupoints on cerebral ischemia-reperfusion injury in rats

**Authors:** Tao WT, Chen JJ, Zheng Z

**Abstract:** Objective: To observe the neurological function, pathological morphology of brain tissue and expression of apoptosis proteins of the ischemia-reperfusion injury rats which were pre-treated with electroacupuncture (EA) at Baihui, Shenshu and Sanyinjiao. Methods: Fifty-six SPF grade SD male rats were randomly divided into four groups,normal group, model group, EA pretreatment group, and EA control group, 14 rats each. Normal group did not receive any treatment;model group was treated with middle cerebral artery occlusion (MCAO) suture method to prepare the right focal cerebral ischemiareperfusion injury model in rats,with ischemia for 2h and reperfusion for 6h; rats were pretreated by EA at Baihui, Shenshu and Sanyinjiao acupoints for 5 consecutive days, and then cerebral ischemia-reperfusion injury was modeled on the 6th day. The rest of the treatments were the same as EA pretreatment group. Longa-Bederson neurological function score was used to evaluate the degree of neurological damage in each group, TTC staining was used to observe the volume of cerebral infarction, HE staining was used to observe the neuronal changes in hippocampal CA3 area, and immunohistochemistry and Western Blot were used to detect hippocampal apoptosis-related proteins, Bax, Bcl-2 and Cleaved-caspase3 expression levels. Results: Compared with normal group,the neurological function score of model group significantly increased,the cerebral infarction volume expanded, the arrangement of nerve cells in the hippocampal CA3 region disordered, a large number of cells were necrotic,the expression of apoptosis-related proteins Bax and Cleaved-caspase3 increased,and the expression of Bcl-2 decreased;compared with model group, neurological function score in EA preconditioning group significantly decreased, cerebral infarction volume reduced,nerve cell injury in hippocampal CA3 area reversed, Bcl-2 increased and Bax and Cleaved-caspase3 decreased; while EA control group showed no significant difference compared with normal group. Conclusion: EA preconditioning might play a protective effect on cerebral ischemia-reperfusion injury in rats, it significantly ameliorate the neurological damages, reduce the volume of cerebral infarction and play an anti-apoptotic effect in the brain related to the acupoint specificity.

**[86] Li WH, Yu J, Lin YP, Tan X, Song Y. Effect of electroacupuncture at Neiguan (PC 6) and Baihui (GV 20) on CHOP and caspase-12 gene expressions in rats after ischemia-reperfusion injury. Journal of Acupuncture and Tuina Science 2017;15(1):8-13.**

**Title:** Effect of electroacupuncture at Neiguan (PC 6) and Baihui (GV 20) on CHOP and caspase-12 gene expressions in rats after ischemia-reperfusion injury

**Authors:** Li WH, Yu J, Lin YP, Tan X, Song Y

**Abstract:** Objective: To investigate the effects of electroacupuncture (EA) at Neiguan (PC 6) and Baihui (GV 20) by observing the changes of CCAAT/enhancer-binding protein (C/EBP) homologous protein (CHOP) and caspase-12 gene expressions in rats after cerebral ischemia-reperfusion injury (IRI), and explore whether the apoptosis pathway of endoplasmic reticulum stress (ERS) is involved in the protective mechanisms of EA. Methods: Sixty rats were randomly assigned to five groups (12 in each group): a normal control group (group A) , a sham-operation group (group B) , an operation group (group C), an Edaravone group (group D) and an EA group (group E). The cerebral IRI rat model was induced by middle cerebral artery occlusion (MCAO) using intraluminal monofilament. 2, 3, 5-triphenyl tetrazolium chloride (TTC) staining was adopted in the measurement of cerebral infarction volume. Real-time polymerase chain reaction (RT-PCR) was used to determine them RNA expressions of CHOP and caspase-12. Results: Compared with group A and group B, the volume of cerebral infarction and m RNA expressions of CHOP and caspase-12 in group C, group D and group E were increased, with statistical significances (*P*<0.05 or *P*<0.01); compared with group C, the volume of cerebral infarction and m RNA expressions of CHOP and caspase-12 in group D and group E were decreased significantly (*P*<0.05 or *P*<0.01); there were no significant differences between group D and group E in comparing the above items (*P*>0.05) . Conclusion: EA at Neiguan (PC 6) and Baihui (GV 20) can effectively suppress the volume of cerebral infarction. Furthermore, the underlying mechanism of EA at Neiguan (PC 6) and Baihui (GV 20) is possibly related to the down-regulation of CHOP and caspase-12 mRNA expressions, so as to decrease cell apoptosis.
